# Development of Prostate-Specific Lysosome-Targeting Degraders

**DOI:** 10.1021/jacs.5c18594

**Published:** 2026-02-11

**Authors:** Deqin Cai, Xuankun Chen, Yaxian Zhou, Malick Bio Idrissou, Reinier Hernandez, Weiping Tang

**Affiliations:** † Lachman Institute of Pharmaceutical Development, School of Pharmacy, 5228 University of Wisconsin-Madison, Madison, Wisconsin 53705, United States; ‡ Department of Chemistry, University of Wisconsin–Madison, Madison, Wisconsin 53706, United States; § Department of Medical Physics, University of Wisconsin School of Medicine and Public Health, Madison, Wisconsin 53705, United States; ∥ Department of Radiology, University of Wisconsin School of Medicine and Public Health, Madison, Wisconsin 53792, United States

## Abstract

Targeted protein degradation (TPD) technologies have emerged as transformative therapeutic modality for treating cancers and other diseases. While significant progress has been achieved in intracellular protein degradation, degradation of membrane proteins and extracellular targets remains in an early stage. In this study, we developed a prostate-specific lysosome-targeting degradation strategy using a prostate-specific membrane antigen (PSMA) as a lysosome-targeting receptor (LTR). We demonstrated that both extracellular and membrane proteins can be selectively degraded in prostate cancer cells via the lysosome pathway. These PSMA TArgeting Chimeras (PTACs) were shown to facilitate lysosomal degradation in a selective, potent, rapid, and sustained manner. Notably, **Ctx-L3** and **Atz-L5** exhibited exceptional degradation potencies in LNCaP cells, with DC_50_ values of 4.3 pM for EGFR and 2 pM for PD-L1, respectively—among the most potent degraders reported to date. Furthermore, the application of PTACs to degrade PD-L1, using both antibody- and small-molecule-based formats, highlights the versatility of this platform. Collectively, this work advances the application of TPD technology and offers promising avenues for precision medicine in prostate-related diseases.

## Graphical abstract

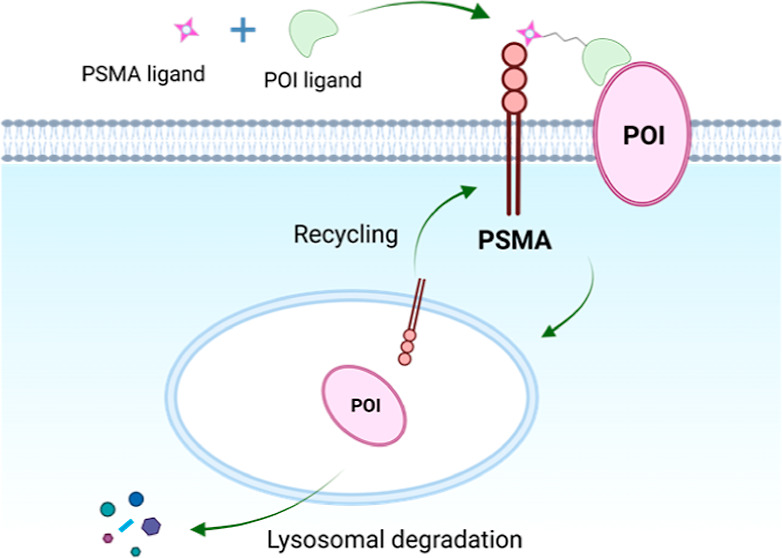

## Introduction

Protein degradation is essential for maintaining cellular homeostasis, primarily mediated by the ubiquitin-proteasome system (UPS) and the lysosomal degradation pathway.^1–3^ Proteolysis targeting chimeras (PROTACs) and monovalent molecular glue degraders leverage the UPS by inducing the proximity between protein of interest (POI) and E3 ligase in cytoplasm, leading to POI ubiquitination and subsequent proteasomal degradation.^4^ PROTAC ARV-471 has advanced to a phase III clinical trials for the treatment of advanced breast cancer.^5,6^ Unlike classical occupation-driven enzyme inhibition or ligand blocking, TPD can eliminate the entire protein including both the enzymatic and nonenzymatic functions.^7,8^ This novel mechanism of action holds the potential to overcome resistance to conventional treatments and provides a promising avenue for discovering drugs targeting challenging or undruggable proteins.^9^ However, the proteasomal pathway is limited to degrading POIs with cytosolic domains.

To extend the targeted protein degradation paradigm to extracellular proteins, lysosome-targeting chimeras (LYTACs) have been developed.^10–13^ LYTACs are designed to bind extracellular or membrane proteins and direct them to lysosomes for degradation via lysosomal targeting receptor (LTR)-mediated endocytosis, offering a complementary approach to proteasomal degradation. Several LTRs have been identified,^14^ including cation-independent mannose-6-phosphate receptor (CI-M6PR),^10,15–20^ asialoglycoprotein receptor (ASGPR),^11–13^ macrophage galactose-type lectin 1 (MGL1),^21^ integrin,^22–24^ scavenger receptors (SRs),^25^ cytokine receptors,^26^ mannose receptor (CD206),^27^ glucagon-like peptide-1 receptor (GLP-1R),^28^ low-density lipoprotein receptor-related protein (LRP-1),^29^ glucose transporter 1 (GLUT1),^30^ transferrin receptor,^31^ and folate receptor.^32^ Other innovative strategies^33,34^ have also been developed for extracellular or transmembrane protein degradation, such as lysosome-based antibody-based PROTAC (AbTAC),^35^ proteasome and lysosome-based proteolysis-targeting antibodies (PROTABs),^36^ covalent nanobody-based PROTAC strategy (GlueTAC),^37^ and multivalent nanobody-targeting chimeras (mNbTACs).^38^

Despite these significant advancements, most LTRs identified thus far are expressed across multiple tissues, with ASGPR being the notable exception, as reported by us and others.^11–13^ This lack of specificity poses challenges in minimizing on-target and off-tissue side effects and achieving precise therapeutic outcomes. Developing degraders with high tissue specificity is crucial to maximizing therapeutic efficacy while minimizing unintended side effects. This is particularly important in targeting diseases where localized protein degradation is essential, such as tissue-specific cancers.

Prostate-specific membrane antigen (PSMA) is overexpressed in most of prostate cancer cells, correlating with disease progression and poor prognosis, while its expression in normal prostate tissue is minimal, often undetectable.^39^ PSMA is a transmembrane glycoprotein, consisting of a 24-amino acid transmembrane region, a 19-amino acid N-terminal cytoplasmic sequence, and a large extracellular domain comprising 707 amino acids.^40,41^ PSMA facilitates the internalization of targeting molecules and their payloads, making it as an ideal molecular target for diagnostic imaging and targeted therapies.^39,42–45^ Notably, PSMA has already been clinically validated through the FDA approval of [^68^Ga]­Ga-PSMA-11 as a PET imaging agent and [^177^Lu]­Lu-PSMA-617 (Pluvicto) as a targeted radioligand therapy for advanced prostate cancer.

In this study, we report the development of PSMA TArgeting Chimeras (PTACs) as a versatile platform for selective degradation of extracellular and membrane proteins in PSMA-positive prostate cancer cells (Figure 1). The work started with the uptake of soluble antibiotin mediated by biotinylated PSMA ligands. The utility of the platform was subsequently demonstrated through the selective degradation of the membrane protein epidermal growth factor receptor (EGFR). The PTAC approach was further extended to the degradation of PD-L1 in both antibody- and small molecule-based formats. Antibody-based PTACs were prepared by conjugating the PSMA ligands Glu-urea-Lys (EuK)^46,47^ to antibody targeting the POI, while small-molecule-based PTACs were synthesized by linking the PD-L1 ligand BMS-8 with the EuK ligand. Chimeras incorporating PSMA ligands with varying affinities were evaluated to assess their impact on the POI degradation efficiency. Our results suggest that PTACs hold significant promise as a technology for both research purposes and therapeutic applications in the study and treatment of prostate-related diseases.

**Figure 1:**
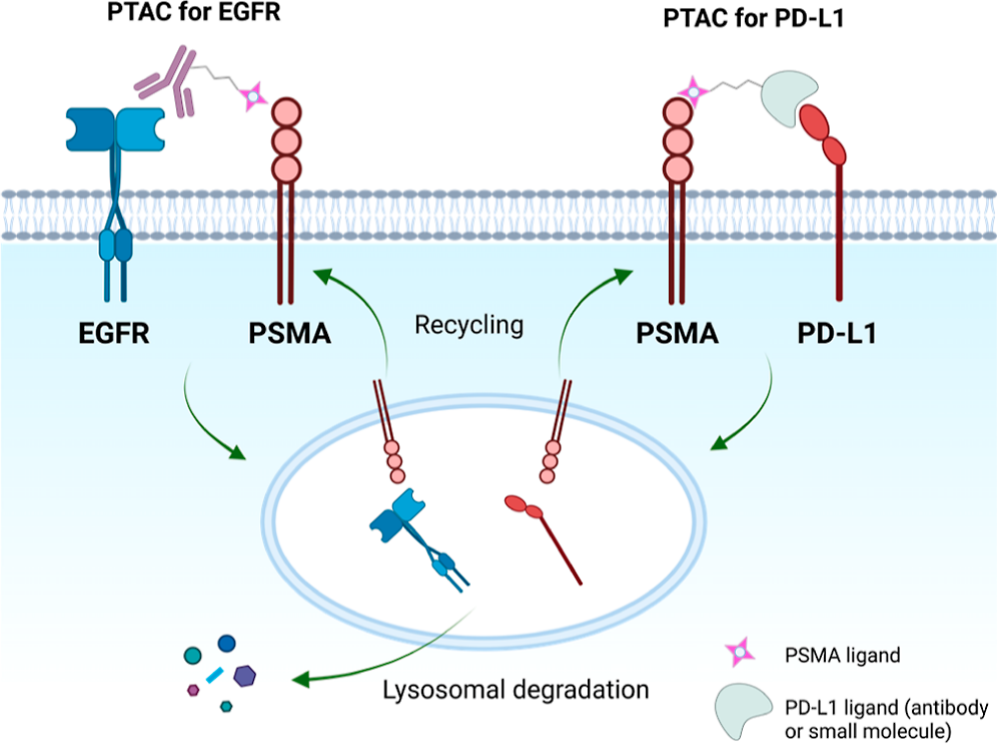
Schematic illustration of PSMA TArgeting Chimeras (PTACs) used in the current study for TPD of membrane proteins EGFR and PD-L1 via lysosomal degradation pathways.

## Results and Discussion

### Uptake of Fluorescent Antibiotin-647 by a Biotinylated PSMA Ligand with Various Linkers in Different Prostate Cancer Cell Lines

We began with investigating the uptake of antibiotin-647 using biotinylated PSMA ligands. Five distinct biotinylated PSMA ligands were designed and synthesized, as shown in Figure 2. The synthetic route and procedure are provided in the Supporting Information. Initially, biotinylated PSMA ligands with linkers of three different lengths were synthesized: **L1-biotin**, **L2-biotin**, and **L3-biotin**. No uptake was observed with **L1-biotin** and **L2-biotin**, while uptake by **L3-biotin** bearing a longer linker was observed only in PC3-PIP cells, a PSMA-overexpressing variant of the PC3 cell line, in a dose-dependent manner (Figures 3a and S1).

**Figure 2:**
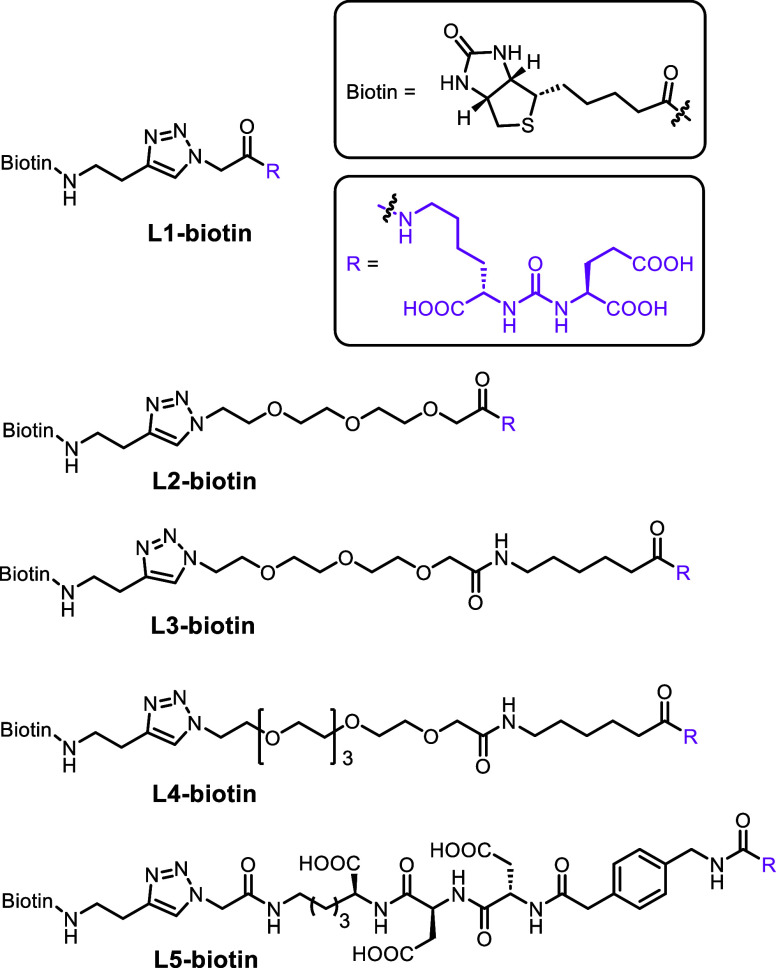
Biotinylated PSMA ligands with various linkers.

**Figure 3:**
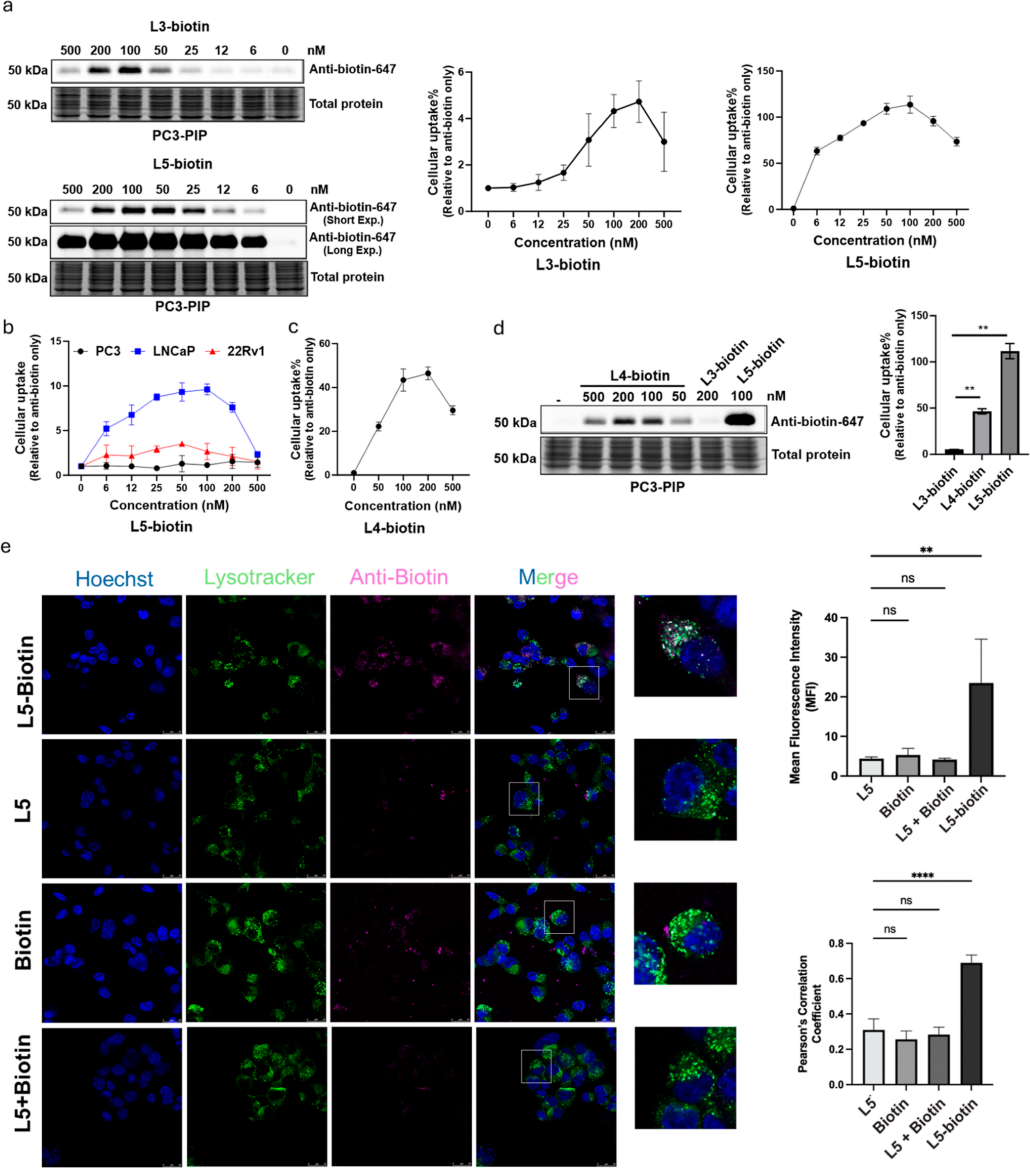
Uptake of fluorescent antibiotin-647 in prostate cancer cells treated with **L3-biotin**, **L4-biotin**, or **L5-biotin**. (a) Dose–response of antibiotin-647 uptake after 24 h of treatment with **L3-biotin** or **L5-biotin** in PC3-PIP cells (*n* = 3). (b) Dose–response of antibiotin-647 uptake after 24 h of treatment with **L5-biotin** in LNCaP, 22Rv1, and PC3 cells (*n* = 3). (c) Dose–response of antibiotin-647 uptake after 24 h of treatment with **L4-biotin** in PC3-PIP cells (*n* = 3). (d) The maximum uptake of antibiotin-647 (24 h) treated with **L3-biotin** (200 nM), **L4-biotin** (200 nM), and **L5-biotin** (100 nM) in PC3-PIP cells (*n* = 3). (e) Cellular uptake and lysosome colocalization of antibiotin-647 (100 nM) in the presence of L5-biotin (100 nM), free L5 (100 nM), biotin (100 nM), and biotin (100 nM) + free L5 (100 nM) in LNCaP cells for 4 h by immunofluorescent staining. Scale bar: 25 μm. The intracellular fluorescence intensity is presented as mean fluorescence intensity (MFI) (*n* = 9). The colocalization was analyzed by Pearson’s correlation coefficients (*n* = 9). Data are presented as mean ± SD. The statistical significance was assessed using an unpaired two-tailed *t*-test, **P <0.01, ****P <0.0001, NS: not significant.

These observations are not surprising, since it has been shown that the linker attached to the PSMA ligand could significantly affect its binding affinity to PSMA.^48–50^ Notably, a ∼20 Å deep funnel exists between the ligand binding site and the protein surface.^51^ In addition to linker length, other parameters can also significantly enhance the binding affinity. For example, a PSMA ligand that contains a short peptide Asp-Asp-Cys and an aromatic motif exhibits a 30-fold higher binding affinity to PMPA,^52^ a known PSMA ligand with a *K*_*i*_ of 0.3 nM.^53^ In contrast, the PSMA ligand bearing a linker of a linear aliphatic motif has a *K*_*i*_ value of only 8.84 nM.^54^ Based on these, we synthesized biotinylated PSMA ligand **L5-biotin**, with a short peptide Asp-Asp-Lys and an aromatic motif. The uptake of antibiotin-647 by **L5-biotin** was clearly observed in all PSMA positive cell lines: PC3-PIP, LNCaP, and 22Rv1 (Figure 3a,b). The uptake efficiency is dose-dependent and corelated to the PSMA expression level (LNCaP > 22Rv1 > PC3, Figures 3b, S2, and S3).

To further investigate the effect of linker structure on uptake efficiency, we synthesized **L4-biotin**, which has the same linker length as **L5-biotin**. **L4-biotin** exhibited significantly better uptake efficiency than the previous linear structure, **L3-biotin**, in a dose-dependent manner (Figure 3c). The improvement is likely due to the longer linker present in **L4-biotin**. However, at a concentration of 200 nM, **L4-biotin** achieved only ∼40% of the uptake of **L5-biotin** (Figure 3a,d), which was further confirmed by co-localization data (Figure 3e). Remarkably, uptake induced by **L5-biotin** could be observed as low as at 6 nM. Additionally, a hook effect^55^ was observed for all three compounds: **L3-biotin**, **L4-biotin**, and **L5-biotin**. Notably, **L5-biotin**, with its higher binding affinity, exhibits a broader effective concentration range.

### Degradation of EGFR Mediated by PTACs

Given the differential PSMA expression across prostate cancer cell lines, it is important to note that PC3-PIP cells (engineered to overexpress PSMA) exhibit the highest PSMA levels, followed by LNCaP (endogenous expression) and 22Rv1 (low expression), while PC3 cells are essentially PSMA-negative. In this study, we initially employed PC3-PIP cells to establish mechanistic insights and subsequently focused on LNCaP cells to ensure greater clinical relevance. After demonstrating the uptake efficiency of a model target protein mediated by PTACs bearing PSMA ligands with varying binding affinities, we initiated our investigation into the development of degraders targeting therapeutically relevant proteins, such as EGFR. EGFR is an oncogenic driver overexpressed in many cancers, including prostate cancer. Commercially available reagent N_3_–PEG_12_-C_3_–OSu was used to conjugate with cetuximab (Ctx), an FDA-approved EGFR blocking antibody, via reaction with lysine residues on the antibody to produce azide-modified Ctx. **L3-DBCO** and **L5-DBCO** were then reacted with azide-modified Ctx to generate **Ctx-L3** and **Ctx-L5**, respectively, through the copper-free Click reaction (Figures 4 and S4–S5). Interestingly, **Ctx-L3** and **Ctx-L5** showed similar degradation efficiencies for EGFR across all three PSMA positive cell lines. Minimal degradation of EGFR was observed in PSMA-negative PC3 cells (Figures 5a and S6). The *D*_max_ (maximum degradation) is positively corelated with PSMA expression levels (PC3-PIP > LNCaP > 22Rv1), reaching approximately 75% in PC3-PIP cells. The degradation of EGFR induced by **Ctx-L3** and **Ctx-L5** was dose-dependent.

**Figure 4:**
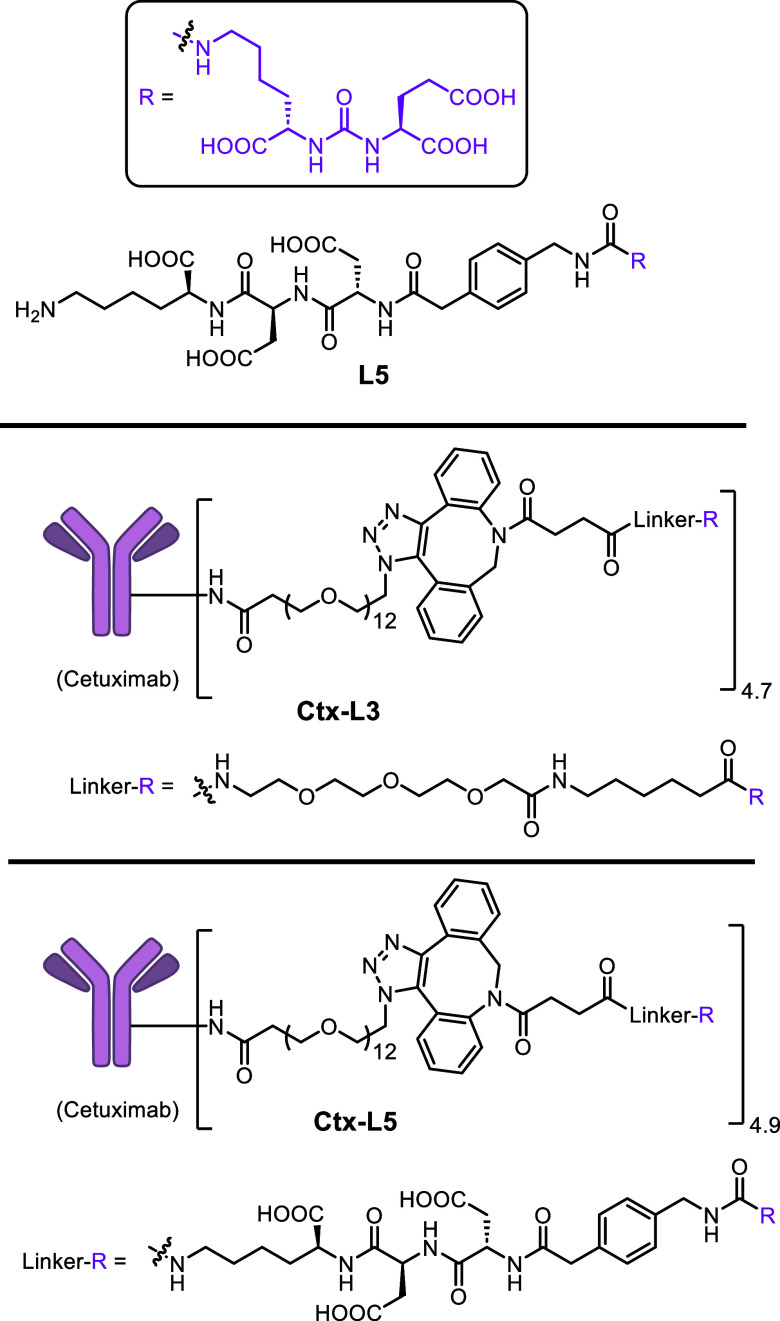
Structure of PSMA ligand **L5** and cetuximab-PSMA ligand conjugates with two different linkers. **L5** was used in the competitive study of EGFR degradation induced by PTACs.

**Figure 5:**
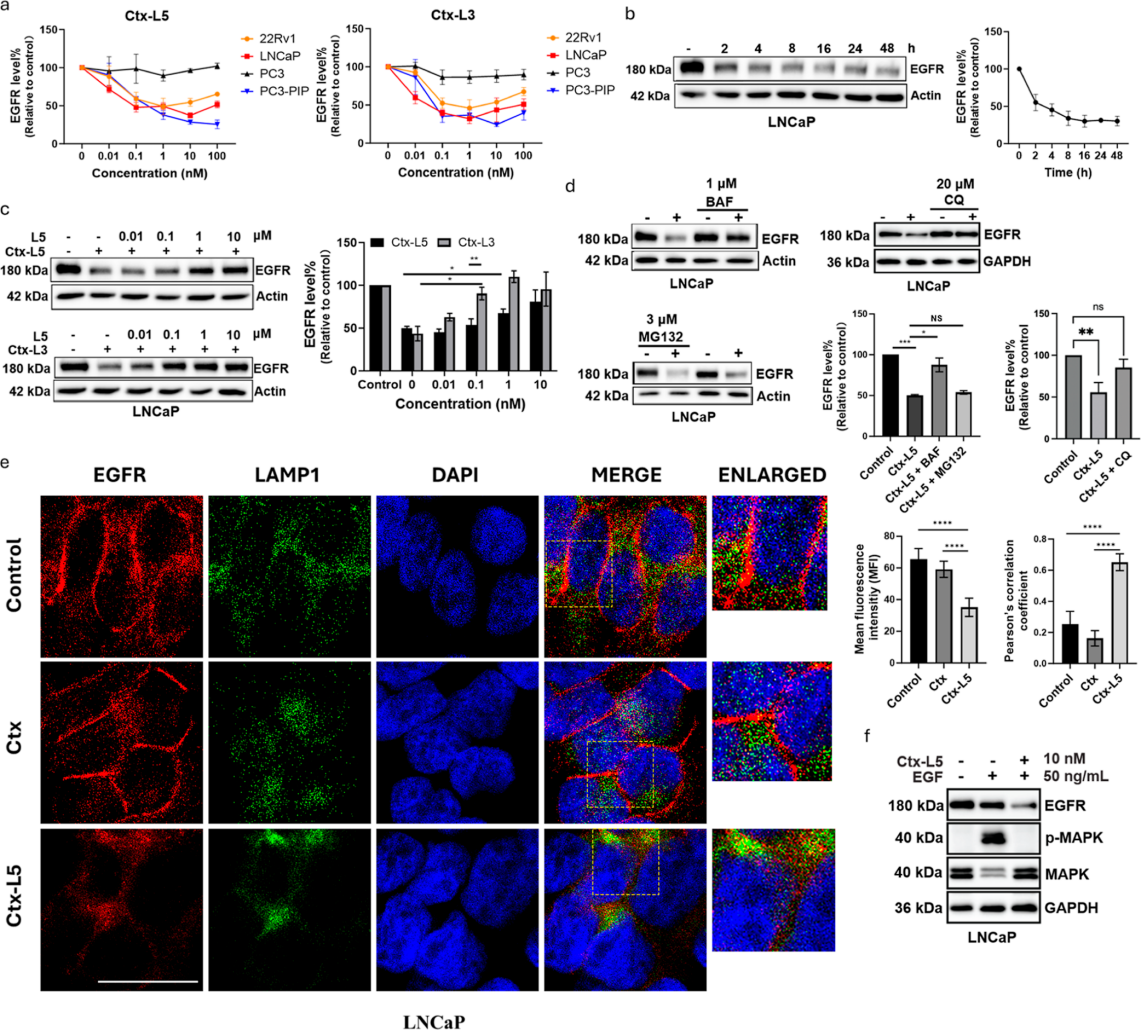
PTACs mediate the lysosomal degradation of EGFR by recruiting PSMA. (a) Dose–response of EGFR degradation after 24 h of treatment with **Ctx-L5** and **Ctx-L3** in four prostate cancer cell lines: PC3-PIP, LNCaP, 22Rv1, and PC3 (*n* = 3). (b) Time course of EGFR degradation induced by **Ctx-L5** (10 nM) in LNCaP cells (*n* = 3). (c) Inhibition of EGFR degradation induced by **Ctx-L3** (10 nM) and **Ctx-L5** (10 nM) in the presence of free ligand **L5** (0.01–10 μM) in LNCaP cells for 7 h (*n* = 3). (d) Inhibition of EGFR degradation induced by **Ctx-L5** (10 nM for BAF and MG132, or 1 nM for CQ) in the presence of Bafilomycin A1 (BAF, 1 μM), MG132 (3 μM), and CQ (20 μM) in LNCaP cells for 6 h (*n* = 3). (e) Immunofluorescent staining of EGFR degradation and lysosome colocalization after treatment of Ctx (10 nM) and **Ctx-L5** (10 nM) for 24 h in LNCaP cells. Scale bar: 25 μm. The intracellular fluorescence intensity is presented as mean fluorescence intensity (MFI) (*n* = 9). The colocalization was analyzed by Pearson’s correlation coefficients (*n* = 9). (f) Downregulation of EGFR, MAPK phosphorylation, and MAPK in LNCaP cells (*n* = 3). Data are presented as mean ± SD. The statistical significance was assessed using an unpaired two-tailed *t*-test, *P <0.05, **P <0.01, ***P <0.001, ****P <0.0001, NS: not significant.

Notably, the DC_50_ (concentration required for 50% degradation) for both **Ctx-L3** and **Ctx-L5** was below 100 pM in all three PSMA-positive cell lines, with the DC_50_ of **Ctx-L3** in LNCaP as low as 4.3 pM, which is one of the most potent DC_50_ values for LYTACs and related degraders reported to date. The DC_50_ and *D*_max_ values of **Ctx-L3** and **Ctx-L5** across PC3-PIP, LNCaP, and 22Rv1 cells are summarized in Table S1.

Despite the noticeable difference in uptake efficiency between **L3-biotin** and **L5-biotin**, **Ctx-L3** and **Ctx-L5** demonstrate a similar effective concentration range for degradation, with **Ctx-L3** even showing potency slightly better than that of **Ctx-L5**. These results indicated the degradation efficiency is not solely dependent on the binding affinity of the LTR ligand. This phenomenon may be attributed to the formation of relatively more unproductive binary complexes (degrader-PSMA) with a high-affinity PSMA ligand, compared to the productive ternary complexes required for effective degradation in these two scenarios.^55^

Our time course study indicated that **Ctx-L5** exerts a rapid and sustained effect on EGFR degradation in LNCaP cells at 10 nM. Approximately 50% of EGFR degradation was observed 2 h post-treatment and the maximal degradation persisted for at least 48 h (Figure 5b).

To further investigate the role of PSMA in degrader-induced EGFR degradation, **Ctx-L3** and **Ctx-L5** were cotreated with an increasing amount of free PSMA ligand **L5** in LNCaP cells. We observed that the degradation of EGFR induced by PTACs was abolished by **L5** in a dose-dependent manner. These results suggest that PSMA is involved in mediating EGFR degradation. Interestingly, degrader **Ctx-L5**, with a higher-affinity ligand, is much less sensitive to competition from **L5** (Figure 5c). The binding affinity of the PTACs was supported by heterologous competition binding assays using [^177^Lu]­Lu-PSMA-617 as the tracer ligand (Figure S7). The results suggested that PTACs with higher binding affinities have an enhanced ability to resist competition from endogenous ligands.

To investigate the mechanistic pathways of EGFR degradation, cells were treated with the lysosomal inhibitor Bafilomycin A1 (BAF), Chloroquine (CQ), and proteasome inhibitor MG132. Treatment with BAF or CQ significantly reduced EGFR degradation in the presence of **Ctx-L5**, while MG132 had no effect (Figure 5d). These results confirm that **Ctx-L5**-induced EGFR degradation occurs via the lysosomal pathway rather than the proteasomal pathway. In addition, pretreatment with known endocytosis inhibitors, including chlorpromazine, methyl-β-cyclodextrin, or the chaperone-mediated autophagy (CMA) regulator VER-155008, applied at concentrations as reported,^56^ did not appreciably affect **Ctx-L5**-induced EGFR degradation (Figure S8). While these data support PSMA-dependent internalization and subsequent lysosomal degradation, the precise endocytic pathways involved warrant future investigation.

Confocal imaging further validated the degradation effect of EGFR induced by **Ctx-L5** in the LNCaP cells. Degrader-treated cells showed a marked reduction of EGFR compared to control and Ctx-treated groups. Additionally, a distinct translocation of EGFR from the cell surface into the cytoplasm was observed after 24 h of **Ctx-L5** treatment. To track EGFR postinternalization, costaining of EGFR and lysosomal marker LAMP1 revealed that EGFR predominantly colocalized with lysosomes following degrader incubation (Figure 5e). These findings demonstrate that PTACs facilitate the delivery of membrane proteins to lysosomes for degradation.

To assess the downstream functional consequences, we pretreated LNCaP cells with **Ctx-L5** (10 nM, 6 h) followed by EGF stimulation (50 ng/mL, 30 min, Figure 5f). Compared with the control, EGF treatment alone robustly increased the *p*-MAPK level and concomitantly decreased total MAPK, while EGFR levels remained largely unchanged. In contrast, **Ctx-L5** treatment resulted in pronounced EGFR reduction and abolished the EGF-induced *p*-MAPK response, with total MAPK unchanged. These findings suggest that EGFR degradation triggered by **Ctx-L5** may attenuate EGF-induced signaling, consistent with an on-target PTAC mechanism.

### Prostate Cancer Selectivity of PTACs Targeting EGFR

To investigate the selectivity of **Ctx-L5** for EGFR degradation in PSMA-positive cancer cells, the potency of the degrader was compared across prostate cancer cells PC3-PIP, LNCaP, 22Rv1, and PC3, as well as two nonprostate cancer cell lines, MDA-MB-231 and Huh7. As expected, degradation was observed in the following PSMA-positive cancer cell lines: PC3-PIP, LNCaP, and 22Rv1. The total PSMA level was not affected by the degrader (Figure S9). In contrast, no degradation occurred in PSMA-negative cancer cell lines: PC3, MDA-MB-231, and Huh7 (Figure 6). These results indicate that PTACs can selectively degrade EGFR in PSMA-positive prostate cancer cell lines.

**Figure 6:**
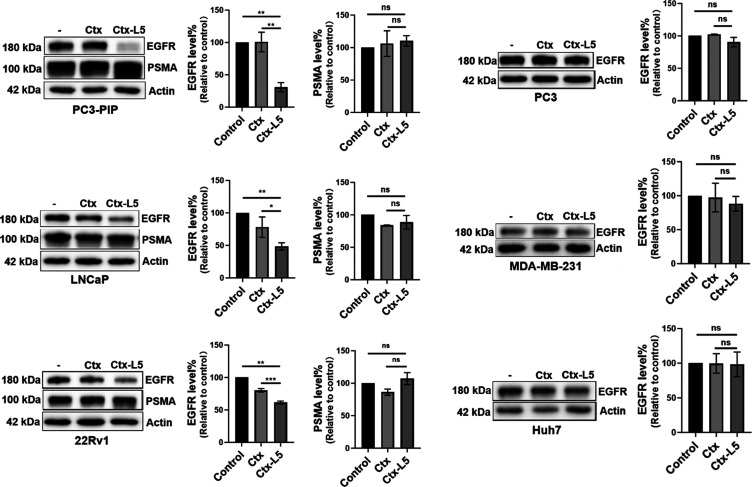
Selectivity of EGFR degradation (24 h) induced by Ctx (10 nM) and **Ctx-L5** (10 nM) in PSMA-positive and -negative cell lines, and PSMA levels following EGFR degradation in PSMA-positive cancer cell lines. PSMA-positive cancer cell lines: PC3-PIP, LNCaP, 22Rv1; PSMA-negative cancer cell lines: PC3, MDA-MB-231, and Huh7. Data are presented as mean ± SD. The statistical significance was assessed using an unpaired two-tailed *t*-test, **P <0.01, NS: not significant.

### Degradation of PD-L1 Mediated by PTACs

To demonstrate the versatility of the PTAC platform, we extended its application to PD-L1, a membrane protein critical for tumor immune evasion. Atezolizumab (Atz), an FDA-approved PD-L1 antibody, was conjugated with **L5** via copper-free click chemistry to generate **Atz-L5** (Figure 7a). **Atz-L5** induced potent PD-L1 degradation in PC3-PIP cells, with a DC_50_ of 18 pM and *D*_max_ of 70% at 10 nM for 24 h (Figure 7b). Notably, **Atz-L5** exhibited even more efficient PD-L1 degradation in LNCaP cells, with a DC_50_ of 2 pM and a *D*_max_ of 81% for 24 h (Figure 7c).

**Figure 7:**
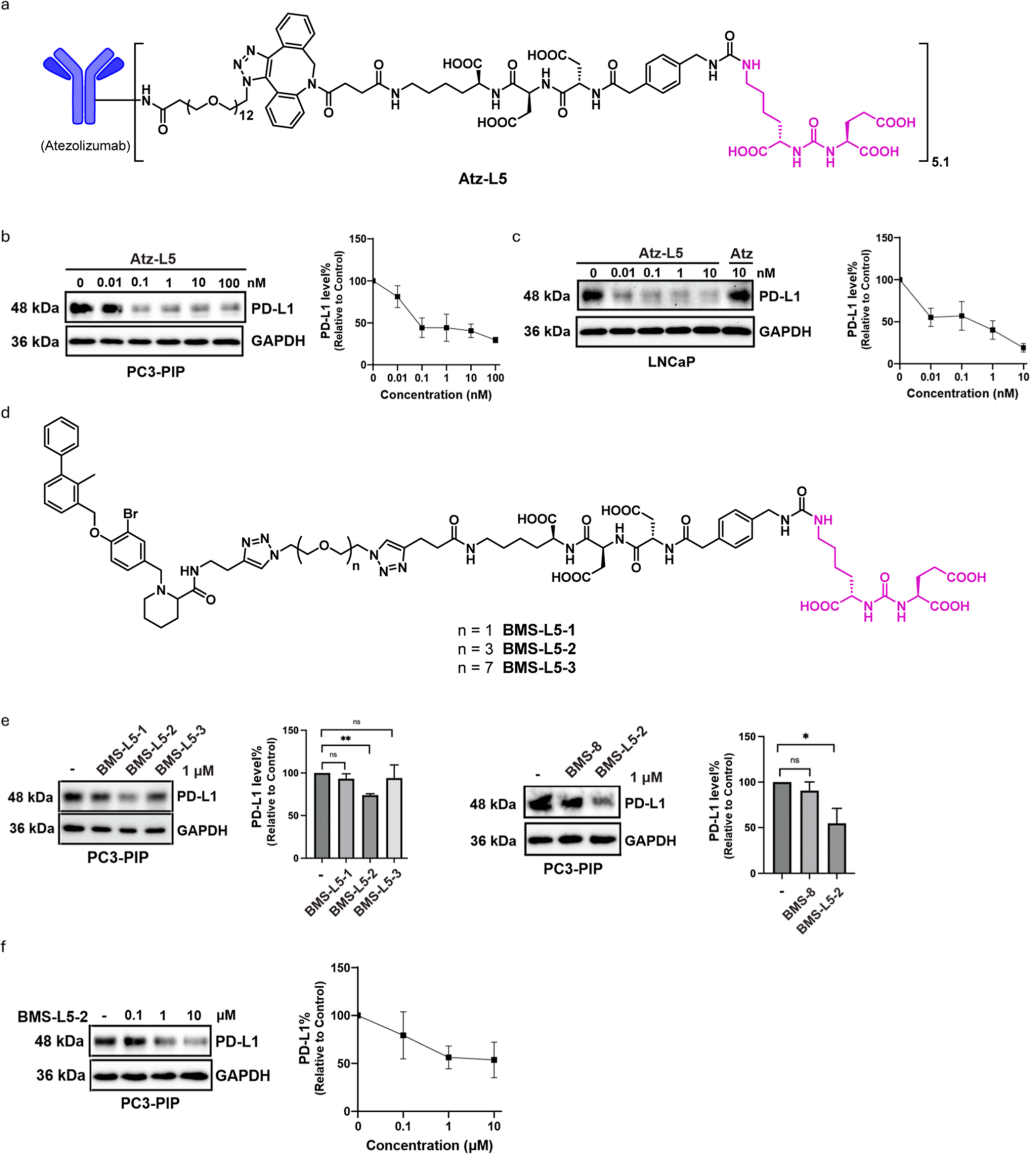
Degradation of PD-L1 mediate by both antibody- and small molecule-based PTACs. (a) Structure of the atezolizumab-L5 conjugate (**Atz-L5**). (b) Dose–response of PD-L1 degradation induced by **Atz-L5** with indicated concentration in PC3-PIP cells for 24 h, DC_50_ = 18 pM, *D*_max_ = 70% (*n* = 3). (c) Dose–response of PD-L1 degradation induced by **Atz-L5** with indicated concentration in LNCaP cells for 24 h, DC_50_ = 2 pM, *D*_max_ = 81% (*n* = 3). (d) The structures of **BMS-L5–1**, **BMS-L5–2**, and **BMS-L5–3**. (e) PD-L1 degradation induced by **BMS-L5–1**, **BMS-L5–2**, **BMS-L5–3**, and **BMS-8** at 1 μM in PC3-PIP cells for 24 h (*n* = 3). (f) Dose–response of PD-L1 degradation after 24 h of treatment with **BMS-L5–2** with indicated concentration in PC3-PIP cells for 24 h (*n* = 3). Data are presented as mean ± SD. The statistical significance was assessed using an unpaired two-tailed *t*-test, *P <0.05, **P <0.01, NS: not significant.

We further applied the PTAC strategy to small-molecule degraders by linking PD-L1 binder **BMS-8** with PSMA ligand **L5** through PEG linkers of different lengths, generating **BMS-L5–1**, **BMS-L5–2**, and **BMS-L5–3** (Figure 7d). Among them, **BMS-L5–2** showed the highest activity, achieving 47% degradation at 10 μM (Figure 7e and f).

These results confirm the versatility of the PTAC platform across both antibody conjugates and small-molecule ligands. Notably, while both antibody- and small-molecule-based PTACs enabled PD-L1 degradation, the small-molecule conjugates required substantially higher concentrations to achieve comparable effects, indicating that the intrinsic properties and compatibility of the POI ligand can markedly influence degradation efficiency. Given the role of PD-L1 in immune evasion, these findings suggest that PTAC-mediated PD-L1 degradation could potentially enhance immune recognition of prostate cancer cells, providing a therapeutic rationale for further exploration.

## Conclusions

In summary, this work successfully demonstrated that PSMA can function as an LTR, enabling applications in tissue-specific TPD. PTACs were shown to facilitate rapid and selective lysosomal degradation of both extracellular antibiotin model protein target and therapeutically relevant membrane proteins, such as EGFR and PD-L1, in PSMA-positive prostate cancer cells. Two PTACs for EGFR degradation, **Ctx-L3** and **Ctx-L5**, bearing ligands with different PSMA binding affinities, exhibited comparable degradation efficiencies despite their differing affinities. However, PTACs with higher binding affinities demonstrate superior resistance to competition from LTR ligands, indicating that they may be less susceptible to interference from endogenous PSMA substrates. This characteristic enhances their potential for therapeutic applications, where competition with naturally occurring PSMA substrates could otherwise diminish efficacy. Notably, the DC_50_ values for both PTACs are all below 100 pM, with one of them as low as 4.3 pM, among the lowest reported for LYTACs and related degraders to date. PTACs for PD-L1 degradation were demonstrated in both antibody- and small-molecule-based formats, enriching this versatile platform to an additional target and degrader modality. By utilizing PSMA as an LTR, this study extends the strategy of degradation of extracellular and membrane proteins and demonstrates the potential of PTACs as a tool for prostate cancer-selective degraders. These findings broaden the therapeutic landscape for TPD, paving the way for the development of innovative treatments for PSMA-positive cancers and other diseases.

## Supplementary Material



## Notes

Additional figures and table as mentioned in the text, experimental details, materials, methods, NMR spectra, and HPLC data (PDF)
