# Supplementary material for: Development of Prostate-Specific Lysosome-Targeting Degraders
Source: J Am Chem Soc. 2026 Feb 11;148(7):7171–80. doi: 10.1021/jacs.5c18594 (PMC12951434; doi:10.1021/jacs.5c18594)
Supplement: Supplementary file 1 [file ja5c18594_si_001.pdf]

Supporting Information

Development of Prostate-Specific Lysosome-Targeting Degraders

Deqin Cai[1, †] Xuankun Chen[1, †] Yaxian Zhou[1, †] Malick Bio Idrissou,[3] Reinier Hernandez,[3, 4] and Weiping Tang[1, 2, *]

1. Lachman Institute of Pharmaceutical Development, School of Pharmacy, University of Wisconsin-Madison, Madison, WI 53705, USA.

2. Department of Chemistry, University of Wisconsin-Madison, Madison, WI 53706, USA.

3. Department of Medical Physics, University of Wisconsin School of Medicine and Public Health, Madison, WI, 53705, United States.

4. Department of Radiology, University of Wisconsin School of Medicine and Public Health, Madison, WI, 53792, United States

[†] These authors contributed equally.

Table of Contents

1. Supporting Figures and Table S1 .... S3

2. Methods in Biology S9

Cell culture S9

Cellular uptake of anti-biotin-647 .... S9

Antibody labeling with N_3-PEG_{12}-C_3-OSu S9

Click chemistry of antibody-azide with DBCO-PSMA ligands S9

MALDI-MS S9

Targeted protein degradation S10

Western blotting and in-gel fluorescence analysis S10

Competition assay S10

Confocal microscopy S10

Heterologous competition binding S10

3. Methods in Chemistry S11

General information S11

Synthetic procedures for the preparation of L1-L5-biontin S13

Synthetic procedures for the preparation of L3-DBCO and L5-DBCO S21

Synthetic procedures for the preparation of BMS-L5-1, BMS-L5-2 and BMS-L5-3 .... S22

4. NMR..........S27

5. HPLC analysis..........S46

6. References..........S50

1. Supporting Figures and Table S1

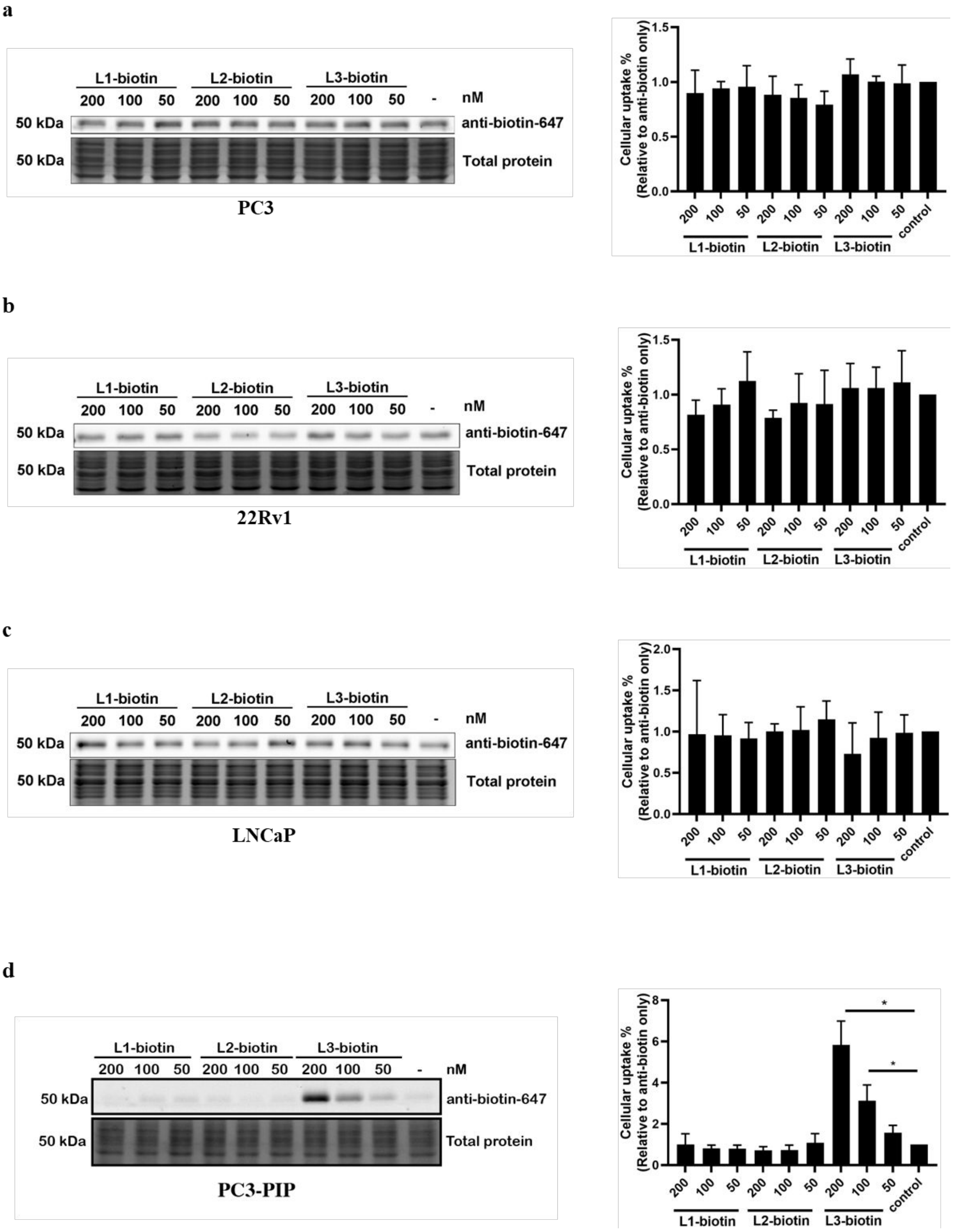

Figure S1. Uptake of anti-biotin-647 (50 nM) in PC3, 22Rv1, LNCaP, and PC3-PIP cells treated with L1-biotin, L2-biotin, and L3-biotin (50, 100, 200 nM) for 24h (n=3). Data are presented as mean ± SD. The statistical significance was assessed using an unpaired two-tailed t-test, *$P < 0.05$.

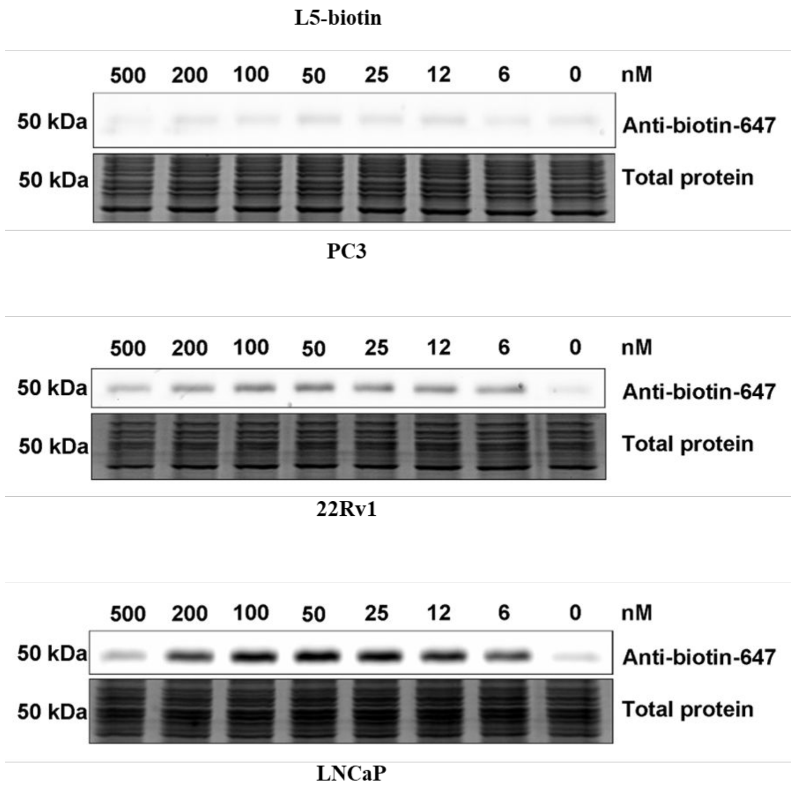

Figure S2. Dose-response of anti-biotin-647 uptake (24h) treated with L5-biotin in LNCaP, 22Rv1, or PC3 cells (n=3).

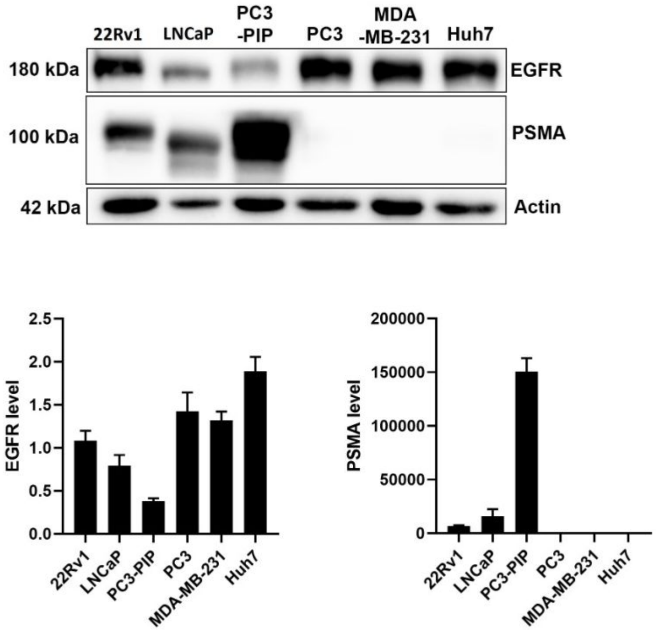

Figure S3. Endogenous expression level of PSMA and EGFR in cancer cells used in this study.

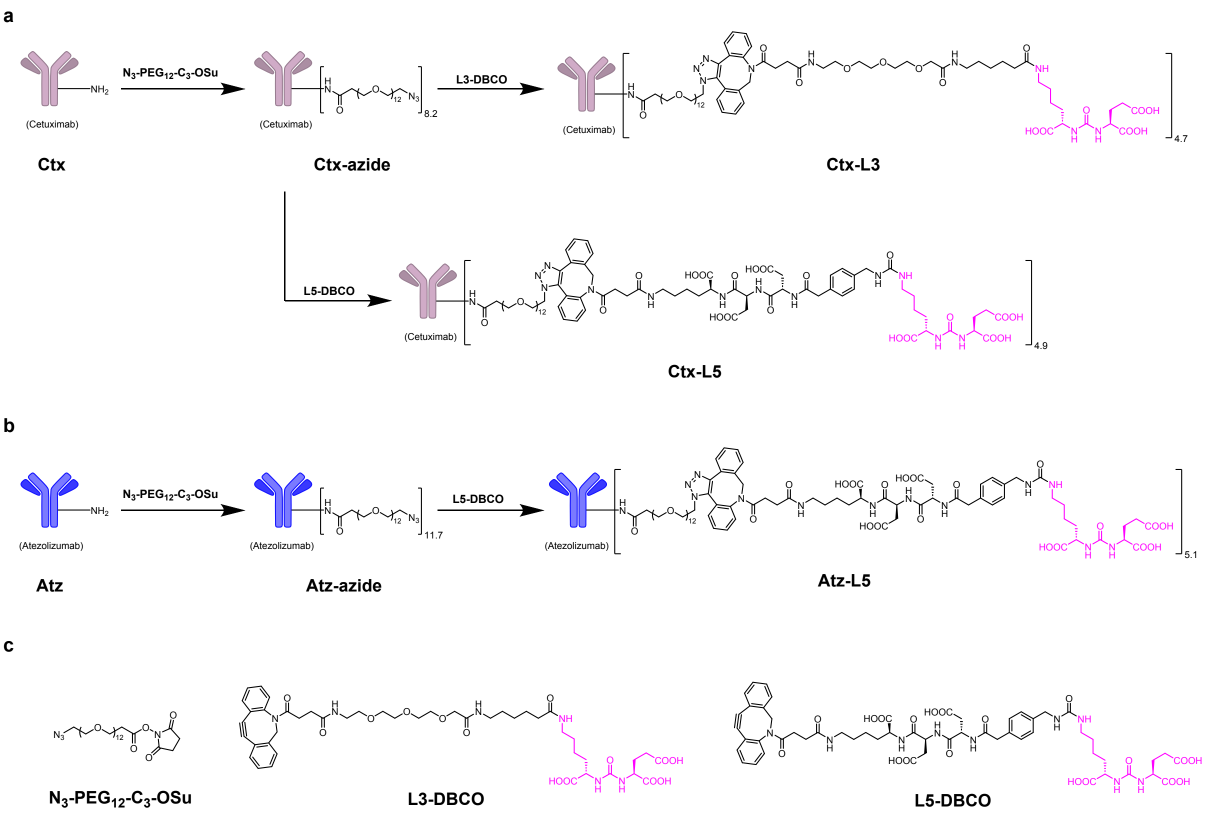

Figure S4. Preparation of PTACs Ctx-L3, Ctx-L5, and Atz-L5. a. synthesis of **Ctx-L3** and **Ctx-L5**. b. synthesis of **Atz-L5**. c. The key intermediates used in the preparation of PTACs.

Ctx

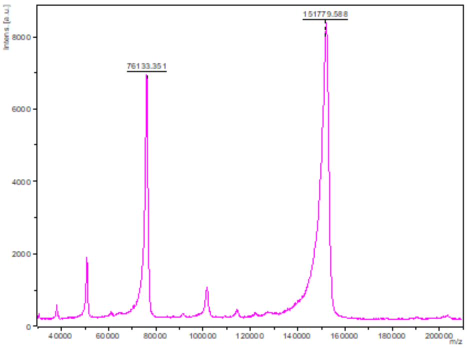

Ctx-azide

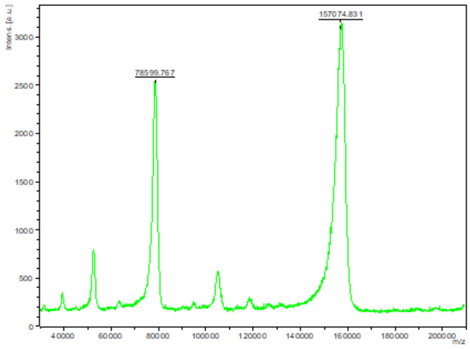

Ctx-L3

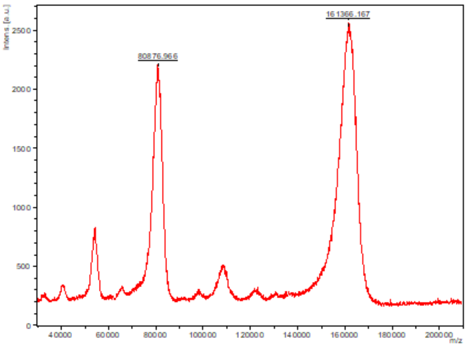

Ctx-L5

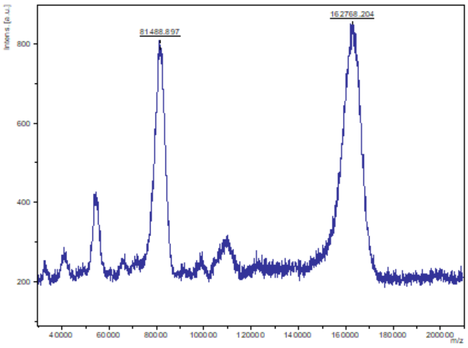

Atz

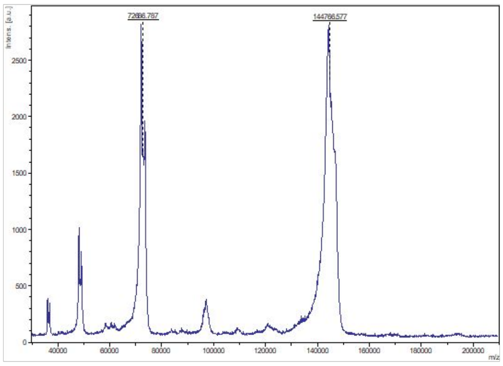

Atz-azide

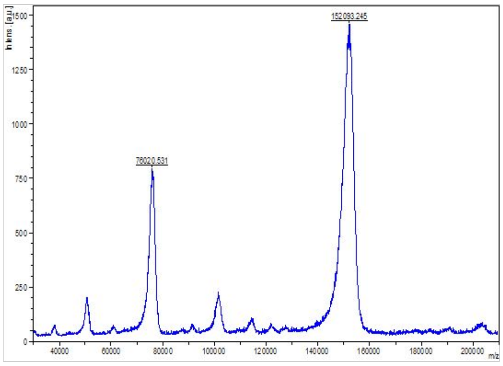

Atz-L5

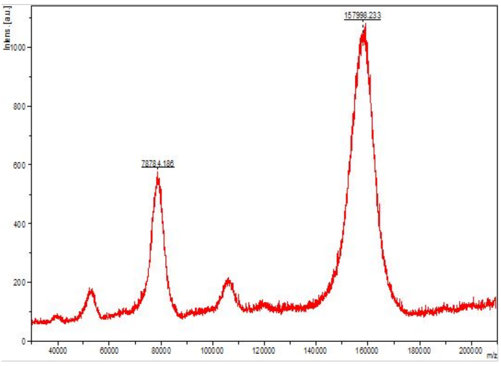

Figure S5. MALDI-TOF-MS characterization of Ctx-L3, Ctx-L5, and Atz-L5.

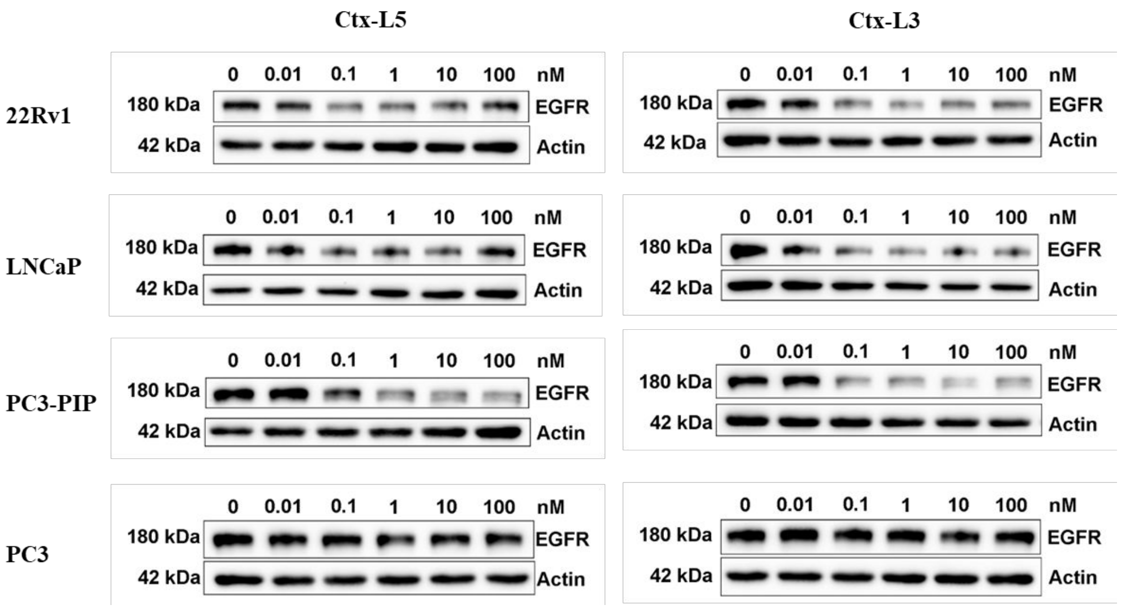

Figure S6. Dose-response of EGFR degradation after 24 hours of treatment with Ctx-L5 and Ctx-L3 in four prostate cancer cell lines: PC3-PIP, LNCaP, 22Rv1, and PC3. (n=3).

Table S1 DC_{50},24 h and D_{max} of Ctx-L5 and Ctx-L3 in prostate cancer cell lines

	**Ctx-L5**		**Ctx-L3**	
Cell line	DC_{50} (pM)	D_{max} (%)	DC_{50} (pM)	D_{max} (%)
22Rv1	19.3	50.9	19.7	53.9
LNCaP	9.0	62.7	4.3	68.0
PC3-PIP	80.5	74.5	22.4	75.7

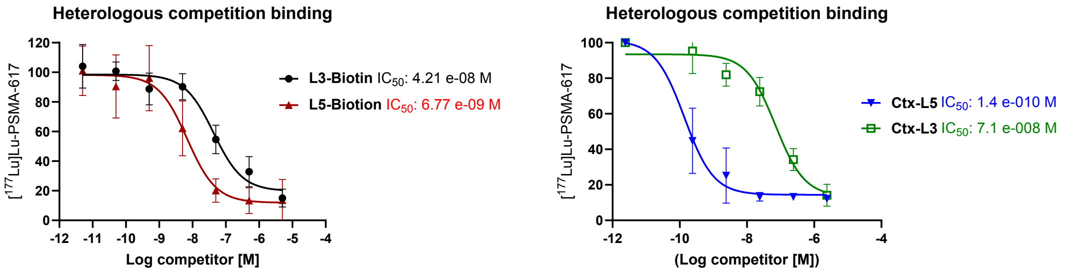

Figure S7. Heterologous competition binding assay of L3-Biotin and L5-Biotin (left), and their corresponding antibody conjugates Ctx-L3 and Ctx-L5 (right), against [^{177}Lu]Lu-PSMA-617 in PSMA-positive cells. The IC_{50} values were determined by nonlinear regression fitting. (n=3)

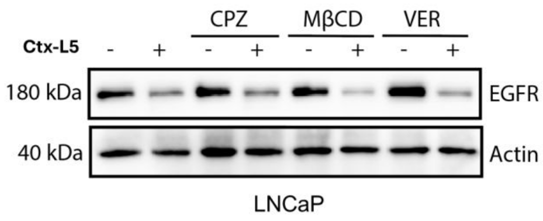

Figure S8. Effects of endocytosis inhibitors and CMA regulator on Ctx-L5-mediated EGFR degradation. LNCaP cells were treated with or without endocytosis inhibitors chlorpromazine (CPZ, 2 μg/mL), methyl-β-cyclodextrin (MβCD, 50 μM), and CMA-regulator VER-155008 (VER, 0.5 μM) for 4 h time before adding Ctx-L5 (10 nM) for additional 24 h.

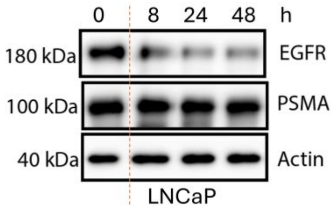

Figure S9. Monitoring PSMA levels in LNCaP cells over a 48 h period following treatment with Ctx-L5 (10 nM). Western blot analysis was performed for EGFR, PSMA, and Actin at 0, 8, 24, and 48 h.

2. Methods in Biology

Cell culture

PC3, 22Rv1, LNCaP and PC3-PIP cells were maintained in RPMI supplemented with 10% fetal bovine serum, 1% HEPES, 1% sodium pyruvate and 1% penicillin/streptomycin under 5% CO_2 at 37 °C. MDA-MB-231 cells were maintained in high-glucose DMEM supplemented with 10% fetal bovine serum, and 1% penicillin/streptomycin under 5 % CO_2 at 37 °C. Huh7 cells were cultured in low-glucose DMEM supplemented with 10% fetal bovine serum, 1% non-essential amino acids, 1% sodium pyruvate, 1% L-glutamine and 1% penicillin/streptomycin under 5 % CO_2 at 37 °C.

Cellular uptake of anti-biotin-647

Cells were seeded at 70% confluence and maintained in 200 μL complete culture media in a 48-well plate. The next day, cells were treated sequentially with 25 μL medium containing anti-biotin-647 and 25 μL medium containing PBS, antibody, or degrader at different concentrations. The cells were incubated at 37 °C for different time periods and then washed twice with PBS before being harvested for in-gel fluorescence analysis.

Antibody labeling with N_3-PEG_{12}-C_3-OSu

To label the antibody with PSMA ligand, 100 μL of the antibody (concentration at 1.8 mg/mL) in PBS was mixed with N_3-PEG_{12}-C_3-OSu at 1:50 molar ratio. The reaction was incubated overnight at room temperature on a rotator, followed by filtration with 500 μL of PBS 5 times using a 10 kDa Amicon Centrifugal Filter. The mass of unmodified antibody and N_3-labeled antibody was analyzed by MALDI-MS, and the average number of N_3 per antibody was calculated based on the increase in molecular weight divided by the molecular weight increase by a single linker-N_3 (625). The concentration of PSMA ligands-labeled antibody was determined by BCA assay.

Click chemistry of antibody-azide with DBCO-PSMA ligands

To label the antibody-azide with DBCO-PSMA ligands (**L3-DBCO** or **L5-DBCO**), 200 μL of the antibody-azide (concentration at 1.8 mg/mL) in PBS was mixed with DBCO-PSMA ligands ester at a 1:50 molar ratio. The reaction was incubated overnight at room temperature on a rotator, followed by filtration with 500 μL of PBS 5 times using a 10 kDa Amicon Centrifugal Filter. Then, the concentration of PSMA ligands-labeled antibody was determined by BCA assay. The mass of PSMA ligands-labeled antibody was analyzed by MALDI-MS, and the average number of PSMA ligands per antibody was calculated based on the increase in molecular weight divided by the molecular weight increase by a single DBCO-PSMA ligand (**L3-DBCO**: 909 and **L5-DBCO**: 1156).

MALDI-MS

Samples were characterized by the following method: Matrix solution was made by dissolving α-Cyano-4-hydroxycinnamic acid (CHCA) in 50% Acetonitrile/H_2O at a final concentration of 10 mg/mL. The sample was absorbed on Omix C4 pipette tips, followed by washing with 0.1% TFA three times and then eluted with 20 μL 75% Acetonitrile/H_2O. 1 μL sample solution and 1 μL CHCA solution were spotted on the MALDI target plate and mixed thoroughly before the spot was allowed to dry at room temperature. MALDI-MS spectra were acquired on the Bruker UltraFlex MALDI-TOF/TOF mass spectrometer operated in linear positive ion mode and plots were generated by Bruker flexAnalysis 4.2.

Targeted protein degradation

Cells were seeded at 70% confluence in a 24-well plate one day before treatment. Then, cells were treated with PTACs targeting EGFR and PD-L1 at various concentrations for indicated time periods before collection for western blot analysis. PC3, LNCaP, PC3-PIP, and Huh7 cells were pre-incubated with 100 ng/mL human IFNγ for 16 h to induce PD-L1 expression before degrader treatment.

Western blotting and in-gel fluorescence analysis

Cells were lysed in 1x RIPA lysis buffer (25 mM Tris, pH 7–8, 150 mM NaCl, 0.1% (w/v) sodium dodecyl sulfate (SDS), 0.5% sodium deoxycholate, 1% (v/v) Triton X-100, protease inhibitor cocktail (Roche, one tablet per 10 mL)) on ice for 10 min, followed by centrifugation at 16,000 × *g* at 4 °C for 15 min. The supernatant was collected and adjusted to equal amounts after determining the protein concentration using the BCA assay. Lysates were then mixed with the 4x Laemmli Loading Dye and heated at 99 °C for 5 min before being loaded onto 7.5% or 12% SDS−polyacrylamide gel electrophoresis. For western blotting, the gel was transferred to a PVDF membrane, blocked in 5% (w/v) nonfat milk in the TBST washing buffer (137 mM NaCl, 20 mM Tris, 0.1% (v/v) Tween) and then incubated with primary antibodies at 4 °C overnight. After 3 washes with TBST, the membrane was incubated with secondary HRP-linked antibodies for 1 h, and then washed 3 times with TBST. Then the membrane was incubated in the Clarity ECL substrate for 3–5 min before acquiring the immunoblot by ChemiDoc MP Imaging Systems. For in-gel fluorescence analysis, the fluorescence images of the gel were directly acquired by ChemiDoc MP Imaging Systems and the total protein was detected using Coomassie blue staining. Western blot and in-gel fluorescence images were acquired by Image Lab Touch Software (v 2.0.1.35). Western blot bands intensity was analyzed using ImageJ (v 1.53a).

Competition assay

LNCaP cells were plated and co-treated with **L5** (0-10 μM) and 10 nM of **Ctx-L3** and **Ctx-L5** in the same manner as mentioned above. After 7 h, cells were washed twice with PBS, and the degradation of EGFR was analyzed as mentioned above.

Confocal microscopy

LNCaP cells were seeded onto 8-well chamber slides at a density of 20,000 cells per well in 200 μL of complete culture medium. After adhesion, cells were treated with 10 nM of Ctx-L5 and Ctx for 24 h at 37 °C. Cells were then washed with PBS for 3 times and fixed with 4% paraformaldehyde for 15 min followed by permeabilization with 0.5% Triton X-100 for 5 min. After blocking with 5% BSA for 1 h at room temperature, the cells were co-incubated with rabbit anti-EGFR and mouse anti-LAMP1 antibody in 1% BSA overnight at 4 °C. The next day, cells were washed with PBS and then incubated with anti-mouse-488 and anti-rabbit-594 secondary antibodies for 1 h at room temperature. Then the cells were mounted with slowfade-antifade mounting medium containing DAPI after three washes. Images were acquired by Leica SP8 3X STED super-resolution microscope at 60x magnification with a 10x eyepiece and analyzed by ImageJ. The Pearson's correlation coefficients and mean fluorescence intensity (MFI) were analyzed by Leica LAS-X software (v 2.6).

Heterologous competition binding

[^{177}Lu]Lu-PSMA-617 radiolabeling

Radiolabeling of PSMA-617 with ^{177}Lu was performed by adding 2 mCi (74 MBq) of the radiometal to 10

μg of PSMA-617 in NaOAc buffer (0.5 M, pH 5.5). The reaction mixture was then incubated at 95°C for 30 minutes with constant shaking at 500 rpm. [^{177}Lu]Lu-PSMA-617 was purified using an HLB solid phase extraction (Waters, USA) cartridge. The purified tracer was eluted in absolute ethanol, dried in a stream of nitrogen, and reconstituted in normal saline containing 0.1% Tween 20 (excipient).

Radiolabeling yield was assessed by instant thin-layer chromatography (iTLC) using silica-impregnated paper (PerkinElmer, USA) as the stationary phase and 50 mM EDTA as the mobile phase. Free radiometals moved with the solvent front (Rf = 1), while [^{177}Lu]Lu-PSMA-617 remained at the origin (Rf = 0).

Binding assay

For competitive binding assays, a constant concentration of [^{177}Lu]Lu-PSMA-617 (1 nM) radioligand was added to 5x10^3 PC3-PIP cells in a 0.22 μm hydrophilic, low-protein-binding GV clear 96-well plate (Fischer Scientific, USA) in the presence of increasing concentrations of cold **Ctx-L3** and **Ctx-L5** (0 (control) , 240 pM, 2.4 nM, 24 nM, 240 nM, 2.4 μM). After one hour incubation at 4°C, the plate was filtered, and unbound ligands were washed away. The remaining bound radioligand is measured using a gamma counter. Cell-associated radioactivity was plotted against increasing concentrations of **Ctx-L3** and **Ctx-L5**, and the PSMA binding affinity was determined as the 50% inhibition concentration (IC_{50}).

3. Methods in Chemistry

General information

Unless otherwise stated, all commercial reagents were used as received. Unless stated otherwise, reactions were performed at room temperature (rt). Thin-layer chromatography (TLC) was conducted on plates (EMD Chemical Inc. 60, F254). Flash column chromatography was performed with silica gel (Silicycle, 40-63 μm). ^{1}H and ^{13}C nuclear magnetic resonance spectra (NMR) were obtained on a Brooker 400 MHz and 500MHz. Chemical shifts were reported in parts per million (ppm), Coupling constants (J) were reported in Hertz (Hz). All high resolution mass spectra were performed by Analytical Instrument Center at the School of Pharmacy (UW-Madison) on an Electron Spray Injection (ESI) mass spectrometer. HPLC method A (standard method): The HPLC spectrometry analysis was processed on a Schimadzu CMB-40 system using a Schimadzu Nexcol C18 column (5 cm × 3.0 mm, 5 μm) for chromatographic separation. The mobile phases were 0.1% HCOOH in purified water (A) and 0.1% HCOOH MeCN (B). The gradient was increased from 5% to 100% in 10 min, then held at isocratic 100% B for 5 min, and then immediately stepped back down to 5% for 5 min re-equilibration. The flow rate was set at 1.0 mL/min. The column temperature was set at 30 °C. HPLC method B (for **L3-DBCO** and **L5-DBCO**) was similar to method A, except that the mobile phase was replaced with 10 mM NH_4HCO_3 in purified water (A) and MeCN (B).

General method for amide bond synthesis: HATU (1.5 e.q.) was added to a mixture of the amine (1.0 e.q.), the carboxylic acid (1.3 e.q.) and DIEA (2.0 e.q.) in DMF (0.1 M). The reaction was stirred at RT until LCMS indicated the end. The result mixture was purified by flash column to afford amide product.

General method for deprotection of t-butyl ester: The to be deprotected *t*-butyl ester was dissolved in DCM/TFA (2/1, 100 V). When LCMS indicated the end, the result mixture was concentrated and co-distilled with DCM twice. The result residue was purified by preparative HPLC C18 reverse column eluting with acetonitrile/water (0.1% formic acid). Note: MeOH should not be used when submitting samples to LCMS, a potential methyl ester will generate.

General method for click chemistry: The azide structure (1.0 e.q.) and the alkyne structure (1.0 e.q.) were

dissolved in DMF/water (10/1, 100 V to the azide structure). Copper(II) sulfate (0.5 e.q.) and sodium ascorbate (1.0 e.q.) were then added. The reaction was then degassed with argon and stirred at RT. More sodium ascorbate may be added until the end. The reaction mixture was purified by preparative HPLC C18 reverse column eluting with acetonitrile/water (0.1% formic acid) and then G-10 column to afford pure compounds. Note: The alkyne structure is difficult to remove from the product using a C18 column, but it can be effectively separated with a G-10 column due to differences in molecular size.

Synthetic procedures for the preparation of L1-L5-biontin

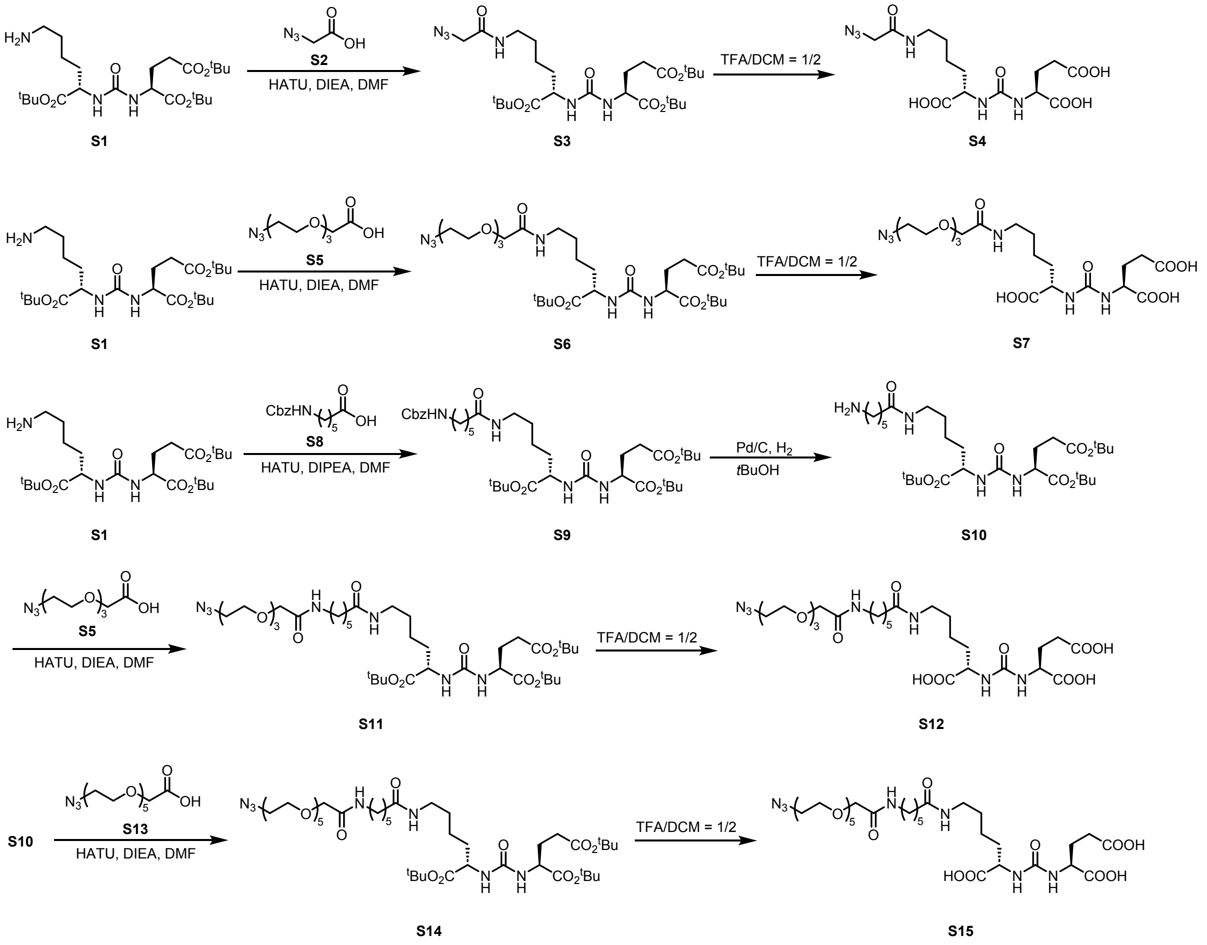

(((*S*)-5-(2-azidoacetamido)-1-carboxypentyl)carbamoyl)-*L*-glutamic acid (S4)

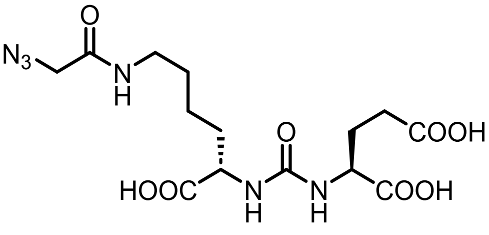

S4

S4 (32.8 mg, yield 29.8% in two steps) was obtained from **S1**[1] (100 mg, 0.21 mmol) according to the "*General method for amide bond synthesis*" and "*General method for deprotection of t-butyl ester*".

^{1}H NMR (400 MHz, D_2O) δ 4.17 (dd, *J* = 9.1, 5.1 Hz, 1H), 4.10 (dd, *J* = 8.8, 5.0 Hz, 1H), 3.91 (s, 2H), 3.15 (t, *J* = 6.8 Hz, 2H), 2.42 (t, *J* = 7.3 Hz, 2H), 2.15-2.03 (m, 1H), 1.94-1.82 (m, 1H), 1.81-1.69 (m, 1H), 1.69-1.56 (m, 1H), 1.54 – 1.40 (m, 2H), 1.40 – 1.23 (m, 2H). ^{13}C NMR (100 MHz, D_2O) δ 177.3, 177.2, 176.3, 170.1, 159.3, 53.2, 52.6, 51.9, 39.0, 30.6, 30.1, 27.7, 26.3, 22.2. LC-MS m/z = 403.1 $[M+1]^+$.

(17*S*,21*S*)-1-azido-11,19-dioxo-3,6,9-trioxa-12,18,20-triazatricosane-17,21,23-tricarboxylic acid (S7)

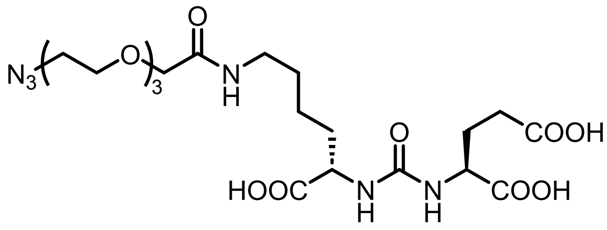

S7

S7 (12.2 mg, yield 19.8% in two steps) was obtained from **S1** (50 mg, 0.10 mmol) according to the "*General method for amide bond synthesis*" and "*General method for deprotection of t-butyl ester*".

^{1}H NMR (400 MHz, D_2O) δ 4.17 (dd, J = 9.0, 5.1 Hz, 1H), 4.10 (dd, J = 8.7, 5.0 Hz, 1H), 3.99 (s, 2H), 3.71 – 3.60 (m, 10H), 3.42 (dd, J = 5.6, 4.2 Hz, 2H), 3.19 (t, J = 6.8 Hz, 2H), 2.43 (t, J = 7.3 Hz, 2H), 2.16-2.03 (m, 1H), 1.95-1.83 (m, 1H), 1.83 – 1.71 (m, 1H), 1.70-1.58 (m, 1H), 1.55 – 1.43 (m, 2H), 1.41-1.26 (m, 2H). ^{13}C NMR (100 MHz, D_2O) δ 177.4, 177.4, 176.6, 172.4, 159.3, 70.3, 69.6, 69.5, 69.4, 69.2, 53.4, 52.8, 50.1, 38.6, 30.7, 30.1, 27.9, 26.5, 22.3. LC-MS m/z = 535.2 $[M+1]^+$.

Tri-tert-butyl (16*S*,20*S*)-3,10,18-trioxo-1-phenyl-2-oxa-4,11,17,19-tetraazadocosane-16,20,22-tricarboxylate (S9)

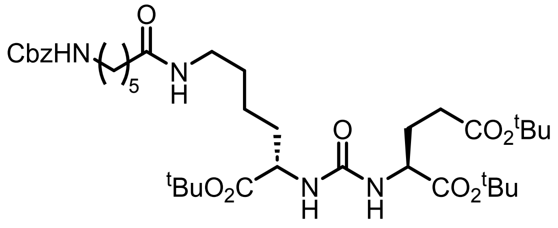

S9

S9 (0.52, yield 57.5%) was obtained from **S1** (600 mg, 1.23 mmol) according to the "*General method for amide bond synthesis*".

^{1}H NMR (400 MHz, $CDCl_3$) δ 7.40 – 7.26 (m, 5H), 6.52 (d, J = 5.7 Hz, 1H), 5.75 (d, J = 8.3 Hz, 1H), 5.61 (d, J = 7.9 Hz, 1H), 5.25-4.97 (m, 3H), 4.41-4.18 (m, 2H), 3.33-3.22 (m, 1H), 3.22-3.07 (m, 3H), 2.39-2.22 (m, 2H), 2.23 – 2.11 (m, 2H), 2.11-2.00 (m, 1H), 1.88 – 1.69 (m, 2H), 1.69 – 1.23 (m, 40H). ^{13}C NMR (100 MHz, $CDCl_3$) δ 173.4, 173.2, 172.5, 172.2, 157.5, 156.6, 136.7, 128.4, 128.0, 82.1, 81.4, 80.5, 66.5, 53.4, 52.9, 40.9, 39.0, 36.2, 32.4, 31.6, 29.5, 28.8, 28.0, 26.3, 25.3, 22.7. LC-MS m/z = 735.4 $[M+1]^+$.

Di-tert-butyl (((*S*)-6-(6-aminohexanamido)-1-(tert-butoxy)-1-oxohexan-2-yl)carbamoyl)-*L*-glutamate (S10)

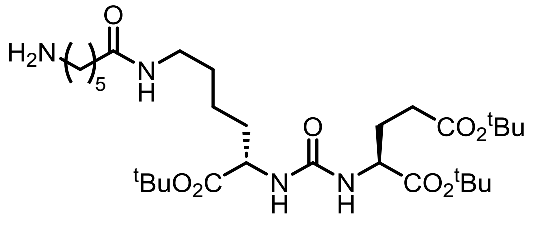

S10

Pd/C (10%, 30 mg) was added to a mixture of **S9** (300 mg, 0.41 mmol) in *t*-BuOH (4.5 mL) under nitrogen flow. The reaction flask was degassed and charged with a hydrogen balloon. The reaction was stirred at 30 ℃ overnight. Filtered and concentrated, co-distilled with acetonitrile and dried in vacuum to afford **S10** (224 mg, 91.4%) as a white foam solid. Note: The result product is an acidified form.

^{1}H NMR (400 MHz, $CDCl_3$) δ 7.26 (t, *J* = 5.7 Hz, 1H), 6.30-6.04 (m, 2H), 4.28 (td, *J* = 8.3, 5.1 Hz, 1H), 4.20 (td, *J* = 7.6, 4.6 Hz, 1H), 3.28-3.09 (m, 2H), 3.01 (t, *J* = 7.2 Hz, 2H), 2.42 – 2.14 (m, 4H), 2.10-1.99 (m, 1H), 1.90-1.74 (m, 3H), 1.75 – 1.55 (m, 5H), 1.55 – 1.27 (m, 35H). ^{13}C NMR (100 MHz, $CDCl_3$) δ 171.9, 171.2, 171.1, 170.6, 155.8, 80.1, 79.6, 78.7, 51.7, 51.0, 37.9, 37.1, 33.8, 30.0, 29.9, 26.9, 26.3, 26.2, 26.1, 24.9, 23.7, 23.0, 20.7. LC-MS m/z = 601.4 $[M+1]^+$.

(24*S*,28*S*)-1-azido-11,18,26-trioxo-3,6,9-trioxa-12,19,25,27-tetraazatriacontane-24,28,30-tricarboxylic acid (S12)

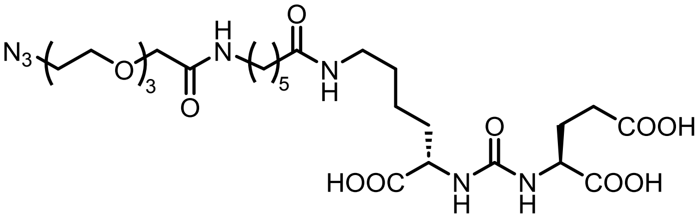

S12

S12 (33.1 mg, yield 30.7% in two steps) was obtained from **S10** (100 mg, 0.17 mmol) according to the "*General method for amide bond synthesis*" and "*General method for deprotection of t-butyl ester*".

^{1}H NMR (400 MHz, D_2O) δ 4.18 (dd, *J* = 9.0, 5.1 Hz, 1H), 4.10 (dd, *J* = 8.7, 5.0 Hz, 1H), 3.98 (s, 2H), 3.73 – 3.59 (m, 10H), 3.47 – 3.37 (m, 2H), 3.16 (t, *J* = 7.0 Hz, 2H), 3.10 (t, *J* = 6.7 Hz, 2H), 2.43 (t, *J* = 7.3 Hz, 2H), 2.20 – 2.04 (m, 3H), 1.95-1.82 (m, 1H), 1.82 – 1.70 (m, 1H), 1.69 – 1.57 (m, 1H), 1.57 – 1.39 (m, 6H), 1.39 – 1.15 (m, 4H). ^{13}C NMR (100 MHz, D_2O) δ 177.3, 177.2, 176.7, 176.4, 172.2, 159.2, 70.3, 69.6, 69.5, 69.2, 53.3, 52.7, 50.1, 38.9, 38.8, 35.7, 30.7, 30.1, 28.1, 27.8, 26.4, 25.5, 25.1, 22.3. LC-MS m/z = 648.3 $[M+1]^+$.

(30*S*,34*S*)-1-azido-17,24,32-trioxo-3,6,9,12,15-pentaoxa-18,25,31,33-tetraazahexatriacontane-30,34,36-tricarboxylic acid (S15)

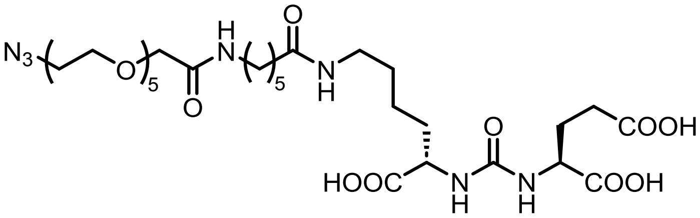

S15

S15 (16.4 mg, yield 30.1% in two steps) was obtained from **S10** (44.4 mg, 0.074 mmol) according to the "*General method for amide bond synthesis*" and "*General method for deprotection of t-butyl ester*".

^{1}H NMR (400 MHz, D_2O) δ 4.18 (dd, *J* = 9.0, 5.1 Hz, 1H), 4.10 (dd, *J* = 8.7, 5.0 Hz, 1H), 3.98 (s, 2H), 3.78 – 3.56 (m, 18H), 3.42 (dd, *J* = 5.5, 4.2 Hz, 2H), 3.17 (t, *J* = 7.0 Hz, 2H), 3.10 (t, *J* = 6.7 Hz, 2H), 2.43 (t, *J* = 7.3 Hz, 2H), 2.15 (t, *J* = 7.3 Hz, 2H), 2.13 – 2.04 (m, 1H), 1.95 – 1.83 (m, 1H), 1.82 – 1.71 (m, 1H), 1.69 – 1.59 (m, 1H), 1.59 – 1.40 (m, 6H), 1.39 – 1.28 (m, 2H), 1.28 – 1.18 (m, 2H). ^{13}C NMR (100 MHz, D_2O) δ 177.3, 177.3,

176.7, 176.5, 172.2, 159.3, 70.3, 69.6, 69.6, 69.6, 69.6, 69.5, 69.5, 69.2, 53.3, 52.7, 50.1, 38.9, 38.8, 35.7, 30.7, 30.1, 28.1, 27.8, 26.4, 25.5, 25.1, 22.3. LC-MS m/z = 736.3 $[M+1]^+$.

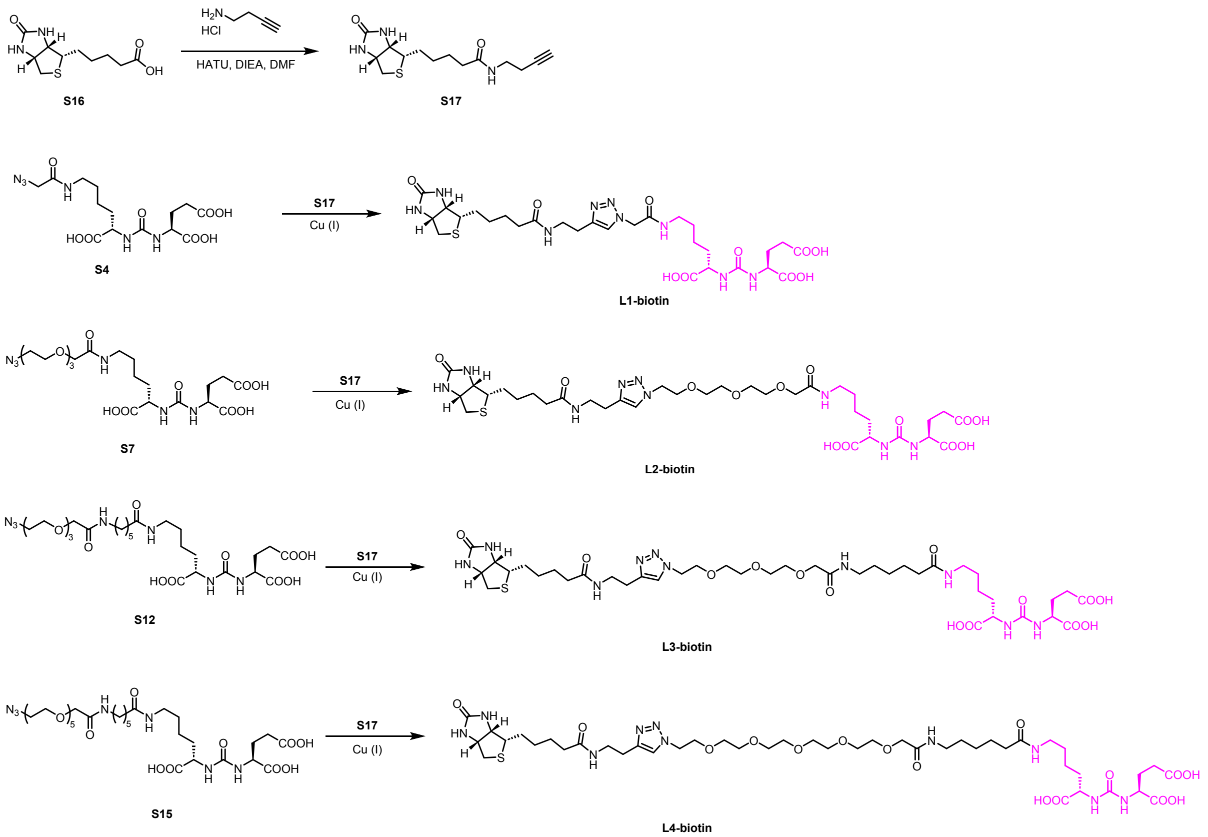

_N_-(but-3-yn-1-yl)-5-((3a*S*,4*S*,6a*R*)-2-oxohexahydro-1*H*-thieno[3,4-*d*]imidazol-4-yl)pentanamide (S17)

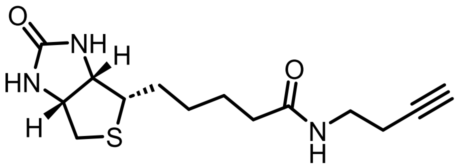

S17

HATU (570 mg, 1.5 mmol) was added to a mixture of 3-butyn-1-amine, hydrochloride (105 mg, 1 mmol, 1.0 e.q.), biotin **S16** (244 mg, 1 mmol) and DIEA (0.38 mL, 2.2 mmol) in DMF (3 mL). The reaction was stirred at RT until LCMS indicated the end. The result mixture was purified by preparative HPLC C18 reverse column eluting with acetonitrile/water (0.1% formic acid) to afford **S17** (70 mg, yield 23.7%).
^{1}H NMR (400 MHz, DMSO-d_6) δ 7.96 (t, J = 5.8 Hz, 1H), 6.41 (s, 1H), 6.35 (s, 1H), 4.36 – 4.27 (m, 1H), 4.13 (ddd, J = 7.7, 4.5, 1.9 Hz, 1H), 3.14 (tt, J = 11.6, 5.5 Hz, 4H), 2.86 – 2.78 (m, 2H), 2.62-2.55 (m, 1H), 2.27 (td, J = 7.1, 2.7 Hz, 2H), 2.06 (t, J = 7.4 Hz, 2H), 1.68 – 1.56 (m, 1H), 1.56 – 1.39 (m, 3H), 1.38 – 1.21 (m, 2H). LC-MS m/z = 296.2 $[M+1]^+$.

(((*S*)-1-carboxy-5-(2-(4-(2-(5-((3a*S*,4*S*,6a*R*)-2-oxohexahydro-1*H*-thieno[3,4-*d*]imidazol-4-yl)pentanamido)ethyl)-1*H*-1,2,3-triazol-1-yl)acetamido)pentyl)carbamoyl)-*L*-glutamic acid (L1-biotin)

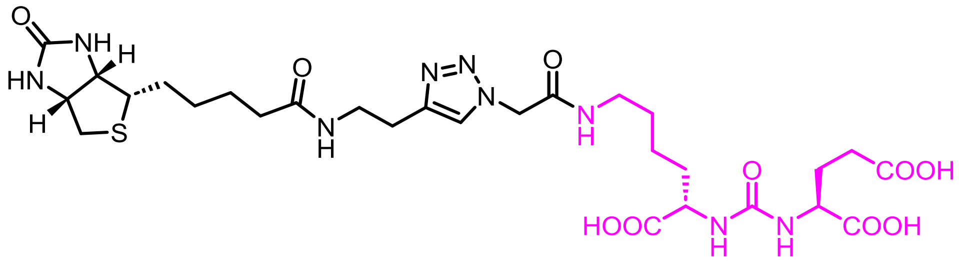

L1-biotin

L1-biotin (2.7 mg, yield 31.0%) was obtained from **S4** (5 mg, 0.012 mmol) according to the "*General method for click chemistry*".

^{1}H NMR (400 MHz, D_2O) δ 7.79 (s, 1H), 5.13 (s, 2H), 4.53 (dd, *J* = 8.0, 4.8 Hz, 1H), 4.32 (dd, *J* = 8.0, 4.4 Hz, 1H), 3.97-3.87 (m, 2H), 3.44 (t, *J* = 6.5 Hz, 2H), 3.27 – 3.13 (m, 3H), 2.96 – 2.83 (m, 3H), 2.71 (d, *J* = 13.1 Hz, 1H), 2.65 (s, 1H), 2.22 – 2.07 (m, 4H), 1.98 – 1.86 (m, 1H), 1.83 – 1.39 (m, 10H), 1.32-1.15 (m, 4H). ^{13}C NMR (100 MHz, D_2O) δ 182.6, 176.8, 167.6, 159.2, 145.4, 125.0, 62.0, 60.2, 55.3, 52.0, 39.7, 39.5, 38.6, 35.4, 34.2, 32.1, 29.4, 27.9, 27.7, 27.6, 25.1, 24.6, 22.4. HRMS (ESI) for $C_{28}H_{43}N_9NaO_{10}S$ ($[M+Na]^+$): calcd 720.2751, found 720.2744. HPLC Purity (220 nm): 98.4%.

(17*S*,21*S*)-11,19-dioxo-1-(4-(2-(5-((3a*S*,4*S*,6a*R*)-2-oxohexahydro-1*H*-thieno[3,4-*d*]imidazol-4-yl)pentanamido)ethyl)-1*H*-1,2,3-triazol-1-yl)-3,6,9-trioxa-12,18,20-triazatricosane-17,21,23-tricarboxylic acid (L2-biotin)

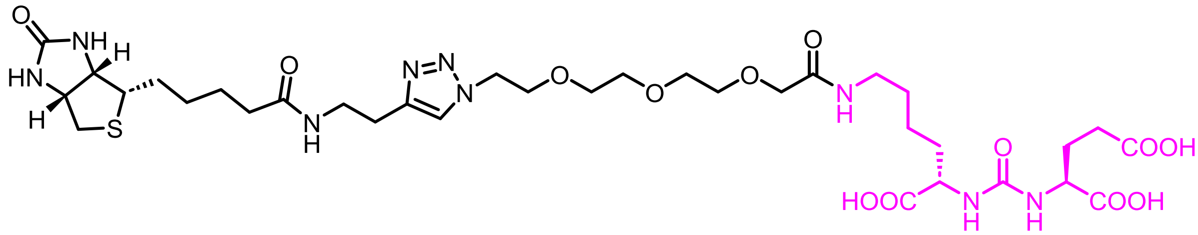

L2-biotin

L2-biotin (3.4 mg, yield 73.1%) was obtained from **S7** (3 mg, 0.0056 mmol) according to the "*General method for click chemistry*".

^{1}H NMR (400 MHz, D_2O) δ 7.81 (s, 1H), 4.58 – 4.48 (m, 3H), 4.33 (dd, *J* = 7.9, 4.4 Hz, 1H), 3.96 (s, 2H), 3.95 – 3.86 (m, 4H), 3.65 – 3.54 (m, 8H), 3.42 (t, *J* = 6.6 Hz, 2H), 3.22 (dt, *J* = 9.8, 5.2 Hz, 1H), 3.13 (t, *J* = 7.0 Hz, 2H), 2.92 (dd, *J* = 13.1, 5.0 Hz, 1H), 2.84 (t, *J* = 6.6 Hz, 2H), 2.70 (d, *J* = 13.1 Hz, 1H), 2.20 – 2.08 (m, 4H), 1.98 – 1.85 (m, 1H), 1.82 – 1.71 (m, 1H), 1.70 – 1.38 (m, 8H), 1.32-1.15 (m, 4H). ^{13}C NMR (100 MHz, D_2O) δ 182.6, 180.6, 180.1, 176.6, 172.2, 159.1, 145.2, 124.0, 70.3, 69.6, 69.5, 68.8, 62.1, 60.2, 55.5, 55.4, 55.3, 49.9, 39.7, 38.8, 38.6, 35.4, 34.1, 32.1, 29.5, 28.2, 27.7, 27.6, 25.1, 24.7, 22.5. HRMS (ESI) for $C_{34}H_{55}N_9NaO_{13}S$ ($[M+Na]^+$): calcd 852.3538, found 852.3523. HPLC Purity (220 nm): 99.5%.

(24*S*,28*S*)-11,18,26-trioxo-1-(4-(2-(5-((3a*S*,4*S*,6a*R*)-2-oxohexahydro-1*H*-thieno[3,4-*d*]imidazol-4-yl)pentanamido)ethyl)-1*H*-1,2,3-triazol-1-yl)-3,6,9-trioxa-12,19,25,27-tetraazatriacontane-24,28,30-tricarboxylic acid (L3-biotin)

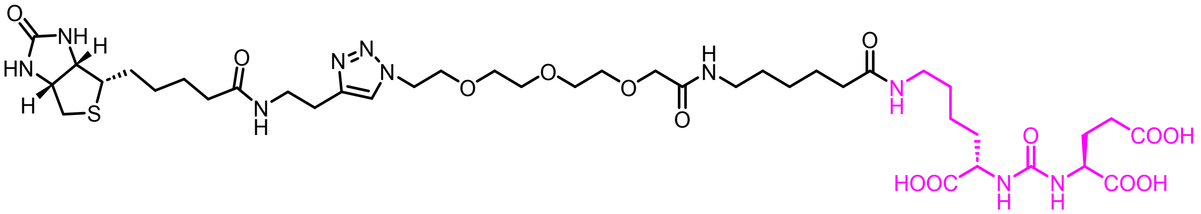

L3-biotin

L3-biotin (8.2 mg, yield 70.7%) was obtained from **S12** (8.0 mg, 0.012 mmol) according to the "*General method for click chemistry*".

^{1}H NMR (400 MHz, D_2O) δ 7.81 (s, 1H), 4.58 – 4.49 (m, 3H), 4.33 (dd, *J* = 7.9, 4.5 Hz, 1H), 4.02 – 3.84 (m, 6H), 3.66 – 3.54 (m, 8H), 3.42 (t, *J* = 6.7 Hz, 2H), 3.21 (dt, *J* = 9.8, 5.1 Hz, 1H), 3.17-3.03 (m, 4H), 2.92 (dd, *J* = 13.1, 5.0 Hz, 1H), 2.84 (t, *J* = 6.6 Hz, 2H), 2.70 (d, *J* = 13.1 Hz, 1H), 2.21-2.06 (m, 6H), 1.99-1.85 (m, 1H), 1.83 – 1.38 (m, 14H), 1.34 – 1.15 (m, 6H). ^{13}C NMR (100 MHz, D_2O) δ 182.5, 180.7, 180.2, 176.6, 172.2, 165.3, 159.2, 145.2, 124.0, 70.4, 69.6, 69.5, 68.8, 62.1, 60.2, 55.5, 55.4, 55.3, 49.9, 39.7, 39.2, 38.8, 38.6, 35.7, 35.4, 34.1, 32.1, 29.4, 28.1, 27.8, 27.7, 25.5, 25.1, 24.7, 22.5. HRMS (ESI) for $C_{40}H_{66}N_{10}Na_2O_{14}S$ ($[M+2Na]^{2+}$): calcd 494.2138, found 494.2123. HPLC Purity (220 nm): 95.0%.

(30*S*,34*S*)-17,24,32-trioxo-1-(4-(2-(5-((3a*S*,4*S*,6a*R*)-2-oxohexahydro-1*H*-thieno[3,4-*d*]imidazol-4-yl)pentanamido)ethyl)-1*H*-1,2,3-triazol-1-yl)-3,6,9,12,15-pentaoxa-18,25,31,33-tetraazahexatriacontane-30,34,36-tricarboxylic acid (L4-biotin)

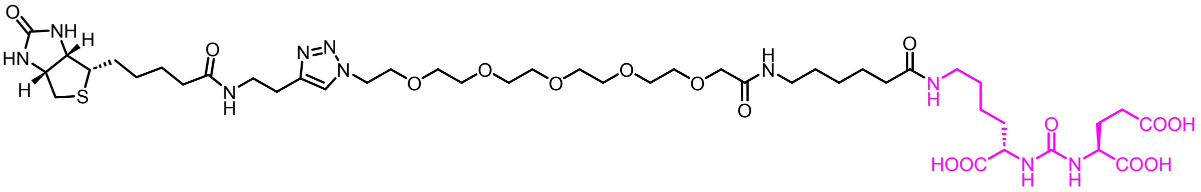

L4-biotin

L4-biotin (7.5 mg, yield 65.2%) was obtained from **S15** (8.2 mg, 0.011 mmol) according to the "*General method for click chemistry*".

^{1}H NMR (400 MHz, D_2O) δ 4.57 – 4.47 (m, 3H), 4.32 (dd, *J* = 7.9, 4.5 Hz, 1H), 4.17 (dd, *J* = 9.0, 5.1 Hz, 1H), 4.10 (dd, *J* = 8.7, 5.0 Hz, 1H), 3.97 (s, 2H), 3.91 – 3.84 (m, 2H), 3.70 – 3.49 (m, 17H), 3.42 (t, *J* = 6.7 Hz, 2H), 3.21 (dt, *J* = 9.7, 5.2 Hz, 1H), 3.15 (t, *J* = 7.0 Hz, 2H), 3.09 (t, *J* = 6.7 Hz, 2H), 2.91 (dd, *J* = 13.0, 5.0 Hz, 1H), 2.85 (t, *J* = 6.6 Hz, 2H), 2.70 (d, *J* = 13.0 Hz, 1H), 2.42 (t, *J* = 7.3 Hz, 2H), 2.22 – 2.02 (m, 5H), 1.89 (ddt, *J* = 14.2, 9.0, 7.1 Hz, 1H), 1.82 – 1.69 (m, 1H), 1.69 – 1.56 (m, 2H), 1.56 – 1.38 (m, 9H), 1.38 – 1.27 (m, 2H), 1.27 – 1.15 (m, 4H). ^{13}C NMR (100 MHz, D_2O) δ 177.3, 176.6, 176.6, 172.2, 165.3, 159.2, 145.2, 124.0, 70.3, 69.7, 69.6, 69.6, 69.6, 69.5, 69.5, 68.8, 62.1, 60.2, 55.3, 49.9, 39.7, 38.9, 38.8, 38.6, 35.7, 35.4, 30.7, 30.1, 28.1, 27.8, 27.8, 27.7, 26.4, 25.5, 25.1, 25.1, 24.7, 22.3. HRMS (ESI) for $C_{44}H_{76}N_{10}O_{16}S$ ($[M+2H]^{2+}$): calcd 516.2581, found 516.2565. HPLC Purity (220 nm): 96.3%.

(9*S*,13*S*)-1-(4-(2-(((*S*)-1-(((*S*)-1-(((*S*)-5-amino-1-carboxypentyl)amino)-3-carboxy-1-oxopropan-2-yl)amino)-3-carboxy-1-oxopropan-2-yl)amino)-2-oxoethyl)phenyl)-3,11-dioxo-2,4,10,12-tetraazapentadecane-9,13,15-tricarboxylic acid (L5)

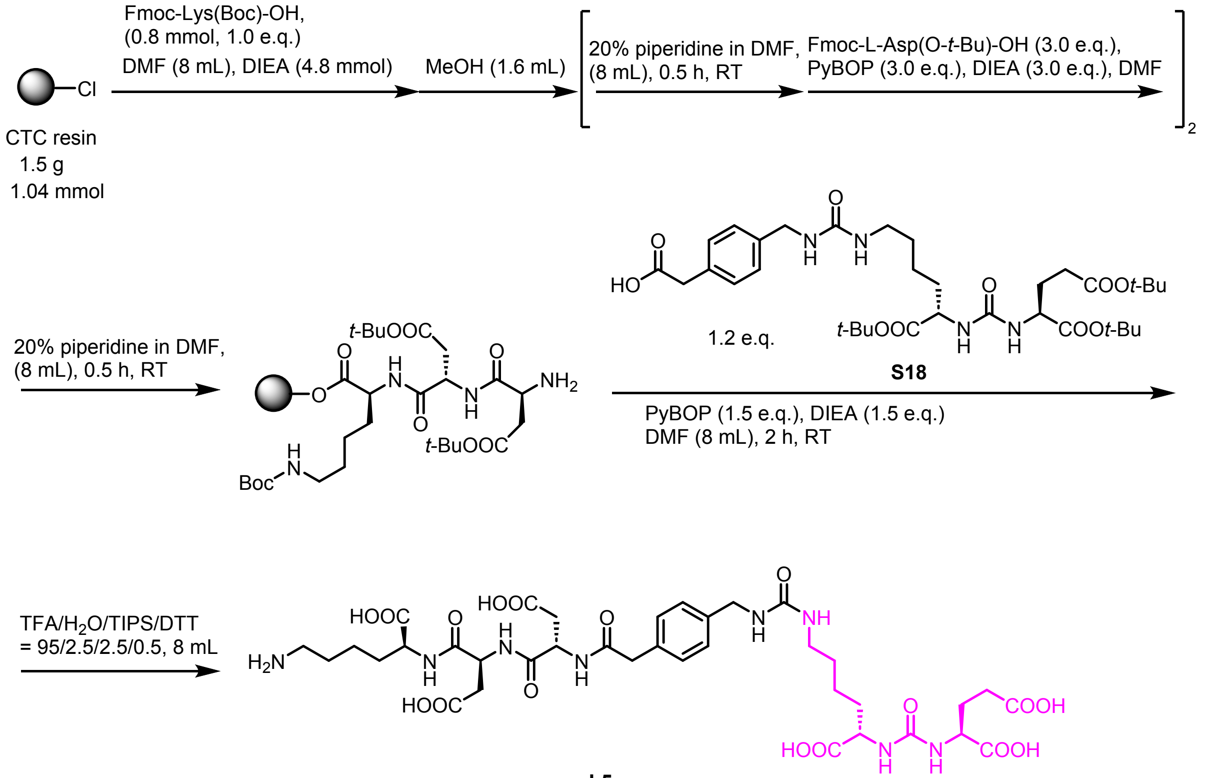

L5

L5 was synthesized via solid phase peptide synthesis using an Fmoc protection strategy. A CTC resin (0.69 mmol/g) was used, a 20% piperidine in DMF solution for Fmoc deprotection, and PyBOP/DIEA chemistry used for all amide conjugations. Peptides were washed with DMF × 5 and DCM × 3 in-between all steps, and ninhydrin testing was done to measure conjugation completion. **S18** was synthesized following reported paper[2]. After the deprotection by TFA/H_2O/TIPS/DTT, the result mixture was poured into Et_2O (50 mL). The precipitate was collected by centrifugation and purified by preparative HPLC C18 reverse column eluting with acetonitrile/water (0.1% acetic acid). 476.6 mg of **L5** was obtained, with a yield of 68.6%, based on Fmoc-Lys(Boc)-OH.

^{1}H NMR (400 MHz, D_2O) δ 7.32 – 7.18 (m, 4H), 4.69 (ddd, J = 7.4, 5.8, 2.8 Hz, 2H), 4.31 – 4.17 (m, 4H), 4.13 (dd, J = 8.6, 5.0 Hz, 1H), 3.62 (s, 2H), 3.09 (t, J = 6.6 Hz, 2H), 2.98 – 2.86 (m, 3H), 2.85 – 2.74 (m, 3H), 2.46 (t, J = 7.4 Hz, 2H), 2.20 – 2.08 (m, 1H), 1.99 – 1.87 (m, 1H), 1.87 – 1.73 (m, 2H), 1.73 – 1.54 (m, 4H), 1.52 – 1.41 (m, 2H), 1.41 – 1.25 (m, 4H). ^{13}C NMR (100 MHz, D_2O) δ 178.4, 178.2, 177.6, 176.9, 175.3, 175.2, 175.1, 173.0, 172.5, 161.2, 159.9, 139.3, 134.1, 130.0, 127.9, 54.2, 54.0, 53.7, 51.2, 50.8, 43.7, 42.3, 40.2, 39.9, 36.2, 36.0, 31.5, 30.9, 30.8, 29.4, 27.3, 26.8, 22.9, 22.5. HRMS (ESI) for $C_{36}H_{53}N_8O_{17}$ ($[M+H]^+$): calcd 869.3529, found 869.3520.

(9*S*,13*S*)-1-(4-((4*S*,7*S*,10*S*)-10-carboxy-4,7-bis(carboxymethyl)-2,5,8,16-tetraoxo-17-(4-(2-(5-((3a*S*,4*S*,6a*R*)-2-oxohexahydro-1*H*-thieno[3,4-*d*]imidazol-4-yl)pentanamido)ethyl)-1*H*-1,2,3-triazol-1-yl)-3,6,9,15-tetraazaheptadecyl)phenyl)-3,11-dioxo-2,4,10,12-tetraazapentadecane-9,13,15-tricarboxylic acid (L5-biotin)

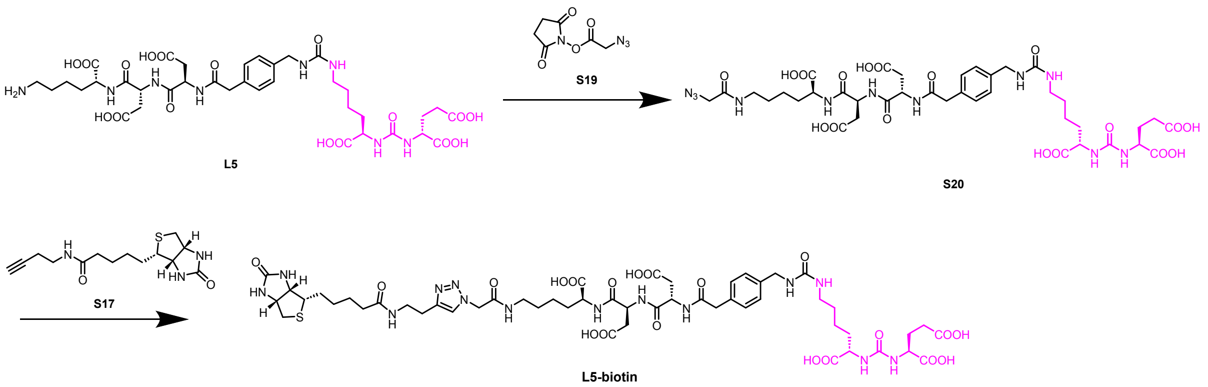

The synthesis of **L5-biotin** starts from **L5**. **L5** (17.4 mg, 0.02 mmol, 1.0 e.q.), **S19** (4.7 mg, 0.024 mmol) and triethylamine (11 µL, 0.08 mmol) were dissolved in DMF/water (5/1, 1.2 mL). The result mixture was stirred at RT for 2 h and then co-distilled with acetonitrile 3 times. Ethyl ether (5 mL) was added and the precipitate containing **S20** was collected and directly used for the next step. The crude **S20** was then reacted with **S17** (6.5 mg, 0.022 mmol) according to the "*General method for click chemistry*" to afford **L5-biotin** (9.6 mg, 38.6% in two steps)

^{1}H NMR (400 MHz, DMSO-d$_6$) δ 8.33 (d, *J* = 7.6 Hz, 1H), 8.25 (t, *J* = 5.5 Hz, 1H), 8.17 (d, *J* = 8.1 Hz, 1H), 7.83 (t, *J* = 5.7 Hz, 1H), 7.75 (s, 1H), 7.72 (d, *J* = 7.8 Hz, 1H), 7.14 – 7.03 (m, 4H), 6.35 (s, 1H), 6.32 – 6.21 (m, 3H), 6.18 (t, *J* = 6.1 Hz, 1H), 5.84 (t, *J* = 5.7 Hz, 1H), 4.95 (s, 2H), 4.55 – 4.41 (m, 2H), 4.24 (dd, *J* = 7.8, 5.0 Hz, 1H), 4.13 – 3.92 (m, 6H), 3.35 (q, *J* = 14.3 Hz, 2H), 3.22 (q, *J* = 7.3 Hz, 2H), 3.07 – 2.96 (m, 3H), 2.91 (q, *J* = 6.4 Hz, 2H), 2.75 (dd, *J* = 12.4, 5.1 Hz, 1H), 2.68 (t, *J* = 7.4 Hz, 2H), 2.65 – 2.55 (m, 2H), 2.54 – 2.47 (m, 2H), 2.40 (dd, *J* = 7.7, 3.6 Hz, 1H), 2.24 – 2.08 (m, 2H), 1.98 (t, *J* = 7.5 Hz, 2H), 1.89 – 1.76 (m, 1H), 1.73 – 1.12 (m, 19H). ^{13}C NMR (125 MHz, DMSO-d$_6$) δ 175.2, 174.9, 174.5, 174.0, 172.7, 172.6, 171.4, 171.1, 171.0, 165.9, 163.4, 158.7, 157.9, 144.8, 139.5, 135.0, 129.5, 127.6, 124.3, 61.7, 59.8, 56.0, 53.0, 52.8, 52.5, 52.2, 50.4, 50.1, 43.3, 42.3, 39.2, 39.0, 37.2, 36.7, 35.8, 32.5, 31.3, 30.8, 30.4, 29.0, 28.8, 28.7, 28.4, 26.2, 25.9, 23.3. HRMS (ESI) for $C_{52}H_{74}N_{14}Na_2O_{20}S$ ([M+2Na]$^{2+}$): calcd 646.2360, found 646.2346. HPLC Purity (220 nm): 99.5%.

Synthetic procedures for the preparation of L3-DBCO and L5-DBCO

(3*S*,7*S*)-5,13,20,32,35-pentaoxo-35-(DBCO)-22,25,28-trioxa-4,6,12,19,31-pentaazapentatriacontane-1,3,7-tricarboxylic acid (L3-DBCO)

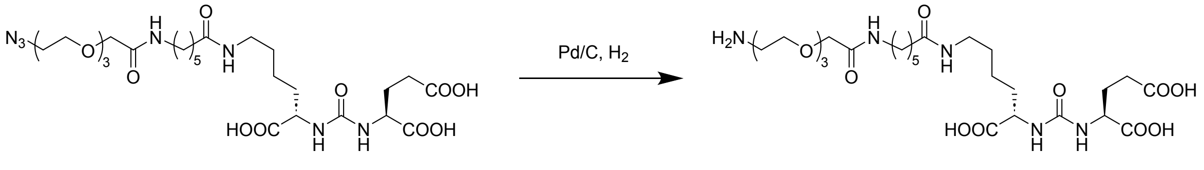

S12 S21

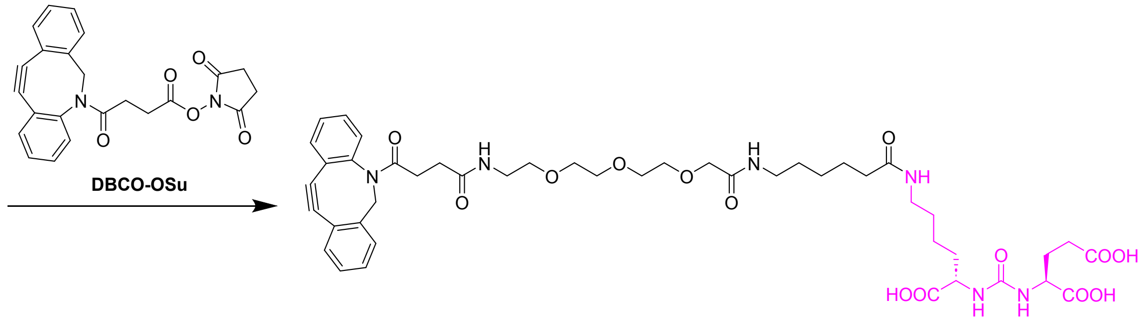

L3-DBCO

Pd/C (5%, 8 mg) was added to a mixture of **S12** (8 mg, 0.012 mmol, 1.0 e.q.) in EtOH/water (10/1, 1.1 mL) under a nitrogen atmosphere. The reaction flask was degassed and charged with a hydrogen balloon. The reaction was stirred at RT 3 h. The mixture was filtered and concentrated, and the product was directly used in the next step. Crude amine from above, **DBCO-OSu** (9.6 mg, 0.024 mmol) and triethylamine (6.8 μL, 0.049 mmol) were dissolved in DMF (300 μL). The result mixture was stirred at RT for 2 h. Ethyl ether (1.5 mL) was added and the precipitate was washed with ethyl ether once more. The residue was taken up with ACN/water and lyophilized to afford **L3-DBCO** (5.0 mg, yield 44.6%).

^{1}H NMR (400 MHz, DMSO-d$_6$) δ 7.75 (dt, *J* = 18.1, 5.6 Hz, 2H), 7.71 – 7.60 (m, 3H), 7.55 – 7.42 (m, 3H), 7.42 – 7.28 (m, 3H), 6.35 (d, *J* = 8.1 Hz, 1H), 6.28 (d, *J* = 7.9 Hz, 1H), 5.04 (d, *J* = 14.0 Hz, 1H), 4.12 – 3.96 (m, 2H), 3.85 (s, 2H), 3.62 (d, *J* = 13.9 Hz, 1H), 3.58 – 3.53 (m, 4H), 3.53 – 3.49 (m, 2H), 3.49 – 3.44 (m, 2H), 3.33 – 3.29 (m, 1H), 3.14 – 3.03 (m, 4H), 2.99 (q, *J* = 6.6 Hz, 2H), 2.29 – 2.19 (m, 3H), 2.02 (t, *J* = 7.4 Hz, 3H), 1.84 – 1.72 (m, 3H), 1.70 – 1.57 (m, 1H), 1.57 – 1.32 (m, 8H), 1.32 – 1.14 (m, 5H). ^{13}C NMR (125 MHz, DMSO-d$_6$) δ 175.3, 174.8, 174.6, 172.5, 171.8, 171.7, 169.6, 157.8, 152.2, 149.1, 133.1, 130.3, 129.6, 128.8, 128.6, 128.3, 127.4, 125.8, 123.2, 122.1, 108.8, 70.8, 70.6, 70.3, 70.2, 69.6, 55.5, 53.0, 52.6, 41.1, 39.1, 38.9, 38.6, 36.0, 32.4, 31.0, 30.3, 29.6, 29.5, 28.9, 26.7, 25.9, 25.7, 23.3. HRMS (ESI) for $C_{45}H_{60}N_6NaO_{14}$ ([M+Na]$^+$): calcd 931.4065, found 931.4037. HPLC Purity (254 nm): 99.4%.

(9*S*,13*S*)-1-(4-((4*S*,7*S*,10*S*)-10-carboxy-4,7-bis(carboxymethyl)-2,5,8,16,19-pentaoxo-19-(DBCO)-3,6,9,15-tetraazanonadecyl)phenyl)-3,11-dioxo-2,4,10,12-tetraazapentadecane-9,13,15-tricarboxylic acid (L5-DBCO)

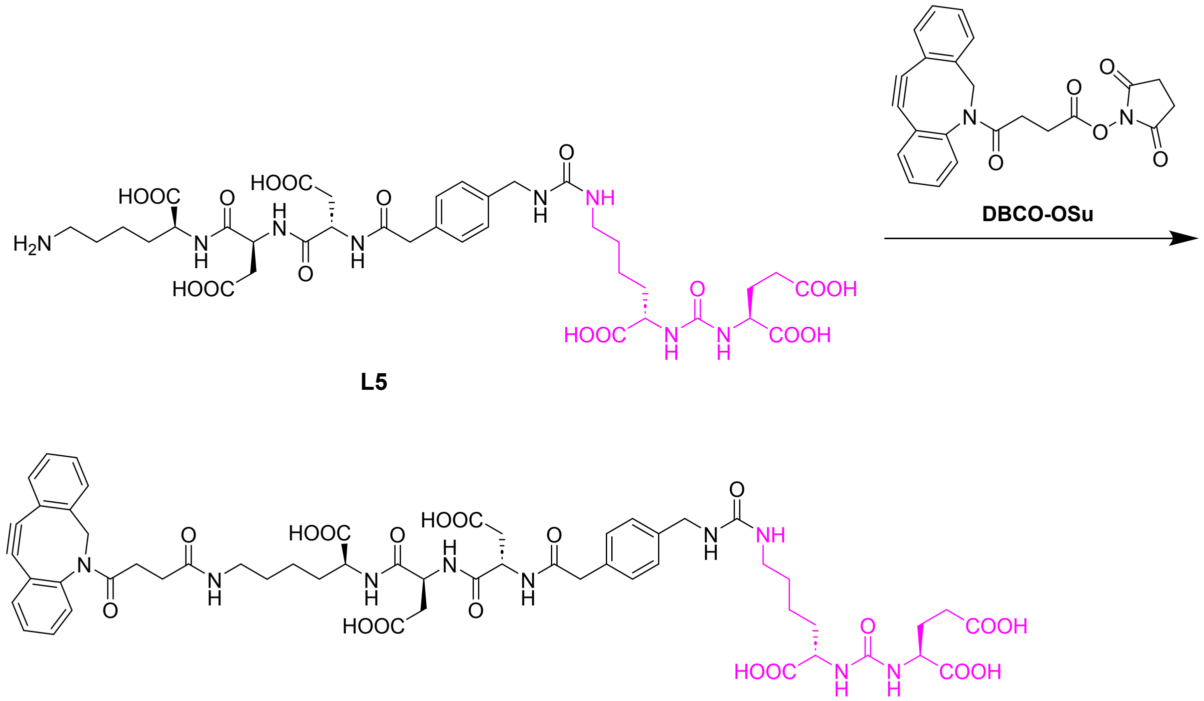

L5-DBCO

L5 (17.4 mg, 0.02 mmol, 1.0 e.q.), **DBCO-OSu** (9.6 mg, 0.024 mmol) and triethylamine (13.5 µL, 0.098 mmol) were dissolved in DMF/water (5/1, 1.2 mL). The result mixture was stirred at RT for 2 h and then co-distilled with acetonitrile 3 times. Ethyl ether (5 mL) was added and the precipitate was washed with ethyl ether once more. The residue was taken up with ACN/water and lyophilized to afford **L5-DBCO** (21.5 mg, yield 93.1%).

^{1}H NMR (400 MHz, DMSO-d$_6$) δ 8.32 (d, *J* = 7.6 Hz, 1H), 8.14 (d, *J* = 8.2 Hz, 1H), 7.68 (d, *J* = 7.7 Hz, 1H), 7.65 – 7.58 (m, 2H), 7.56 (dd, *J* = 7.3, 1.6 Hz, 1H), 7.46 – 7.35 (m, 3H), 7.34 – 7.20 (m, 3H), 7.14 – 7.03 (m, 4H), 6.25 (d, *J* = 8.3 Hz, 2H), 6.18 (t, *J* = 6.0 Hz, 1H), 5.84 (t, *J* = 5.7 Hz, 1H), 4.96 (d, *J* = 14.0 Hz, 1H), 4.54 – 4.40 (m, 2H), 4.08 (d, *J* = 5.7 Hz, 2H), 4.05 – 3.93 (m, 3H), 3.54 (d, *J* = 14.0 Hz, 1H), 3.42 – 3.26 (m, 2H), 2.93 – 2.88 (m, 2H), 2.87 – 2.83 (m, 1H), 2.65 – 2.47 (m, 4H), 2.42 – 2.35 (m, 2H), 2.21 – 2.10 (m, 3H), 1.97 – 1.76 (m, 2H), 1.75 – 1.52 (m, 4H), 1.45 (dq, *J* = 14.1, 6.7 Hz, 2H), 1.35 – 1.11 (m, 8H). ^{13}C NMR (100 MHz, DMSO-d$_6$) δ 175.1, 174.8, 174.3, 173.8, 172.3, 171.6, 171.3, 171.1, 170.9, 170.8, 158.5, 157.8, 152.1, 148.9, 139.3, 134.8, 132.9, 130.1, 129.4, 129.4, 128.6, 128.5, 128.1, 127.4, 127.2, 125.6, 123.0, 121.9, 114.7, 108.6, 55.4, 52.9, 52.6, 52.3, 50.2, 49.9, 43.2, 42.1, 38.8, 36.9, 36.5, 32.4, 31.1, 30.9, 30.6, 30.2, 29.1, 28.2, 23.1. HRMS (ESI) for $C_{55}H_{65}N_9NaO_{19}$ ([M+Na]$^+$): calcd 1178.4294, found 1178.4279. HPLC Purity (254 nm): 97.2%.

Synthetic procedures for the preparation of BMS-L5-1, BMS-L5-2 and BMS-L5-3

***N*-(2-(1-(2-(2-azidoethoxy)ethyl)-1*H*-1,2,3-triazol-4-yl)ethyl)-1-(3-bromo-4-((2-methyl-[1,1'-biphenyl]-3-yl)methoxy)benzyl)piperidine-2-carboxamide (S27), *N*-(2-(1-(2-(2-(2-(2-azidoethoxy)ethoxy)ethoxy)ethyl)-1*H*-1,2,3-triazol-4-yl)ethyl)-1-(3-bromo-4-((2-methyl-[1,1'-biphenyl]-3-yl)methoxy)benzyl)piperidine-2-carboxamide (S28) and *N*-(2-(1-(23-azido-3,6,9,12,15,18,21-heptaoxatricosyl)-1*H*-1,2,3-triazol-4-yl)ethyl)-1-(3-bromo-4-((2-methyl-[1,1'-biphenyl]-3-yl)methoxy)benzyl)piperidine-2-carboxamide (S29)**

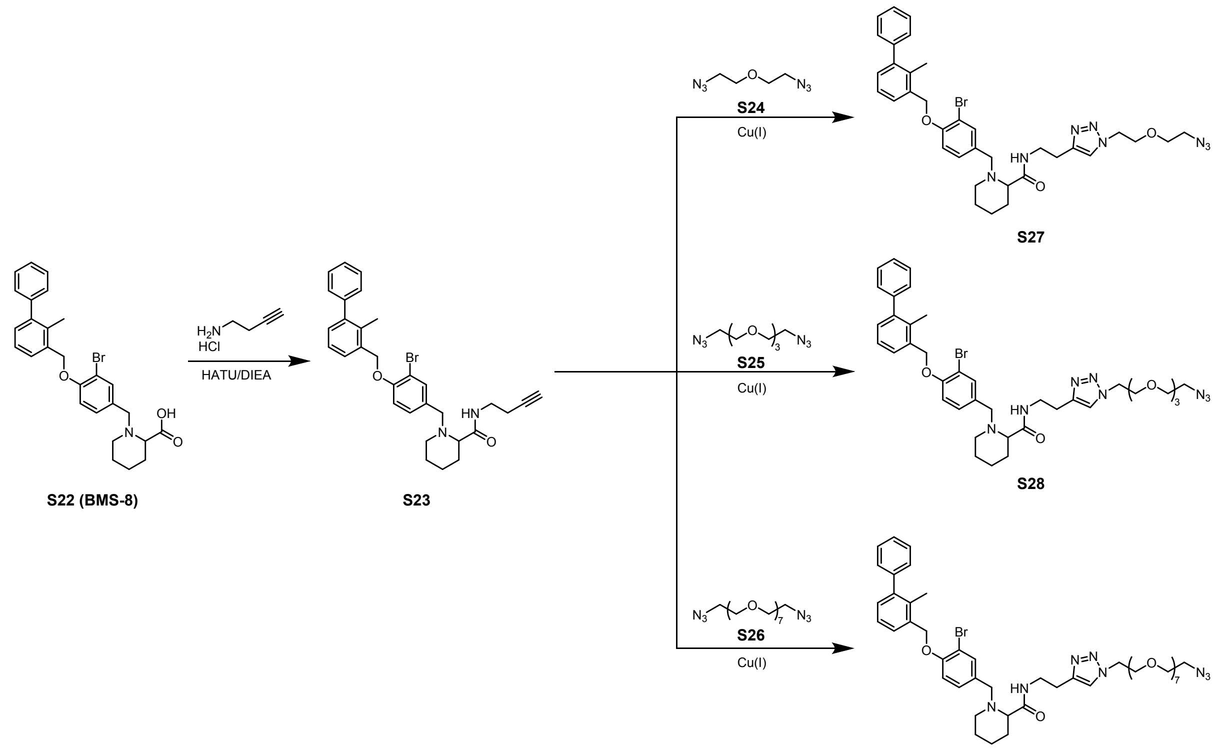

HATU (57 mg, 0.15 mmol) was added to a mixture of 3-butyn-1-amine, hydrochloride (105 mg, 1 mmol, 1.0 e.q.), **BMS-8** (49.4 mg, 0.1 mmol) and DIEA (52.3 μL, 0.3 mmol) in DMF (1 mL). The reaction was stirred at RT until LCMS indicated the end. The result mixture was diluted with EA and washed with water three times. The organic layer was dried over sodium sulfate, filtered and concentrated to afford crude **S23** (59.1 mg).

Sodium ascorbate (3.6 mg, 0.018 mmol) was added to a mixture of **S23** (10 mg, 0.018 mmol, 1.0 e.q.), **S24** (14 mg, 0.09 mmol), copper(II) sulfate (1 mg, 0.0062 mmol), and Tris[(1-benzyl-1H-1,2,3-triazol-4-yl)methyl]amine (3.2 mg, 0.0062 mmol) in DMF/water (10/1, 1.1 mL). The reaction was stirred at RT until LCMS indicated the end and then purified by preparative HPLC C18 reverse column eluting with acetonitrile/water (0.1% formic acid) to afford **S27** (5.3 mg).

S28 (6.5 mg) and **S29** (7.2 mg) were obtained from **S23** (10 mg, 0.018 mmol) using the procedure described for **S27**.

(9*S*,13*S*)-1-(4-((4*S*,7*S*,10*S*)-18-(1-(2-(2-(4-(2-(1-(3-bromo-4-((2-methyl-[1,1'-biphenyl]-3-yl)methoxy)benzyl)piperidine-2-carboxamido)ethyl)-1*H*-1,2,3-triazol-1-yl)ethoxy)ethyl)-1*H*-1,2,3-triazol-4-yl)-10-carboxy-4,7-bis(carboxymethyl)-2,5,8,16-tetraoxo-3,6,9,15-tetraazaoctadecyl)phenyl)-3,11-dioxo-2,4,10,12-tetraazapentadecane-9,13,15-tricarboxylic acid (BMS-L5-1)

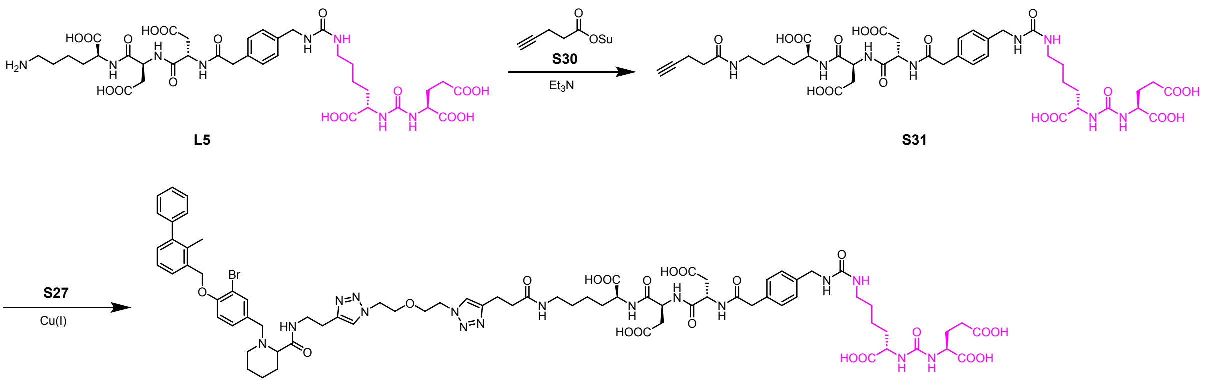

BMS-L5-1

To a solution of **L5** (45 mg, 0.052 mmol) and **S30** (10.2 mg, 0.052 mmol) in DMF/H_2O (10/1, 1.1 mL) was added triethylamine (50 μL, 0.36 mmol). The mixture was stirred for 2 h at RT. The reaction mixture was co-distilled with acetonitrile (2 mL*3) and poured into ethyl ether (6 mL). The residue was washed with ethyl ether one more time and dispersed in water and lyophilized afford a white solid **S44** (53 mg, yield 107.5%). ^{1}H NMR (400 MHz, DMSO-d$_6$) δ 8.40 (d, J = 7.6 Hz, 1H), 8.23 (d, J = 8.1 Hz, 1H), 7.88 (t, J = 5.6 Hz, 1H), 7.77 (d, J = 7.7 Hz, 1H), 7.23 – 7.09 (m, 4H), 6.40 – 6.20 (m, 3H), 5.92 (t, J = 5.6 Hz, 1H), 4.61 – 4.48 (m, 2H), 4.15 (d, J = 5.6 Hz, 2H), 4.12 – 4.00 (m, 3H), 3.43 (q, J = 14.3 Hz, 2H), 3.01 (dq, J = 16.2, 6.8 Hz, 4H), 2.73 (t, J = 2.6 Hz, 1H), 2.72 – 2.62 (m, 2H), 2.46 (d, J = 7.8 Hz, 1H), 2.38 – 2.31 (m, 2H), 2.29 – 2.20 (m, 4H), 1.95 – 1.81 (m, 1H), 1.79 – 1.44 (m, 5H), 1.43 – 1.32 (m, 4H), 1.32 – 1.22 (m, 4H).^{13}C NMR (100 MHz, DMSO-d$_6$) δ 175.1, 174.8, 174.4, 173.8, 173.3, 172.4, 171.2, 170.9, 170.8, 170.5, 158.5, 157.8, 139.3, 134.8, 129.4, 127.4, 84.3, 71.7, 52.9, 52.7, 52.4, 50.2, 49.9, 43.2, 42.1, 40.9, 38.8, 37.0, 36.6, 34.7, 32.4, 31.2, 30.7, 30.2, 29.1, 28.3, 25.7, 23.1, 23.1, 14.8.

Sodium ascorbate (2 mg, 0.01 mmol) was added to a mixture of **S31** (8.6 mg, 0.0091 mmol), **S27** (5.3 mg, 0.0076 mmol), copper(II) sulfate (1 mg, 0.0062 mmol), and Tris[(1-benzyl-1H-1,2,3-triazol-4-yl)methyl]amine (3.2 mg, 0.0062 mmol) in DMF/water (5/1, 1.2 mL). The reaction was stirred at RT until LCMS indicated the end and then purified by preparative HPLC C18 reverse column eluting with acetonitrile/water (0.1% formic acid) to afford **BMS-L5-1** (7.9 mg, 63.1% in two steps).

^{1}H NMR (500 MHz, DMSO-d$_6$) δ 8.39 (d, J = 7.7 Hz, 1H), 8.24 (d, J = 8.1 Hz, 1H), 8.02 (t, J = 5.9 Hz, 1H), 7.90 (t, J = 5.6 Hz, 1H), 7.83 (d, J = 7.8 Hz, 1H), 7.70 (s, 1H), 7.67 (s, 1H), 7.58 – 7.51 (m, 2H), 7.47 (t, J = 6.8 Hz, 2H), 7.42 – 7.35 (m, 1H), 7.35 – 7.25 (m, 4H), 7.25 – 7.11 (m, 6H), 6.35 (t, J = 7.7 Hz, 1H), 6.33 – 6.21 (m, 2H), 5.92 (t, J = 5.6 Hz, 1H), 5.23 (s, 2H), 4.62 – 4.48 (m, 2H), 4.47 – 4.33 (m, 4H), 4.15 (d, J = 5.5 Hz, 2H), 4.11 – 3.99 (m, 3H), 3.77 – 3.66 (m, 4H), 3.48 – 3.32 (m, 4H), 3.05 – 2.92 (m, 4H), 2.80 (dt, J = 12.4, 7.3 Hz, 4H), 2.76 – 2.57 (m, 5H), 2.48 – 2.43 (m, 2H), 2.40 (dd, J = 8.8, 6.8 Hz, 2H), 2.28 – 2.16 (m, 5H), 1.95 – 1.79 (m, 2H), 1.79 – 1.60 (m, 5H), 1.52 (tq, J = 12.8, 7.6 Hz, 4H), 1.42 – 1.13 (m, 11H). ^{13}C NMR (125 MHz, DMSO-d$_6$) δ 175.3, 175.0, 174.6, 174.0, 173.7, 172.7, 171.6, 171.3, 171.0, 170.9, 163.8, 158.7, 158.7, 157.9, 154.1, 146.6, 144.9, 142.8, 142.7, 142.0, 139.5, 136.0, 135.0, 134.4, 133.9, 132.9, 130.2, 130.1, 129.8, 129.5, 128.9, 128.2, 127.6, 127.6, 126.2, 123.1, 122.8, 114.2, 111.5, 69.9, 69.2, 69.2, 67.9, 58.8, 53.1, 52.7, 51.4, 50.5, 50.1, 49.7, 43.4, 42.3, 39.0, 38.7, 35.5, 32.6, 31.6, 31.1, 30.3, 30.3, 29.3, 28.7, 26.1, 25.2, 23.8, 23.3, 23.3, 22.0, 16.5. HRMS (ESI) for $C_{76}H_{99}BrN_{16}O_{21}$ ([M+2H]$^{2+}$): calcd 825.3172, found 825.3153. HPLC Purity (220 nm): 98.4%.

(9*S*,13*S*)-1-(4-((4*S*,7*S*,10*S*)-18-(1-(2-(2-(2-(2-(4-(2-(1-(3-bromo-4-((2-methyl-[1,1'-biphenyl]-3-yl)methoxy)benzyl)piperidine-2-carboxamido)ethyl)-1*H*-1,2,3-triazol-1-yl)ethoxy)ethoxy)ethoxy)ethyl)-1*H*-1,2,3-triazol-4-yl)-10-carboxy-4,7-bis(carboxymethyl)-2,5,8,16-

tetraoxo-3,6,9,15-tetraazaoctadecyl)phenyl)-3,11-dioxo-2,4,10,12-tetraazapentadecane-9,13,15-tricarboxylic acid (BMS-L5-2)

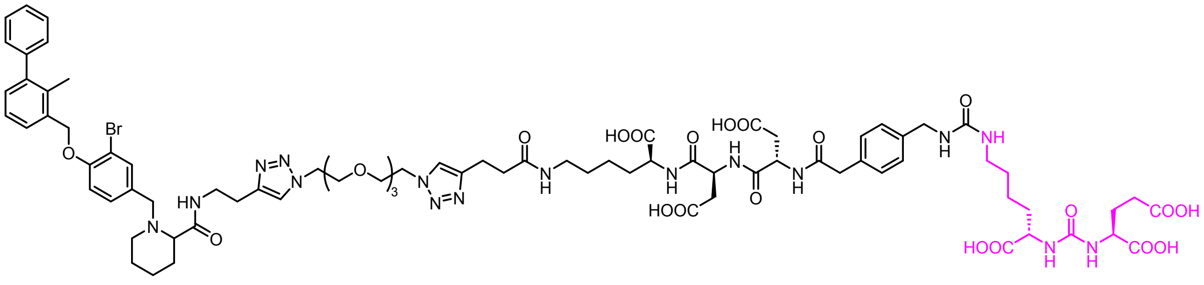

BMS-L5-2

BMS-L5-2 (6.9 mg, 48.4%) was obtained from **S28** (6.5 mg, 0.0082 mmol) using the procedure described for **BMS-L5-1**.

^{1}H NMR (500 MHz, DMSO-d$_6$) δ 8.39 (d, *J* = 7.6 Hz, 1H), 8.23 (d, *J* = 8.2 Hz, 1H), 8.00 (t, *J* = 5.9 Hz, 1H), 7.87 (t, *J* = 5.6 Hz, 1H), 7.81 (s, 2H), 7.75 (s, 1H), 7.57 (d, *J* = 2.0 Hz, 1H), 7.53 (dd, *J* = 7.7, 1.4 Hz, 1H), 7.47 (t, *J* = 7.5 Hz, 2H), 7.42 – 7.36 (m, 1H), 7.35 – 7.25 (m, 4H), 7.25 – 7.10 (m, 6H), 6.42 – 6.21 (m, 3H), 5.91 (t, *J* = 5.7 Hz, 1H), 5.23 (s, 2H), 4.53 (td, *J* = 7.8, 4.1 Hz, 2H), 4.43 (t, *J* = 5.3 Hz, 2H), 4.39 (t, *J* = 5.3 Hz, 2H), 4.15 (d, *J* = 5.6 Hz, 2H), 4.05 (td, *J* = 13.4, 7.7 Hz, 3H), 3.74 (dt, *J* = 12.6, 5.3 Hz, 4H), 3.49 – 3.45 (m, 4H), 3.45 – 3.42 (m, 6H), 3.42 – 3.31 (m, 2H), 3.04 – 2.96 (m, 4H), 2.80 (dt, *J* = 12.8, 7.3 Hz, 4H), 2.75 – 2.61 (m, 5H), 2.49 – 2.43 (m, 2H), 2.40 (t, *J* = 6.8 Hz, 2H), 2.29 – 2.17 (m, 5H), 1.93 – 1.81 (m, 2H), 1.76 – 1.60 (m, 5H), 1.52 (qt, *J* = 13.8, 8.8 Hz, 4H), 1.43 – 1.12 (m, 11H). ^{13}C NMR (126 MHz, DMSO-d$_6$) δ 175.3, 175.0, 174.6, 174.0, 173.7, 172.6, 171.6, 171.3, 171.0, 163.9, 158.7, 157.9, 154.1, 146.6, 144.9, 142.8, 141.9, 139.5, 136.0, 135.0, 134.4, 133.9, 132.9, 130.2, 130.1, 129.8, 129.5, 128.9, 128.2, 127.6, 127.6, 126.2, 123.1, 122.8, 114.2, 111.5, 70.2, 70.2, 70.2, 69.9, 69.4, 69.4, 67.9, 58.8, 53.1, 52.6, 51.4, 50.5, 50.1, 49.8, 43.4, 42.3, 39.0, 38.7, 35.5, 32.6, 31.0, 30.4, 30.3, 29.3, 28.6, 26.1, 25.2, 23.8, 23.3, 23.3, 22.0, 16.5. HRMS (ESI) for $C_{80}H_{107}BrN_{16}O_{23}$ ($[M+2H]^{2+}$): calcd 869.3434, found 869.3408. HPLC Purity (220 nm): 97.3%.

(9*S*,13*S*)-1-(4-((4*S*,7*S*,10*S*)-18-(1-(23-(4-(2-(1-(3-bromo-4-((2-methyl-[1,1'-biphenyl]-3-yl)methoxy)benzyl)piperidine-2-carboxamido)ethyl)-1*H*-1,2,3-triazol-1-yl)-3,6,9,12,15,18,21-heptaoxatricosyl)-1*H*-1,2,3-triazol-4-yl)-10-carboxy-4,7-bis(carboxymethyl)-2,5,8,16-tetraoxo-3,6,9,15-tetraazaoctadecyl)phenyl)-3,11-dioxo-2,4,10,12-tetraazapentadecane-9,13,15-tricarboxylic acid (BMS-L5-3)

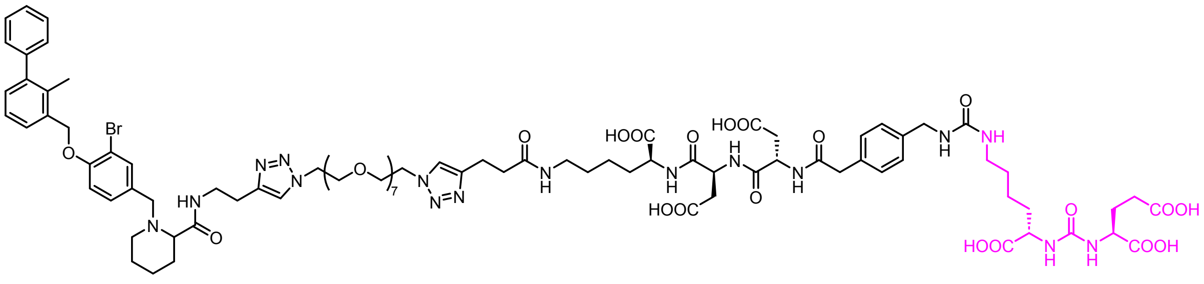

BMS-L5-3

BMS-L5-3 (5.8 mg, 45.1%) was obtained from **S29** (7.2 mg, 0.0074 mmol) using the procedure described for **BMS-L5-1**.

^{1}H NMR (500 MHz, DMSO-d$_6$) δ 8.39 (d, *J* = 7.7 Hz, 1H), 8.24 (d, *J* = 8.1 Hz, 1H), 7.99 (t, *J* = 5.8 Hz, 1H),

7.90 (t, J = 5.6 Hz, 1H), 7.86 – 7.80 (m, 2H), 7.77 (s, 1H), 7.57 (d, J = 1.9 Hz, 1H), 7.54 (d, J = 7.5 Hz, 1H), 7.47 (t, J = 7.5 Hz, 2H), 7.39 (t, J = 7.4 Hz, 1H), 7.35 – 7.26 (m, 4H), 7.25 – 7.12 (m, 6H), 6.37 (d, J = 8.2 Hz, 1H), 6.30 (d, J = 7.6 Hz, 2H), 5.93 (t, J = 5.7 Hz, 1H), 5.23 (s, 2H), 4.58 – 4.49 (m, 2H), 4.45 (t, J = 5.3 Hz, 2H), 4.40 (t, J = 5.3 Hz, 2H), 4.15 (d, J = 5.5 Hz, 2H), 4.12 – 4.00 (m, 3H), 3.75 (dt, J = 16.5, 5.3 Hz, 4H), 3.53 – 3.45 (m, 22H), 3.45 – 3.31 (m, 6H), 3.04 – 2.96 (m, 4H), 2.81 (dt, J = 13.9, 7.5 Hz, 4H), 2.75 – 2.60 (m, 5H), 2.49 – 2.43 (m, 2H), 2.40 (t, J = 6.8 Hz, 2H), 2.26 – 2.19 (m, 5H), 1.92 – 1.78 (m, 2H), 1.78 – 1.44 (m, 9H), 1.42 – 1.12 (m, 11H). ^{13}C NMR (126 MHz, DMSO-d_6) δ 175.3, 175.0, 174.7, 174.0, 173.7, 172.7, 171.6, 171.3, 171.0, 170.9, 158.7, 157.9, 154.1, 146.6, 144.8, 142.8, 141.9, 139.5, 136.0, 135.0, 134.4, 133.9, 133.0, 130.2, 130.1, 129.8, 129.5, 128.9, 128.1, 127.6, 126.2, 123.2, 122.8, 114.2, 111.5, 70.4, 70.3, 70.2, 70.2, 69.9, 69.4, 69.4, 67.9, 58.8, 53.1, 52.8, 51.4, 50.5, 50.1, 49.8, 43.4, 42.3, 39.0, 38.7, 35.5, 32.6, 31.6, 31.2, 30.3, 30.3, 29.3, 28.8, 26.1, 25.2, 23.8, 23.3, 23.3, 22.0, 16.5. HRMS (ESI) for $C_{88}H_{123}BrN_{16}O_{27}$ ($[M+2H]^{2+}$): calcd 957.3958, found 957.3928. HPLC Purity (220 nm): 99.4%.

4. NMR

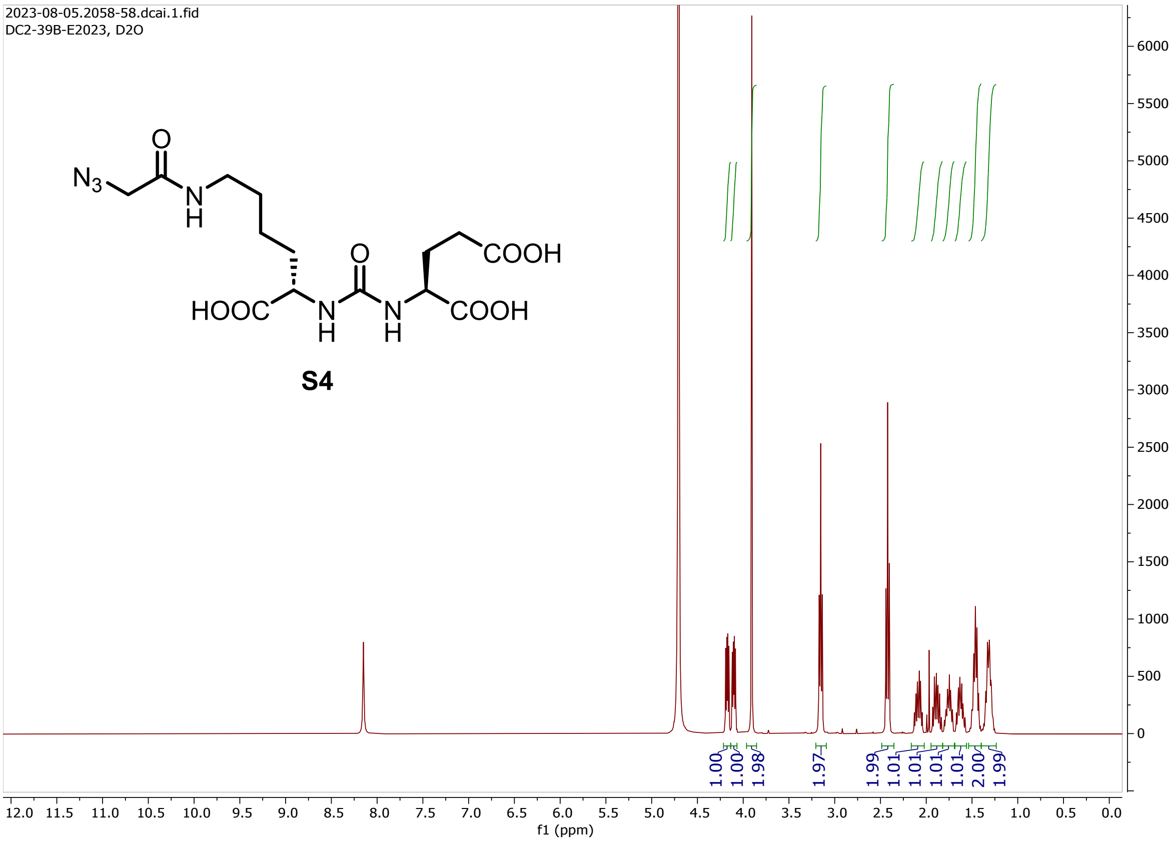

2023-08-05.2058-58.dcai.1.fid
DC2-39B-E2023, D2O
N3
O
N
H
O
COOH
HOOC
N
H
N
H
COOH
1.00
1.00
1.98
1.97
1.99
1.01
1.01
1.01
1.01
2.00
1.99
f1 (ppm)

S4

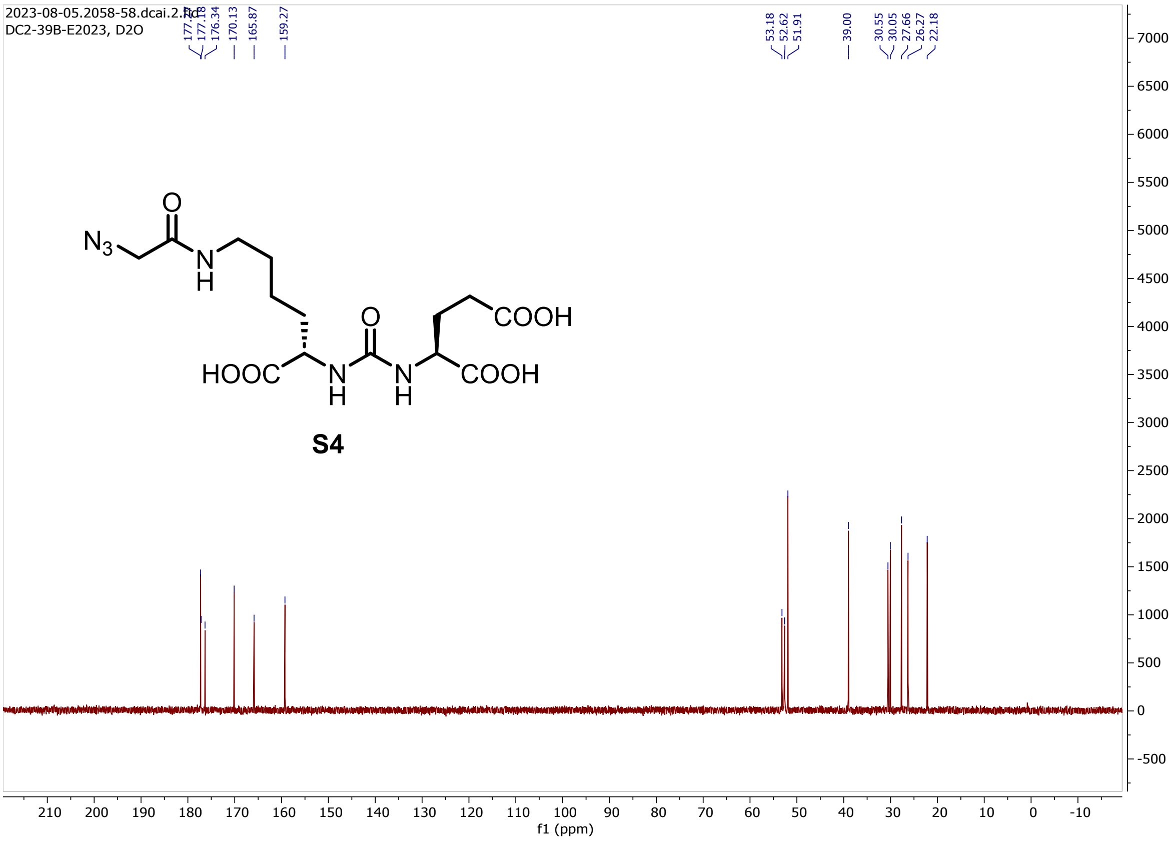

2023-08-05.2058-58.dcai.2.fid
DC2-39B-E2023, D2O
177.18
176.34
170.13
165.87
159.27
53.18
52.62
51.91
39.00
30.55
30.05
27.66
26.27
22.18
N3
O
N
H
O
COOH
HOOC
N
H
N
H
COOH
f1 (ppm)

S4

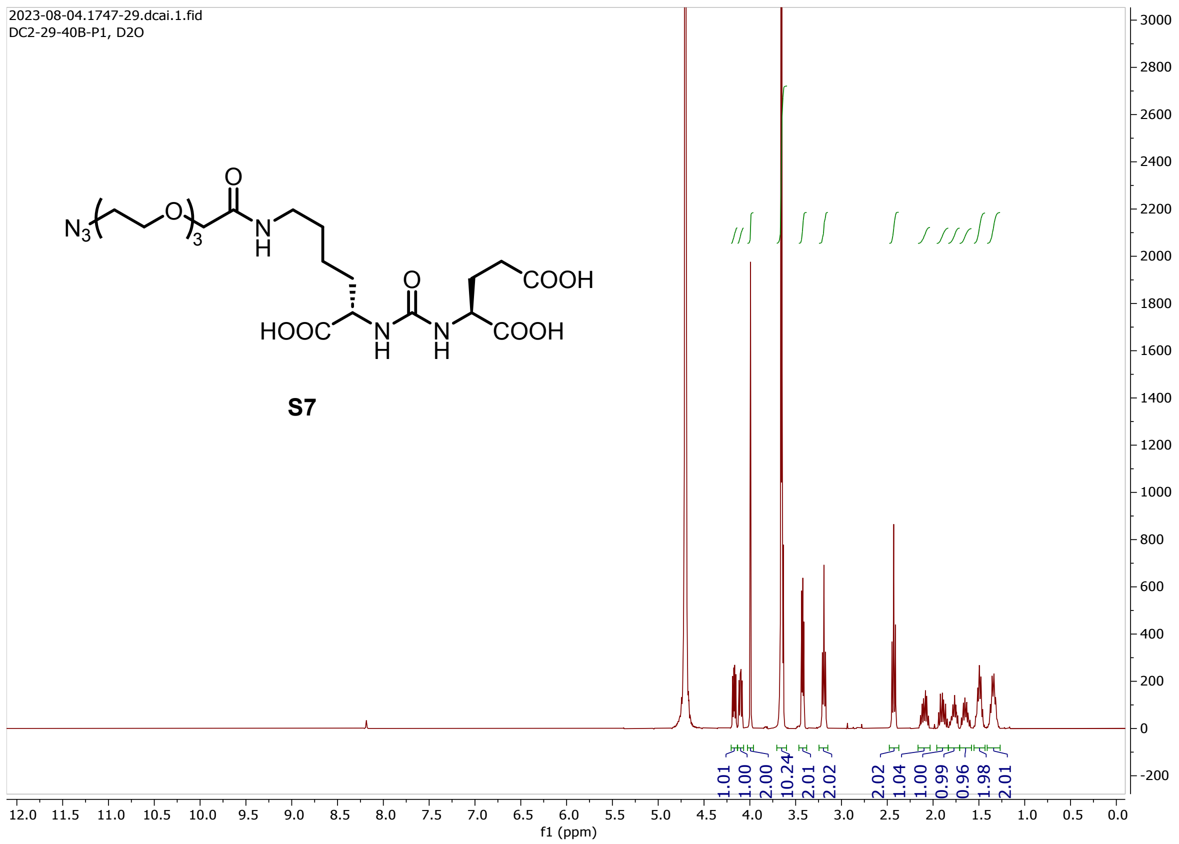
2023-08-04.1747-29.dcai.1.fid
DC2-29-40B-P1, D2O
S7
f1 (ppm)

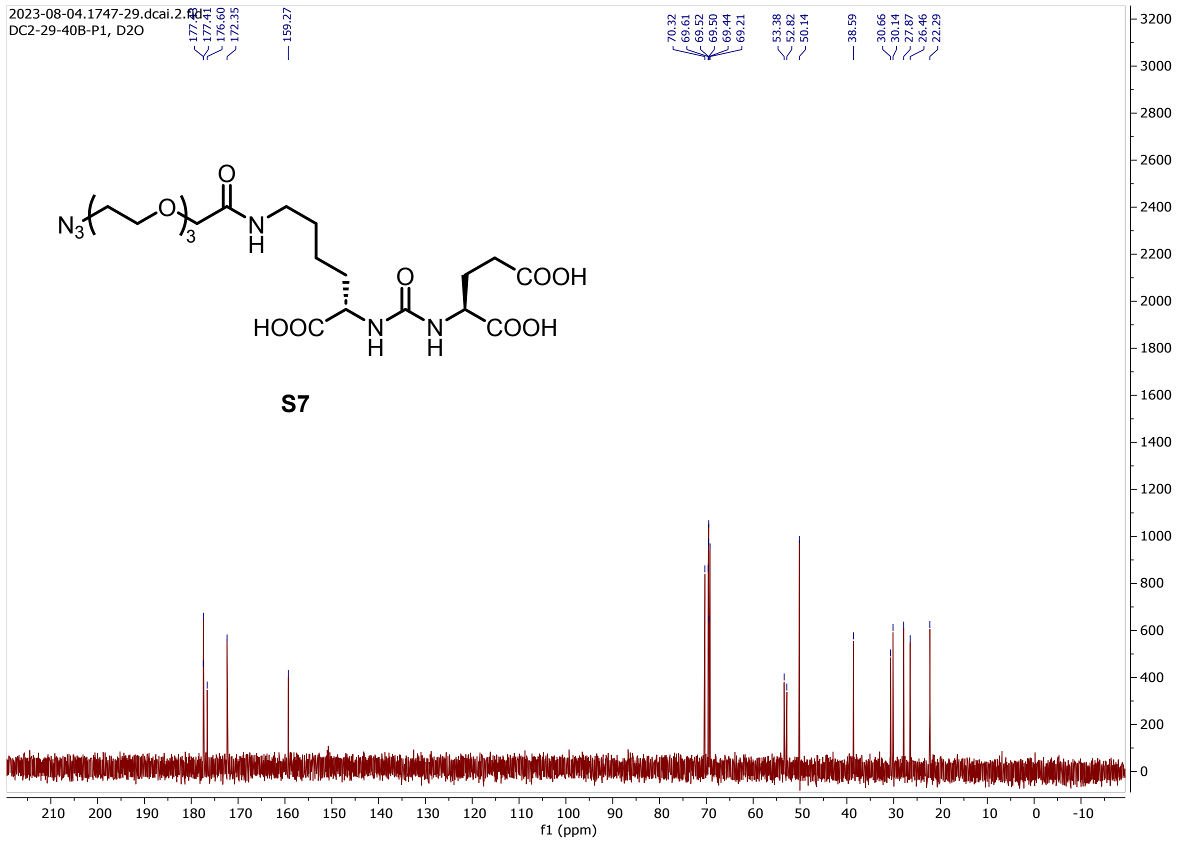
2023-08-04.1747-29.dcai.2.fid
DC2-29-40B-P1, D2O
S7
f1 (ppm)

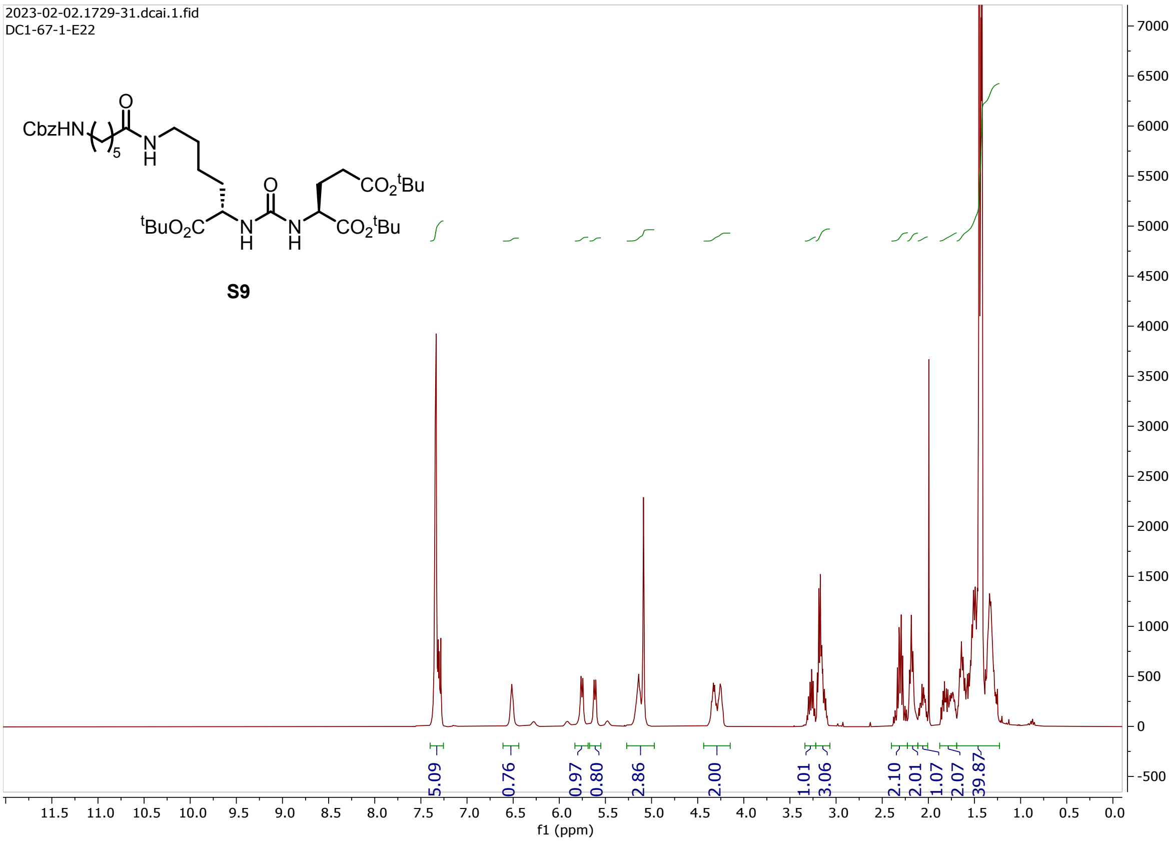
2023-02-02.1729-31.dcai.1.fid
DC1-67-1-E22
CbzHN
O
N
H
5
O
CO2tBu
tBuO2C
N
H
N
H
CO2tBu
S9
5.09
0.76
0.97
0.80
2.86
2.00
1.01
3.06
2.10
2.01
1.07
2.07
39.87
f1 (ppm)

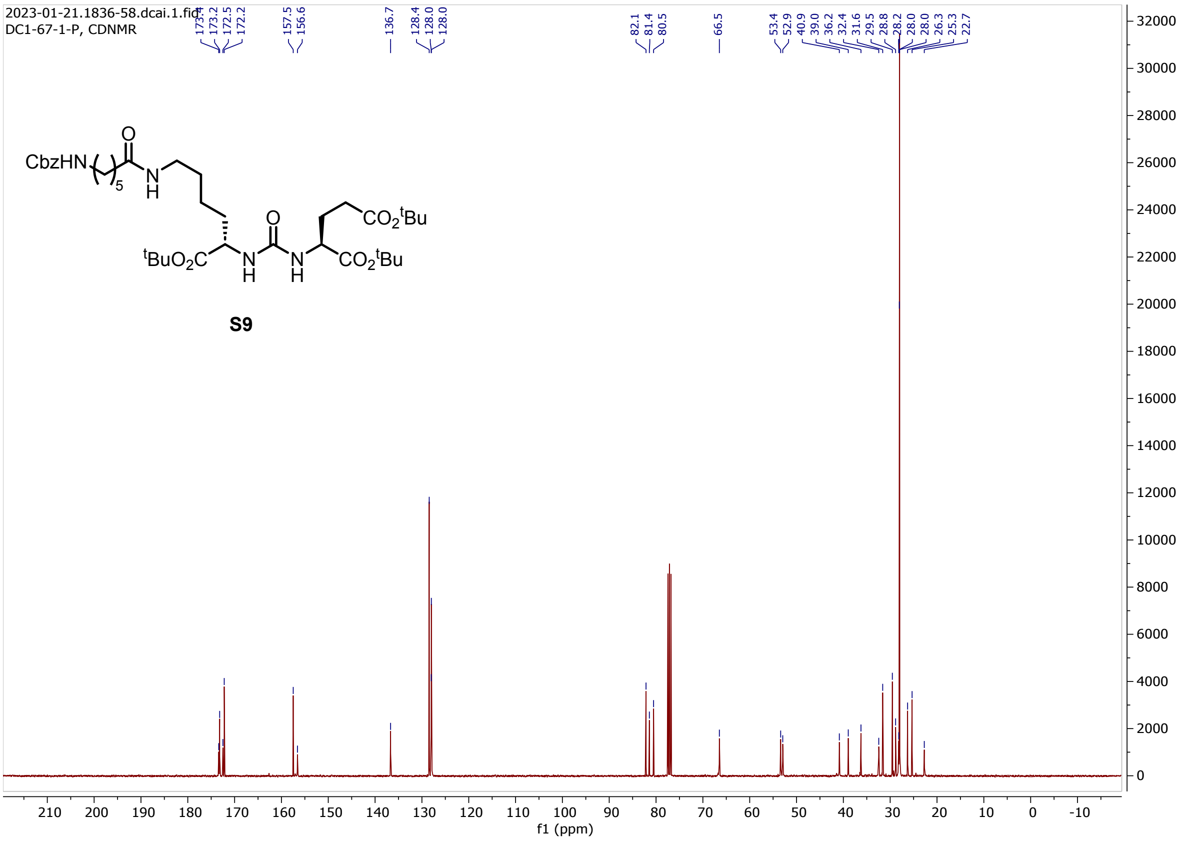
2023-01-21.1836-58.dcai.1.fid
DC1-67-1-P, CDNMR
173.2
172.5
172.2
157.5
156.6
136.7
128.4
128.0
128.0
82.1
81.4
80.5
66.5
53.4
52.9
40.9
39.0
36.2
32.4
31.6
29.5
28.8
28.2
28.0
28.0
26.3
25.3
22.7
CbzHN
O
N
H
5
O
CO2tBu
tBuO2C
N
H
N
H
CO2tBu
S9
f1 (ppm)

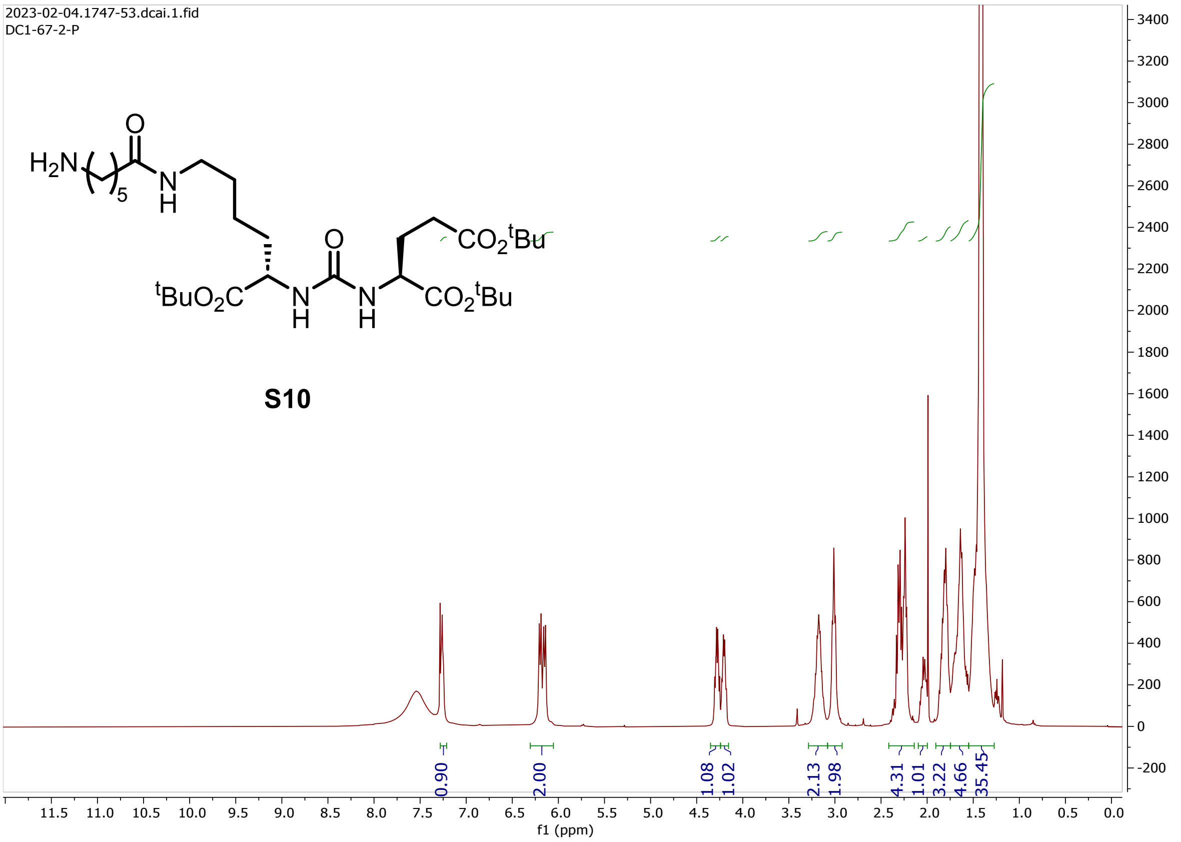
2023-02-04.1747-53.dcai.1.fid
DC1-67-2-P
S10
f1 (ppm)

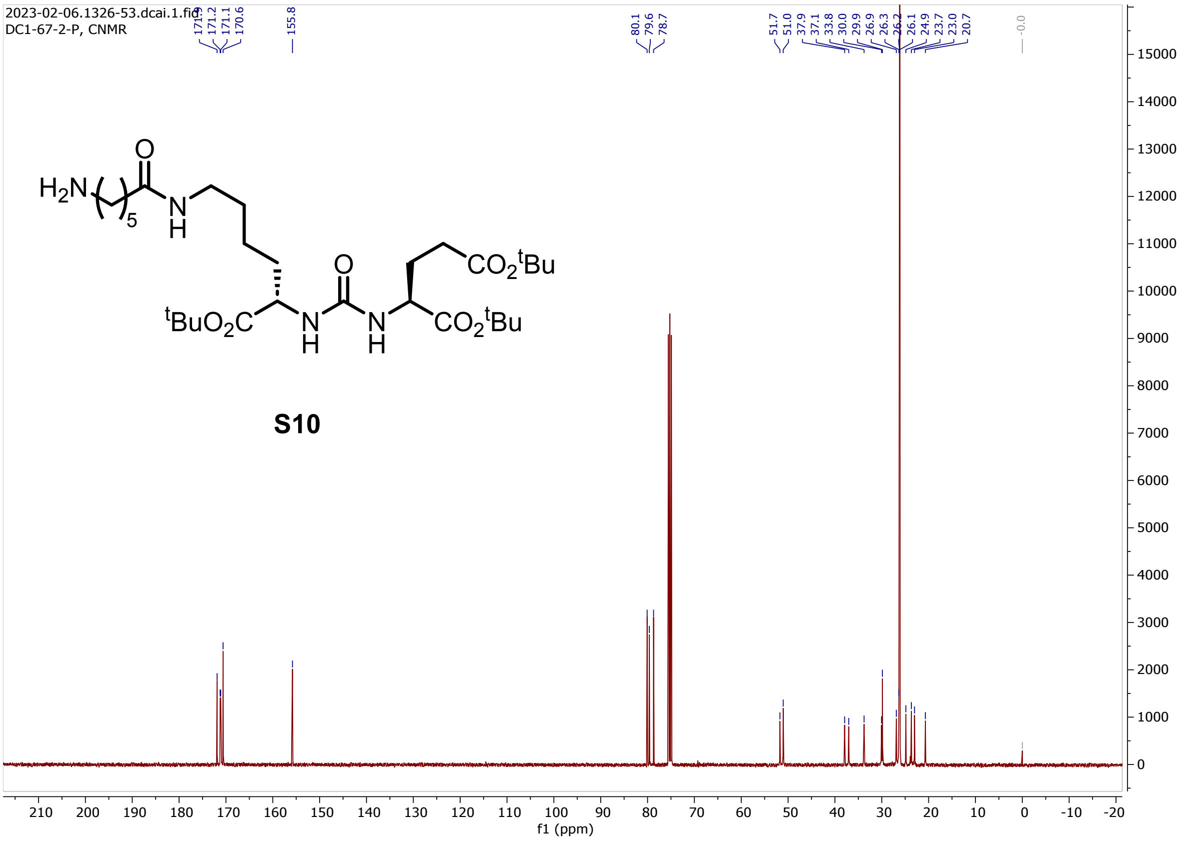
2023-02-06.1326-53.dcai.1.fid
DC1-67-2-P, CNMR
S10
f1 (ppm)

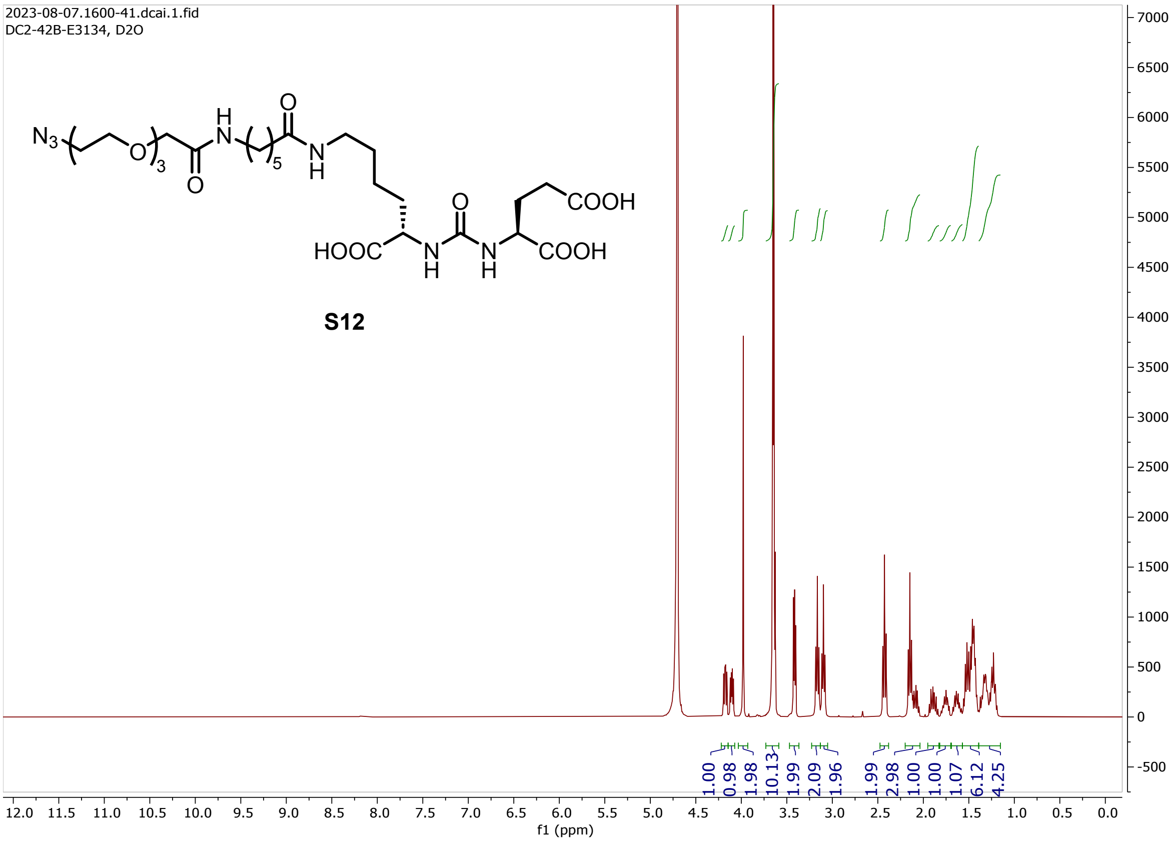

2023-08-07.1600-41.dcai.1.fid
DC2-42B-E3134, D2O
S12
1.00 0.98 1.98 10.13 1.99 2.09 1.96 1.99 2.98 1.00 1.00 1.07 6.12 4.25
f1 (ppm)

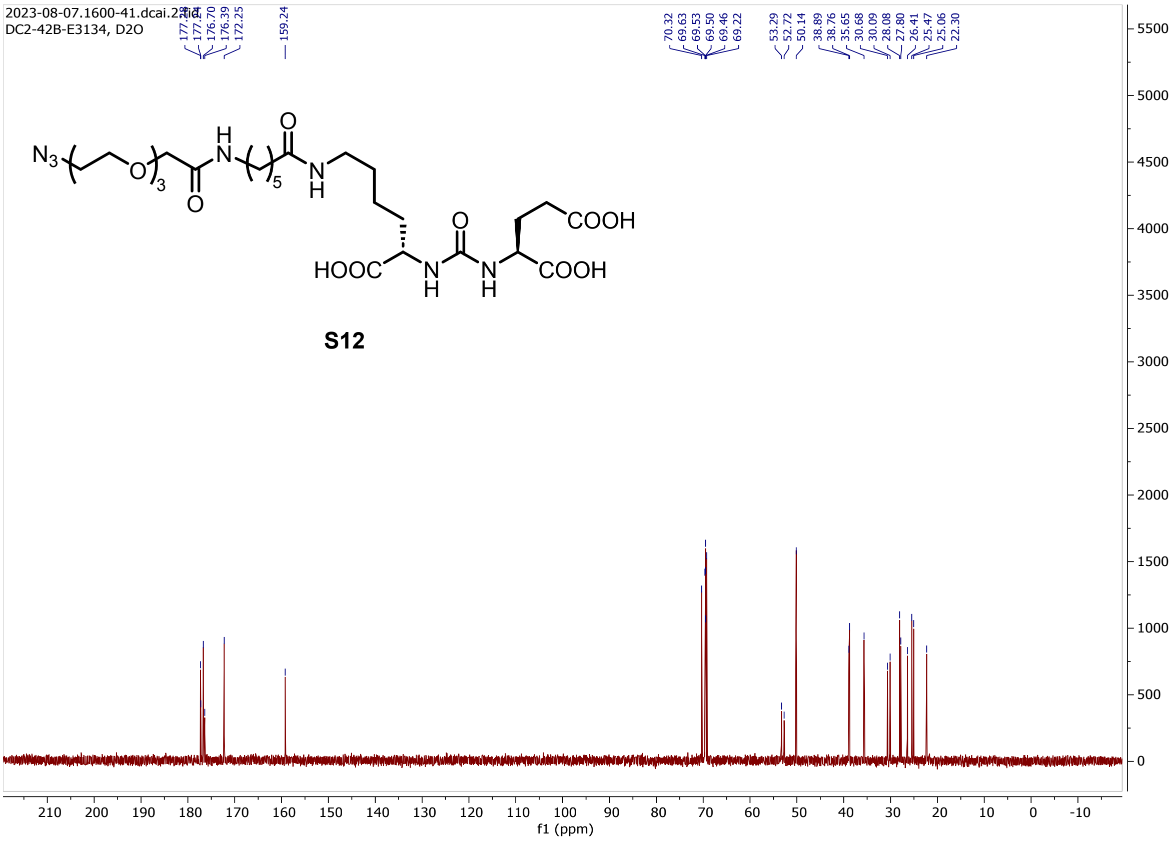

2023-08-07.1600-41.dcai.2.fid
DC2-42B-E3134, D2O
177.70 176.70 176.39 172.25 159.24
70.32 69.63 69.53 69.50 69.46 69.22
53.29 52.72 50.14
38.89 38.76 35.65 30.68 30.09 28.08 27.80 26.41 25.47 25.06 22.30
S12
f1 (ppm)

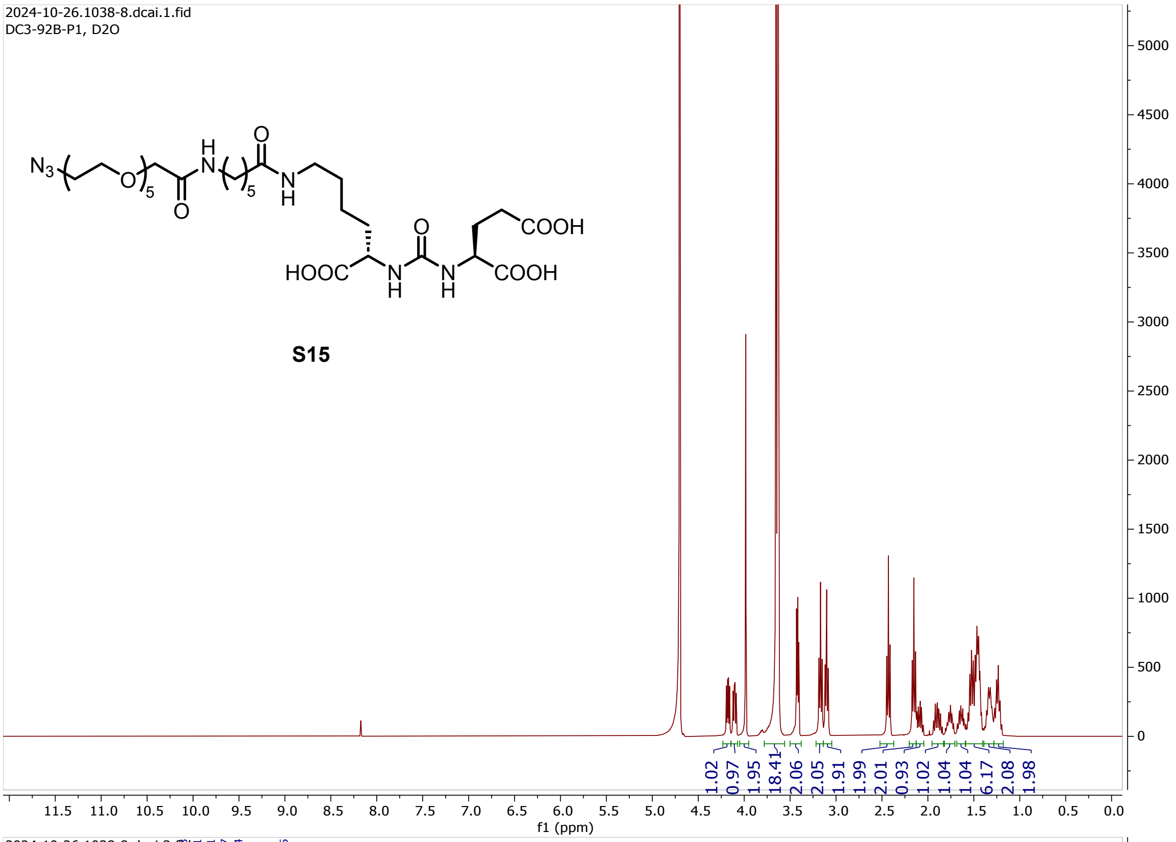
2024-10-26.1038-8.dcai.1.fid
DC3-92B-P1, D2O
S15
1.02
0.97
1.95
18.41
2.06
2.05
1.91
1.99
2.01
0.93
1.02
1.04
1.04
6.17
2.08
1.98
f1 (ppm)

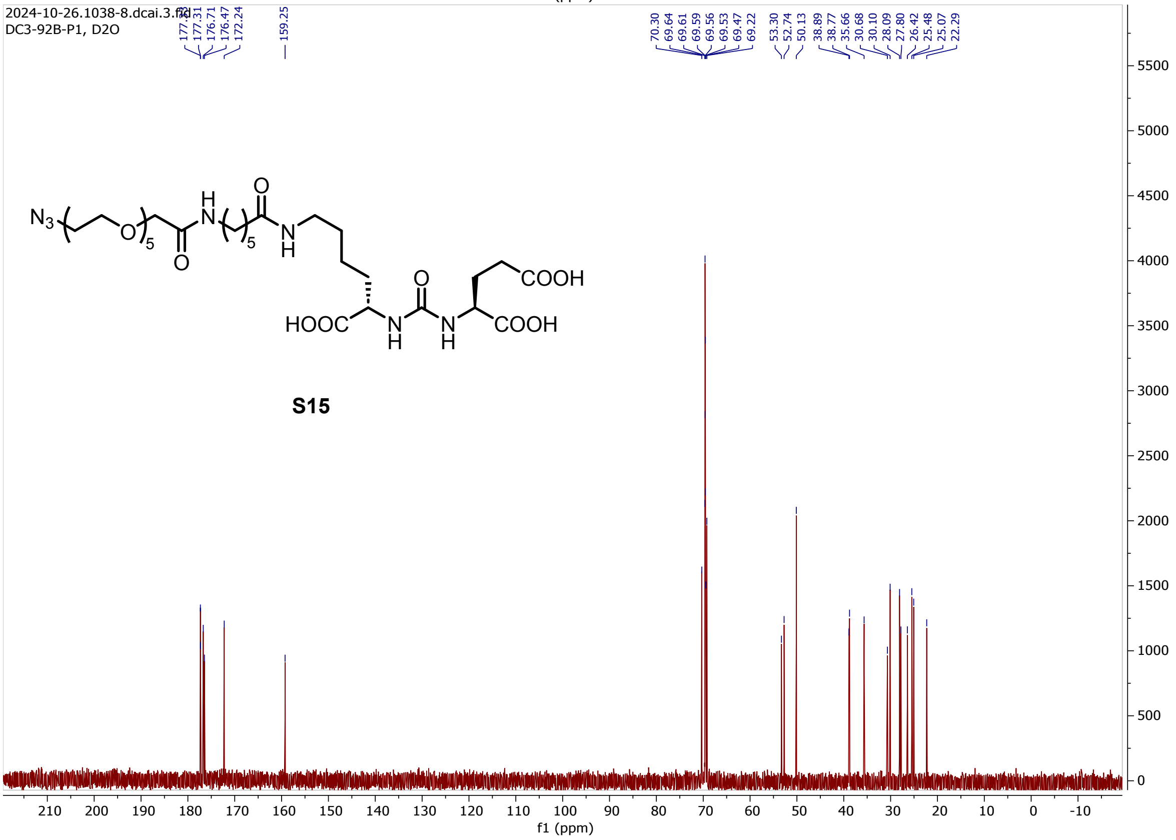
2024-10-26.1038-8.dcai.3.fid
DC3-92B-P1, D2O
177.31
176.71
176.47
172.24
159.25
70.30
69.64
69.61
69.59
69.56
69.53
69.47
69.22
53.30
52.74
50.13
38.89
38.77
35.66
30.68
30.10
28.09
27.80
26.42
25.48
25.07
22.29
S15
f1 (ppm)

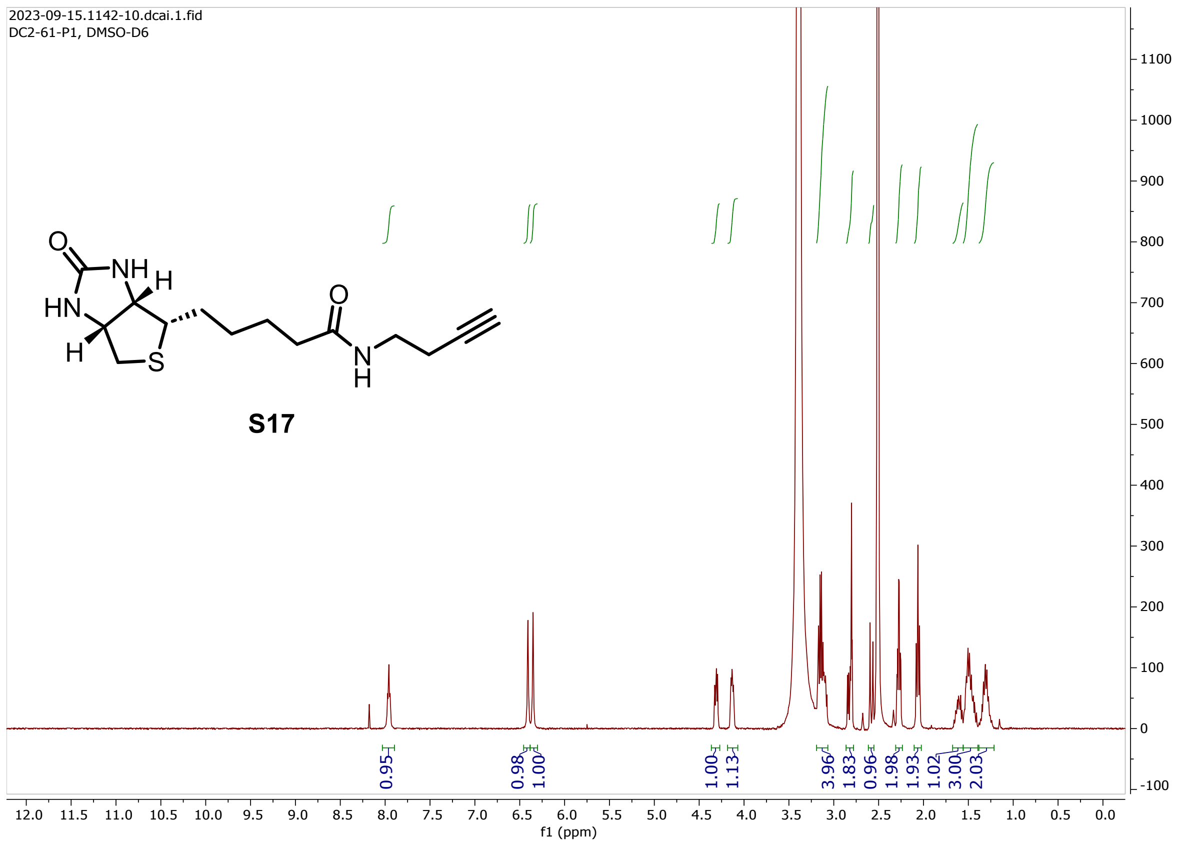

2023-09-15.1142-10.dcai.1.fid
DC2-61-P1, DMSO-D6
S17
0.95
0.98
1.00
1.00
1.13
3.96
1.83
0.96
1.98
1.93
1.02
3.00
2.03
f1 (ppm)

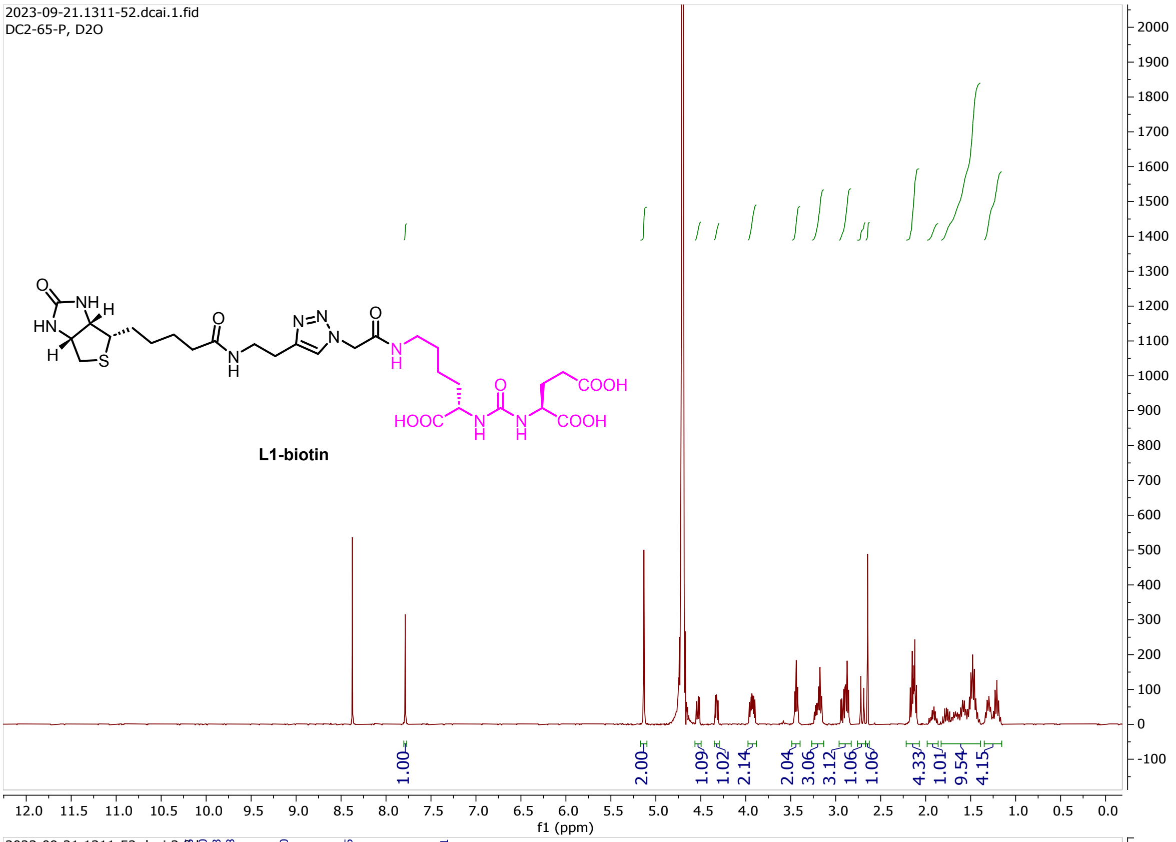
2023-09-21.1311-52.dcai.1.fid
DC2-65-P, D2O
L1-biotin
1.00
2.00
1.09
1.02
2.14
2.04
3.06
3.12
1.06
1.06
4.33
1.01
9.54
4.15
f1 (ppm)

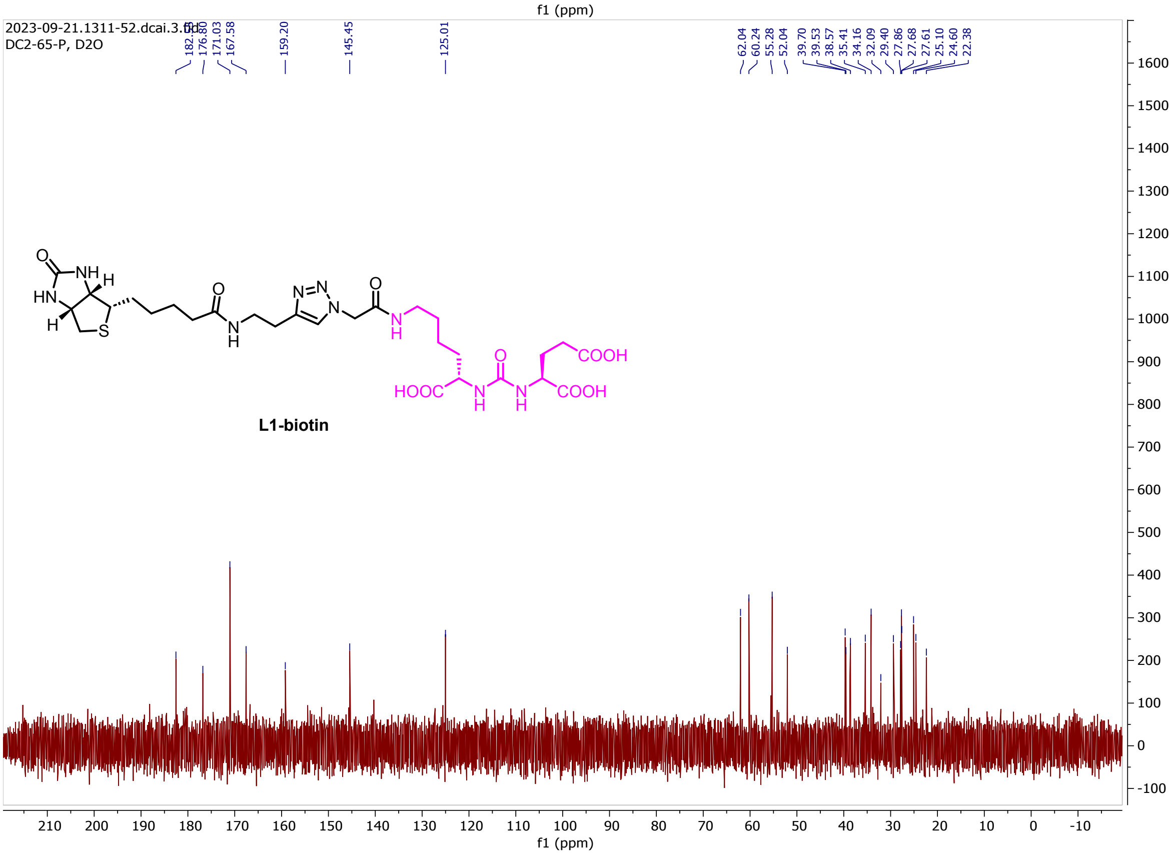
2023-09-21.1311-52.dcai.3.fid
DC2-65-P, D2O
176.80
171.03
167.58
159.20
145.45
125.01
62.04
60.24
55.28
52.04
39.70
39.53
38.57
35.41
34.16
32.09
29.40
27.86
27.68
27.61
25.10
24.60
22.38
L1-biotin
f1 (ppm)

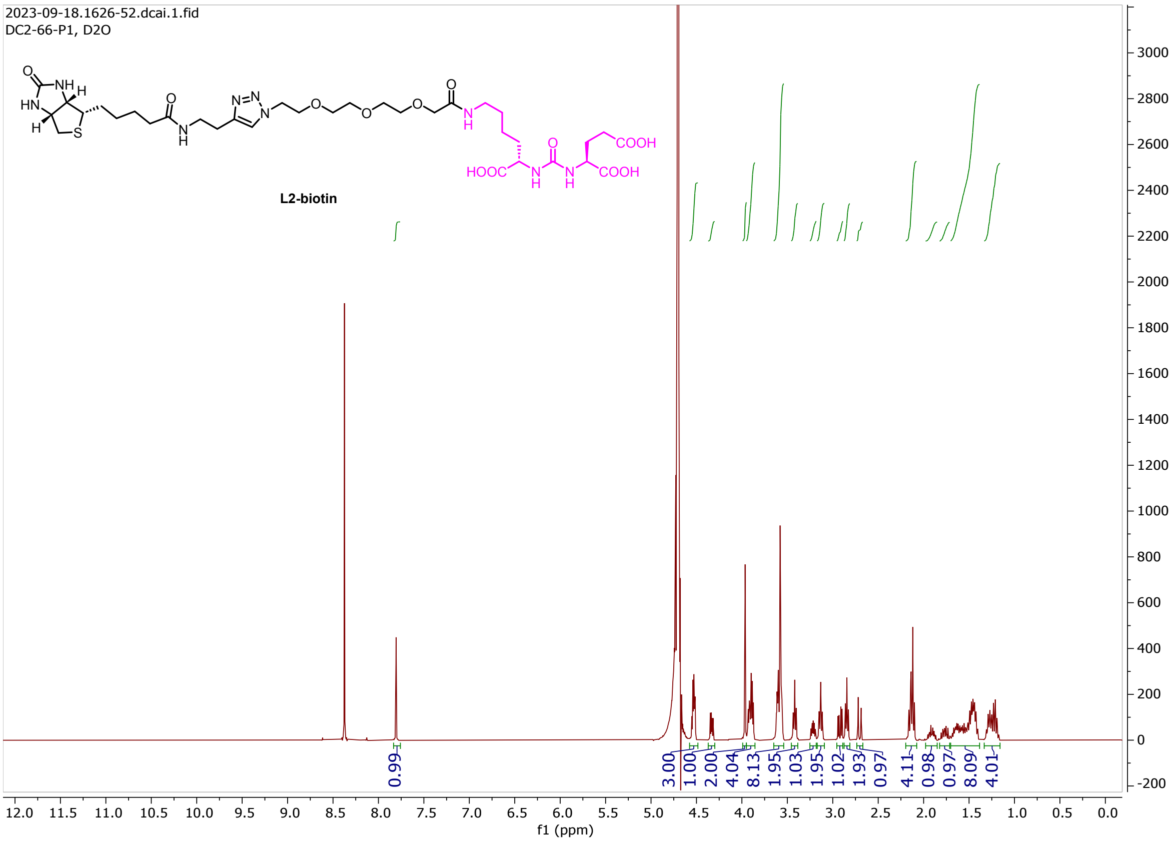
2023-09-18.1626-52.dcai.1.fid
DC2-66-P1, D2O
L2-biotin
0.99
3.00
1.00
2.00
4.04
8.13
1.95
1.03
1.95
1.02
1.93
0.97
4.11
0.98
0.97
8.09
4.01
f1 (ppm)

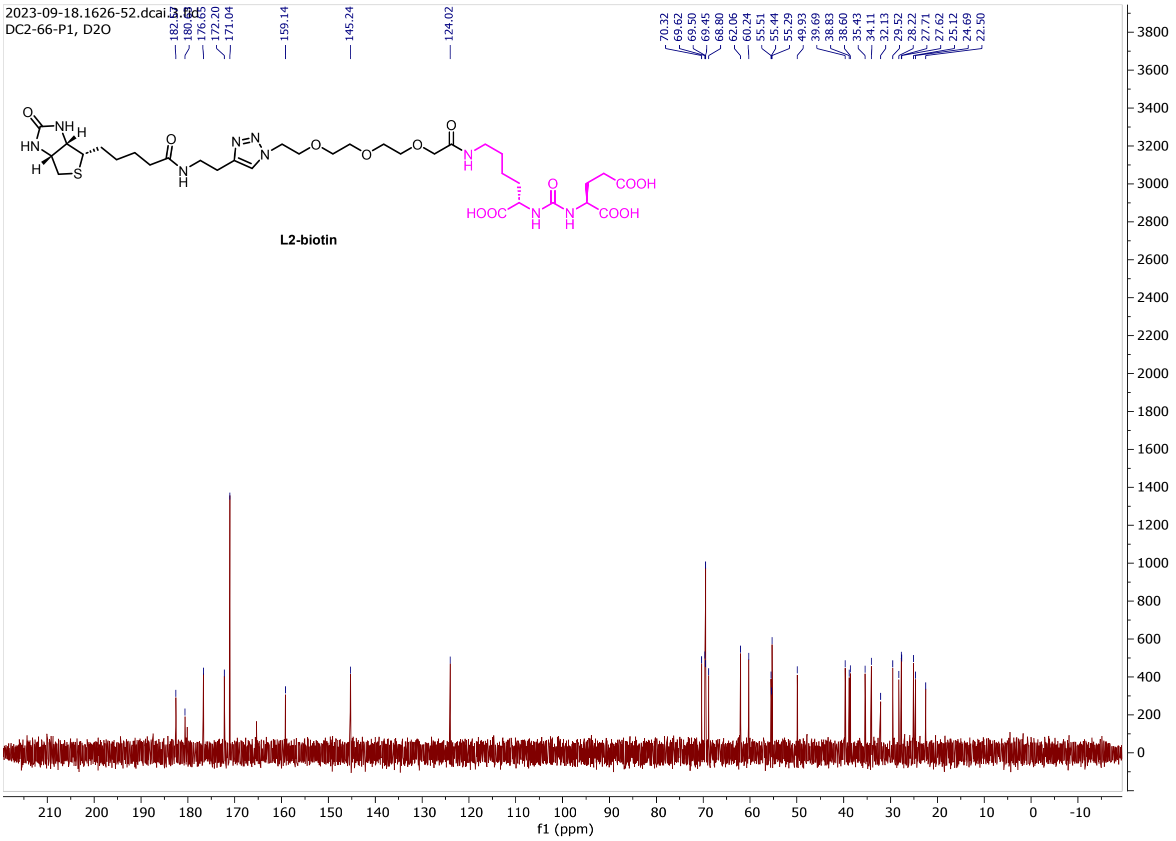
DC2-66-P1, D2O
172.20
171.04
159.14
145.24
124.02
70.32
69.62
69.50
69.45
68.80
62.06
60.24
55.51
55.44
55.29
49.93
39.69
38.83
38.60
35.43
34.11
32.13
29.52
28.22
27.71
27.62
25.12
24.69
22.50
L2-biotin
f1 (ppm)

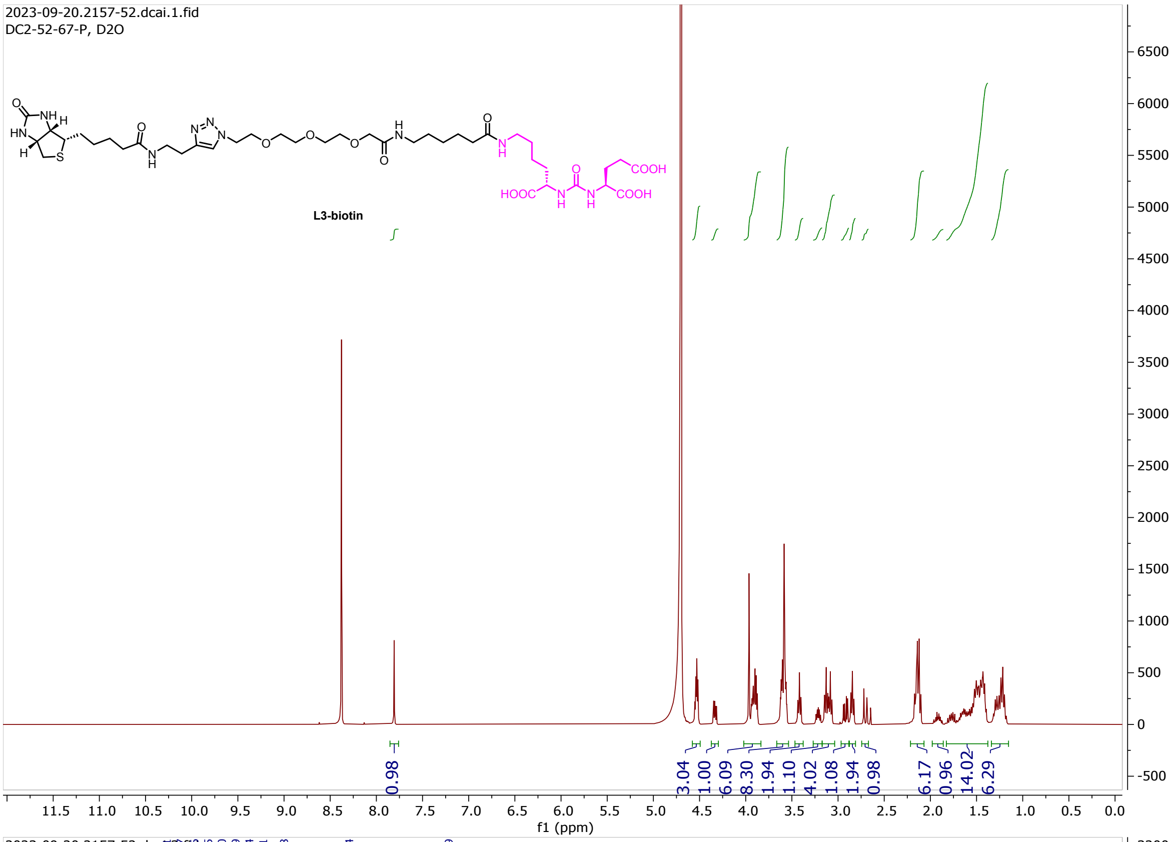
2023-09-20.2157-52.dcai.1.fid
DC2-52-67-P, D2O
L3-biotin
0.98
3.04
1.00
6.09
8.30
1.94
1.10
4.02
1.08
1.94
0.98
6.17
0.96
14.02
6.29
f1 (ppm)

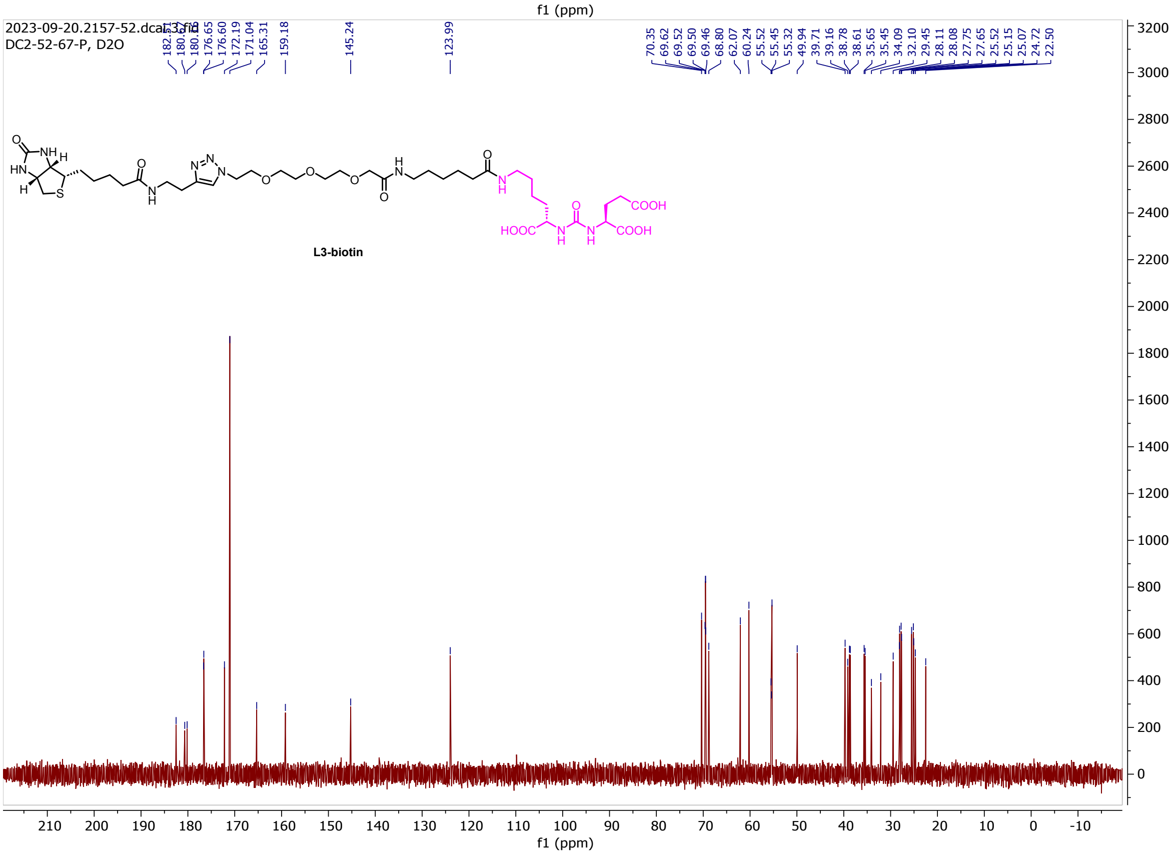
DC2-52-67-P, D2O
176.65
176.60
172.19
171.04
165.31
159.18
145.24
123.99
70.35
69.62
69.52
69.50
69.46
68.80
62.07
60.24
55.52
55.45
55.32
49.94
39.71
39.16
38.78
38.61
35.65
35.45
34.09
32.10
29.45
28.11
28.08
27.75
27.65
25.52
25.15
25.07
24.72
22.50
L3-biotin
f1 (ppm)

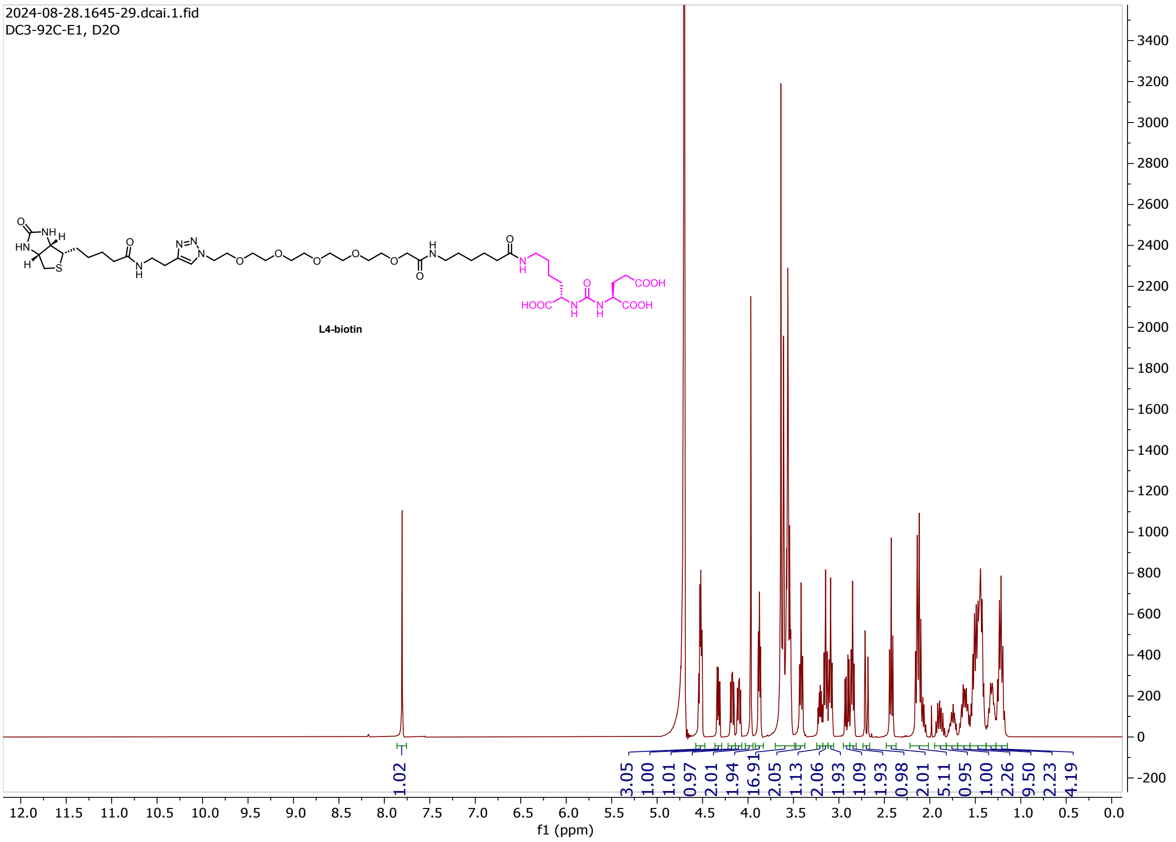

2024-08-28.1645-29.dcai.1.fid
DC3-92C-E1, D2O
L4-biotin
f1 (ppm)

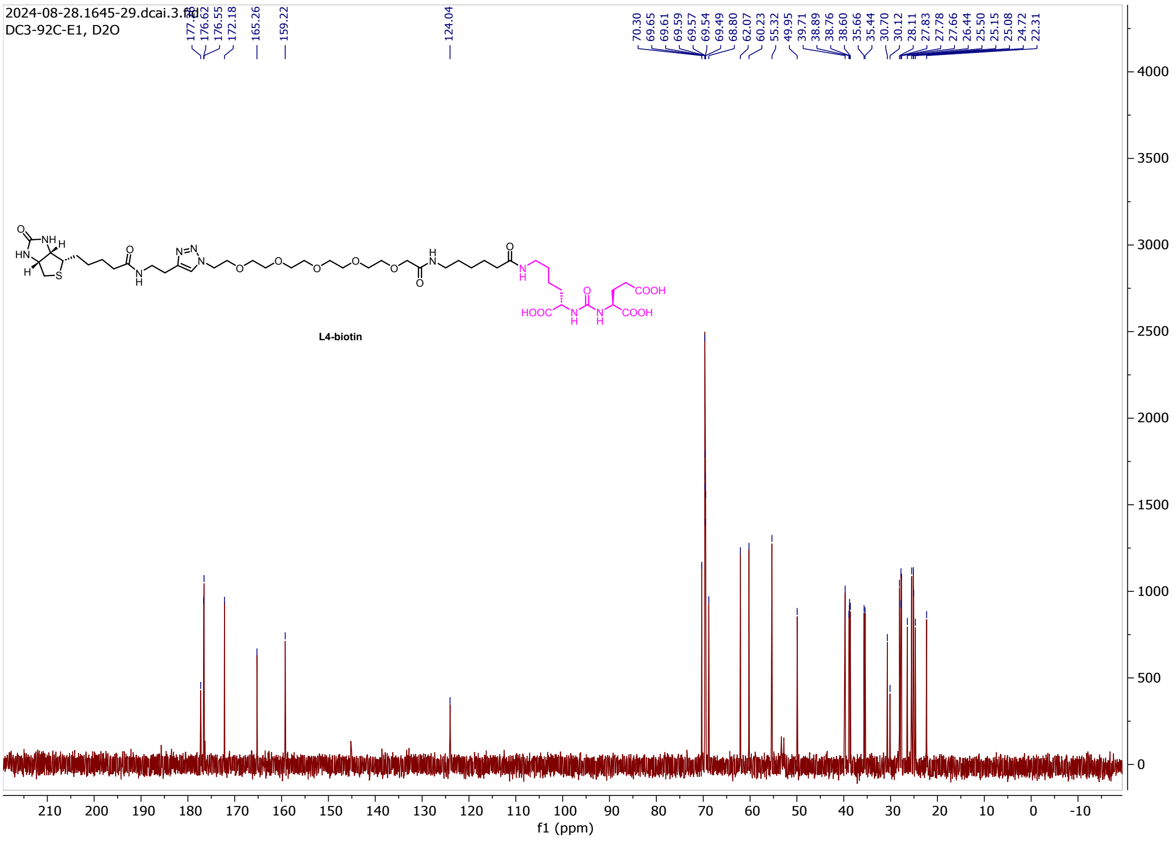

2024-08-28.1645-29.dcai.3.fid
DC3-92C-E1, D2O
L4-biotin
f1 (ppm)

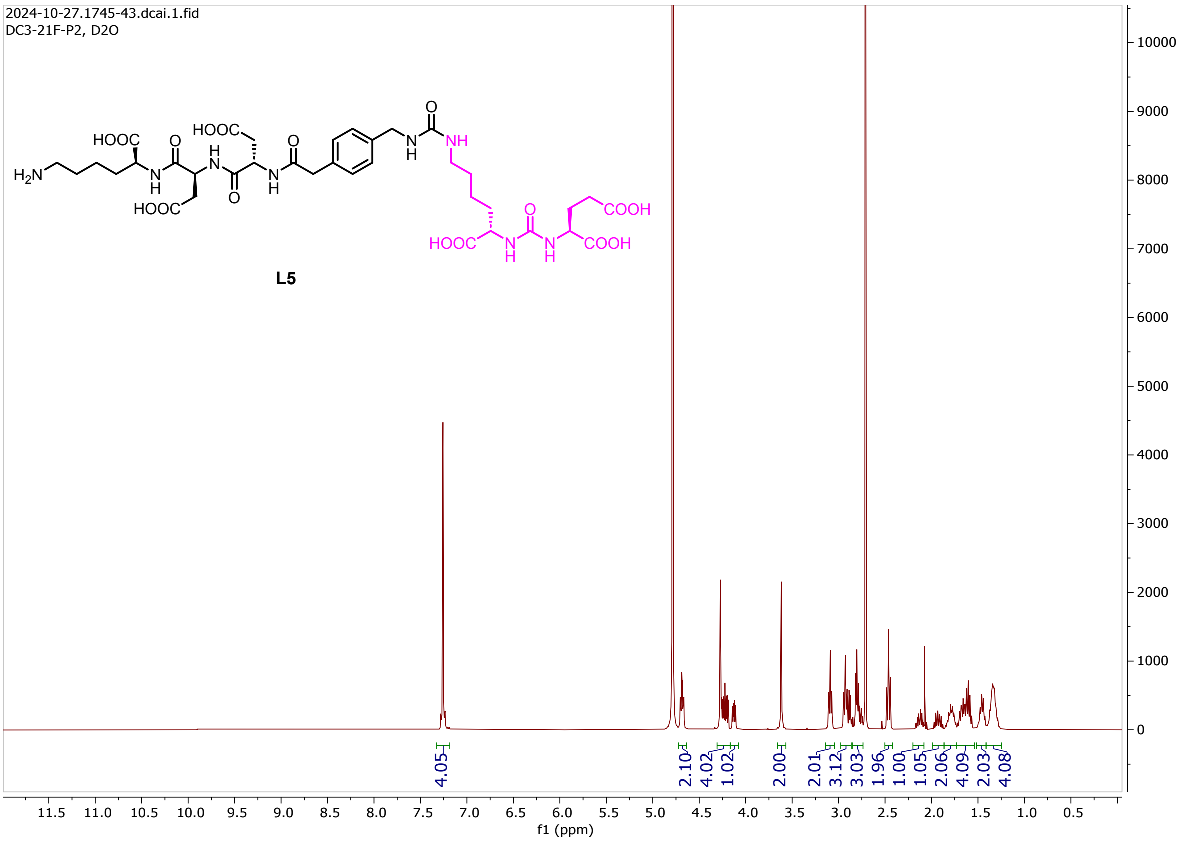
2024-10-27.1745-43.dcai.1.fid
DC3-21F-P2, D2O
L5
f1 (ppm)

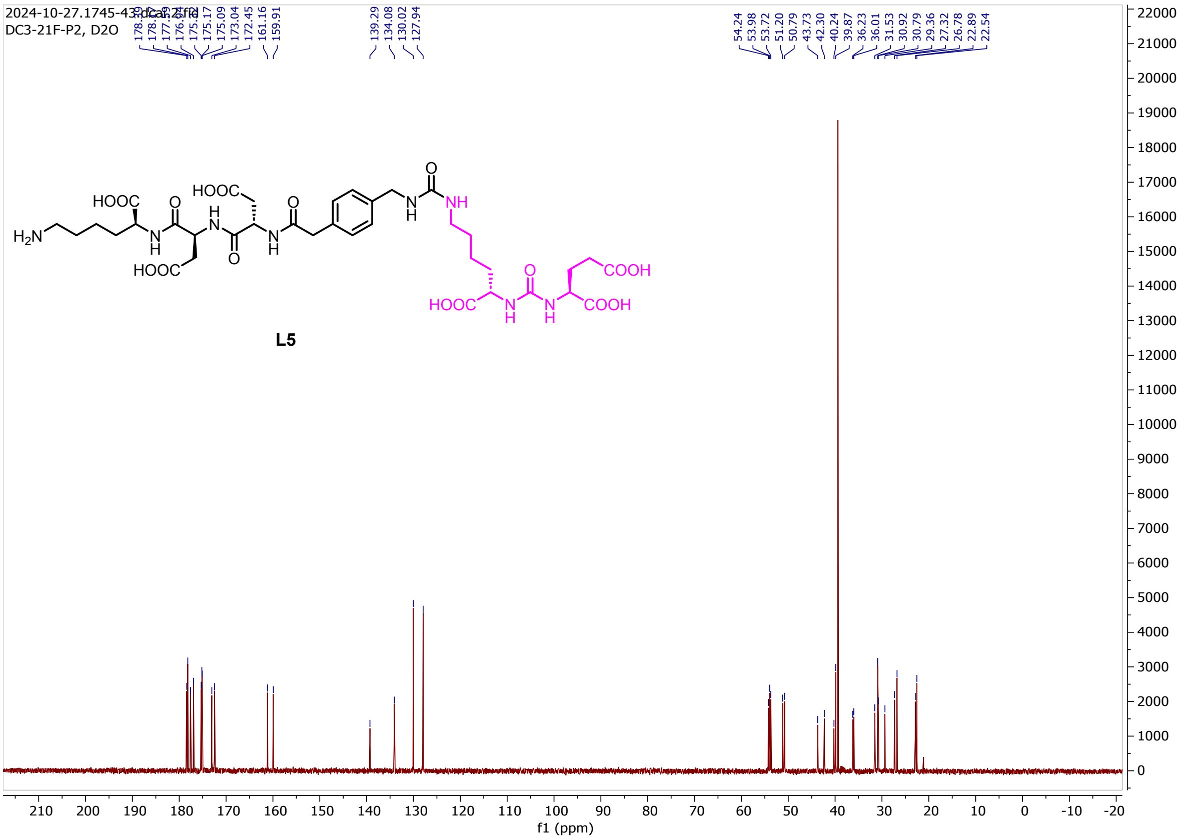
DC3-21F-P2, D2O
L5
f1 (ppm)

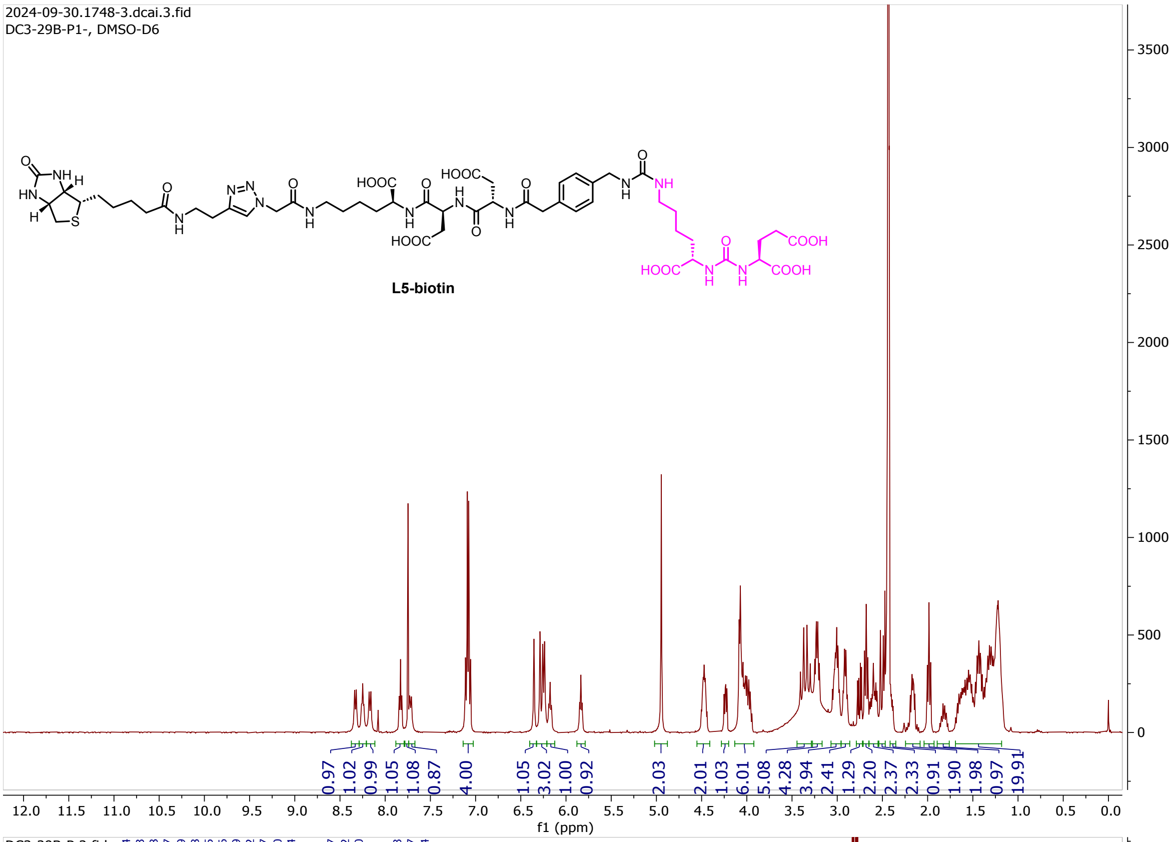
2024-09-30.1748-3.dcai.3.fid
DC3-29B-P1-, DMSO-D6
L5-biotin
0.97 1.02 0.99 1.05 1.08 0.87 4.00 1.05 3.02 1.00 0.92 2.03 2.01 1.03 6.01 5.08 4.28 3.94 2.41 1.29 2.20 2.37 2.33 0.91 1.90 1.98 0.97 19.91
f1 (ppm)

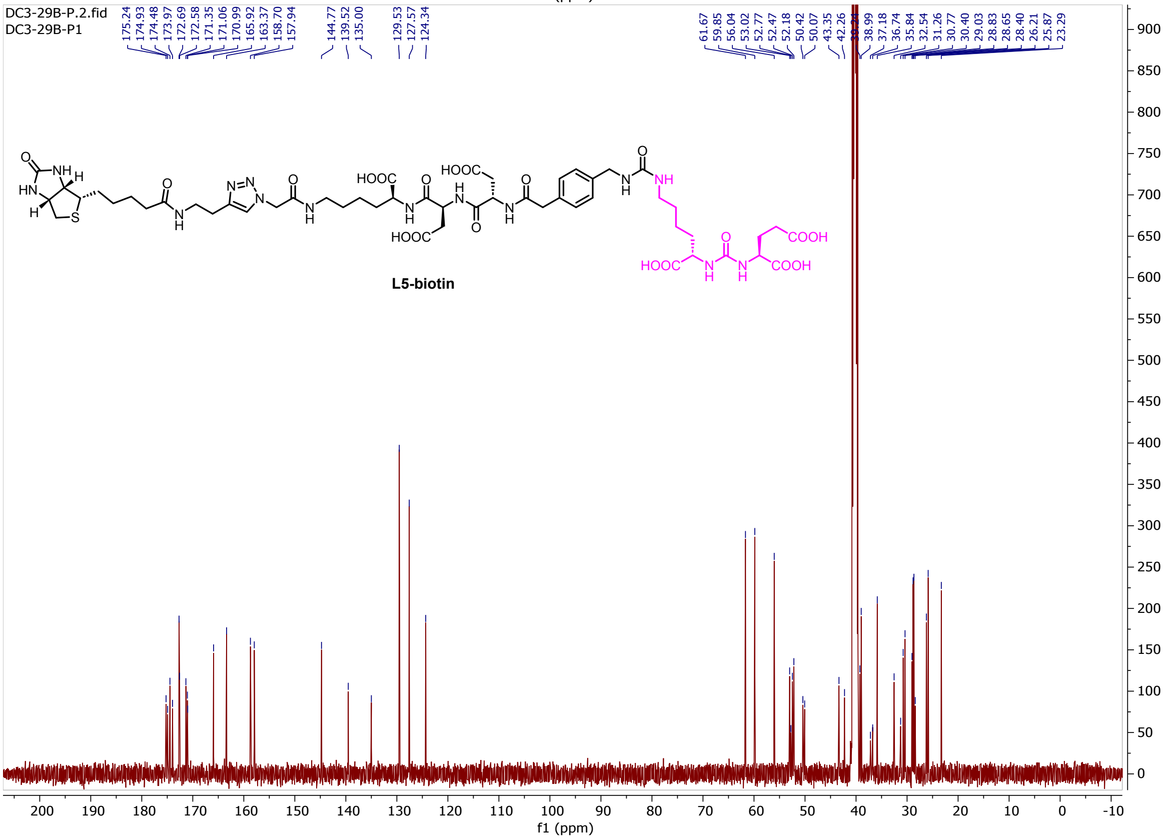
DC3-29B-P.2.fid
DC3-29B-P1
175.24 174.93 174.48 173.97 172.69 172.58 171.35 171.06 170.99 165.92 163.37 158.70 157.94
144.77 139.52 135.00
129.53 127.57 124.34
61.67 59.85 56.04 53.02 52.77 52.47 52.18 50.42 50.07 43.35 42.26
38.99 37.18 36.74 35.84 32.54 31.26 30.77 30.40 29.03 28.83 28.65 28.40 26.21 25.87 23.29
L5-biotin
f1 (ppm)

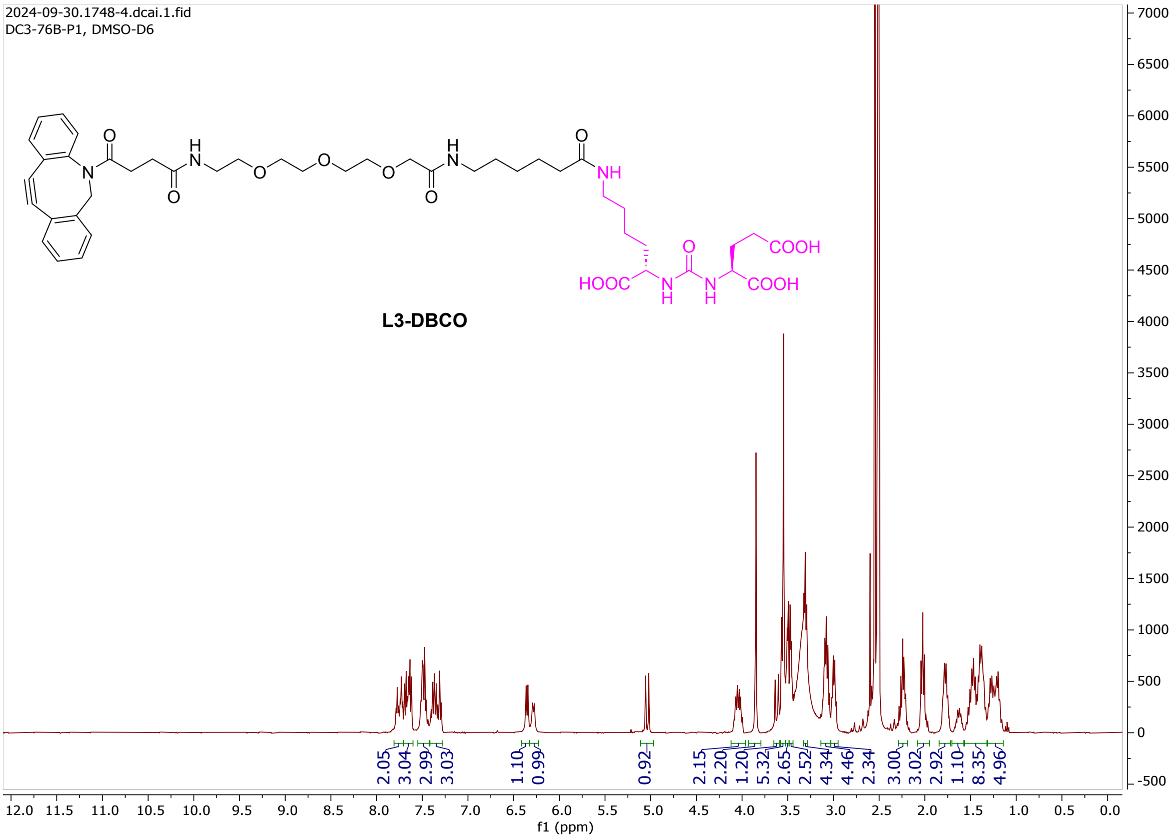
2024-09-30.1748-4.dcai.1.fid
DC3-76B-P1, DMSO-D6
L3-DBCO
HOOC
COOH
COOH
2.05 3.04 2.99 3.03 1.10 0.99 0.92 2.15 2.20 1.20 5.32 2.65 2.52 4.34 4.46 2.34 3.00 3.02 2.92 1.10 8.35 4.96
f1 (ppm)

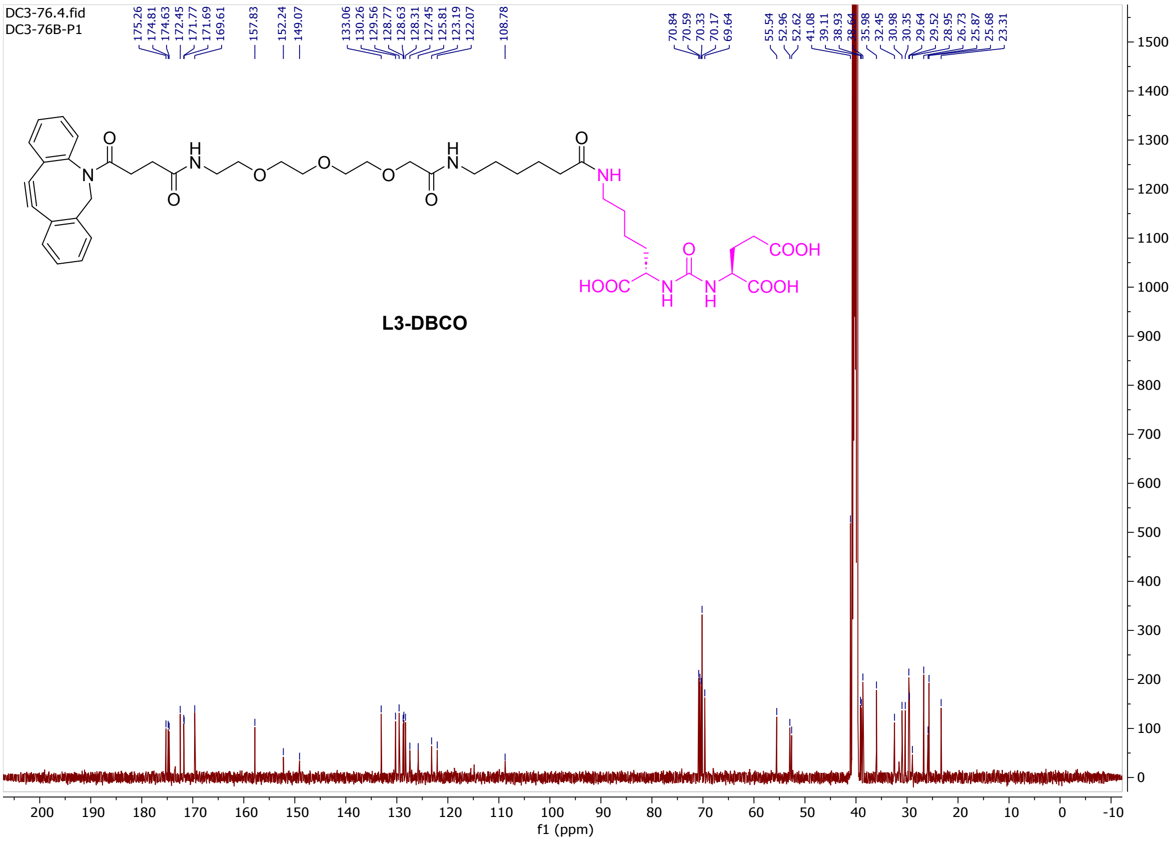
DC3-76.4.fid
DC3-76B-P1
175.26 174.81 174.63 172.45 171.77 171.69 169.61 157.83 152.24 149.07 133.06 130.26 129.56 128.77 128.63 128.31 127.45 125.81 123.19 122.07 108.78 70.84 70.59 70.33 70.17 69.64 55.54 52.96 52.62 41.08 39.11 38.93 35.98 32.45 30.98 30.35 29.64 29.52 28.95 26.73 25.87 25.68 23.31
L3-DBCO
HOOC
COOH
COOH
f1 (ppm)

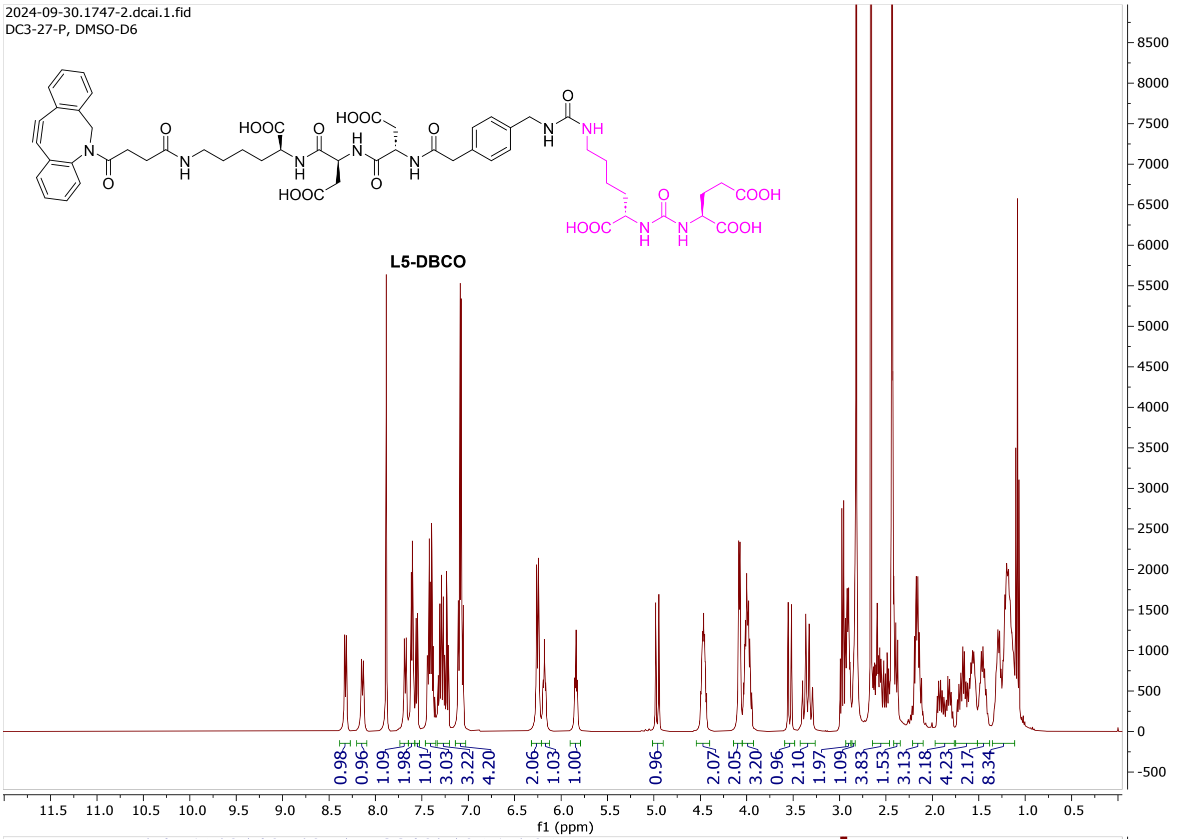
2024-09-30.1747-2.dcai.1.fid
DC3-27-P, DMSO-D6
L5-DBCO
f1 (ppm)

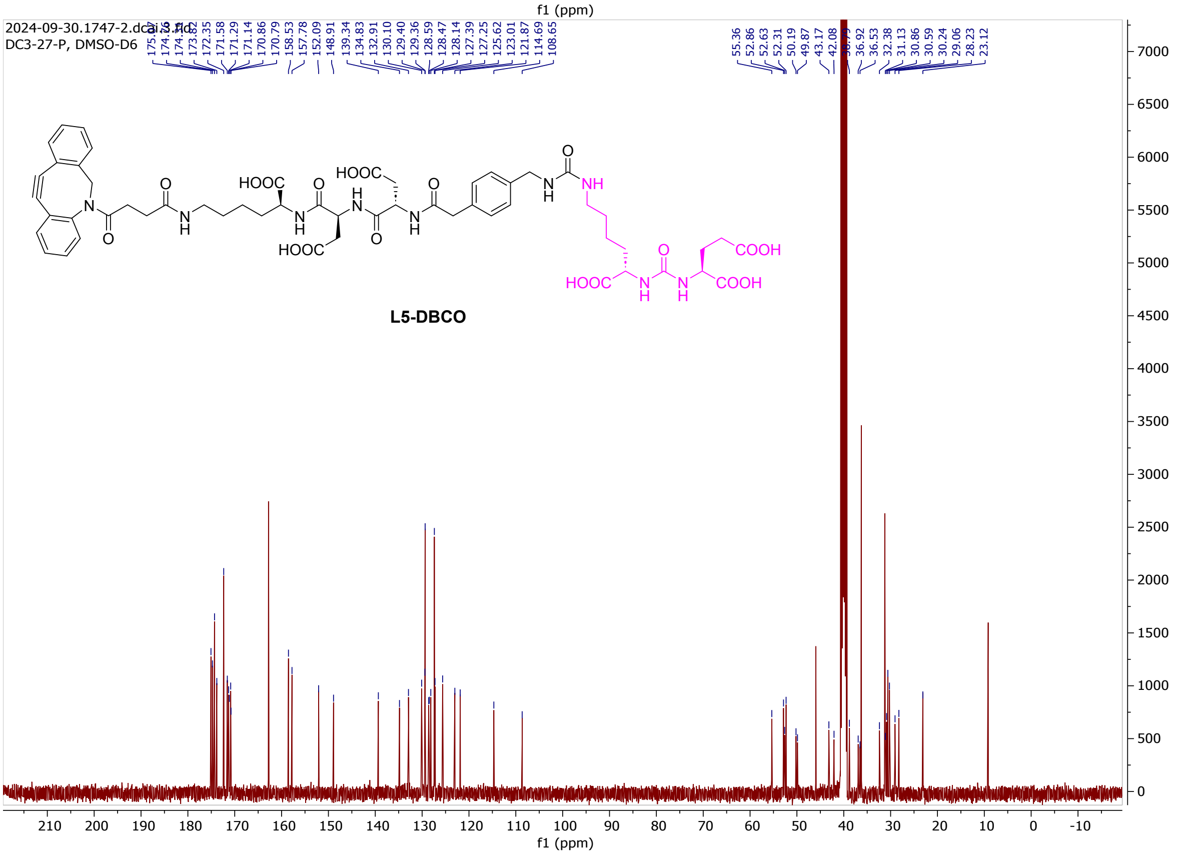
DC3-27-P, DMSO-D6
L5-DBCO
f1 (ppm)

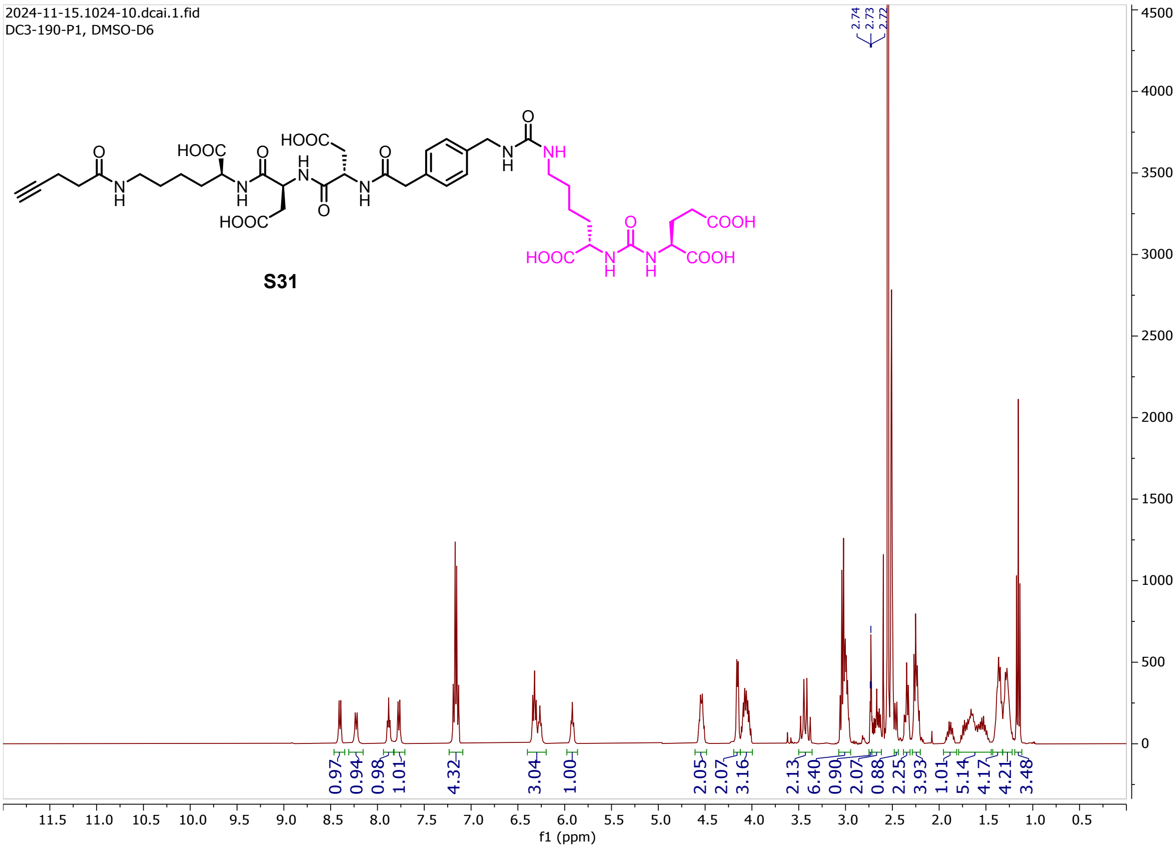

2024-11-15.1024-10.dcai.1.fid
DC3-190-P1, DMSO-D6
2.74
2.73
2.72
HOOC
HOOC
HOOC
NH
COOH
HOOC
COOH
S31
0.97
0.94
0.98
1.01
4.32
3.04
1.00
2.05
2.07
3.16
2.13
6.40
0.90
2.07
0.88
2.25
3.93
1.01
5.14
4.17
4.21
3.48
f1 (ppm)

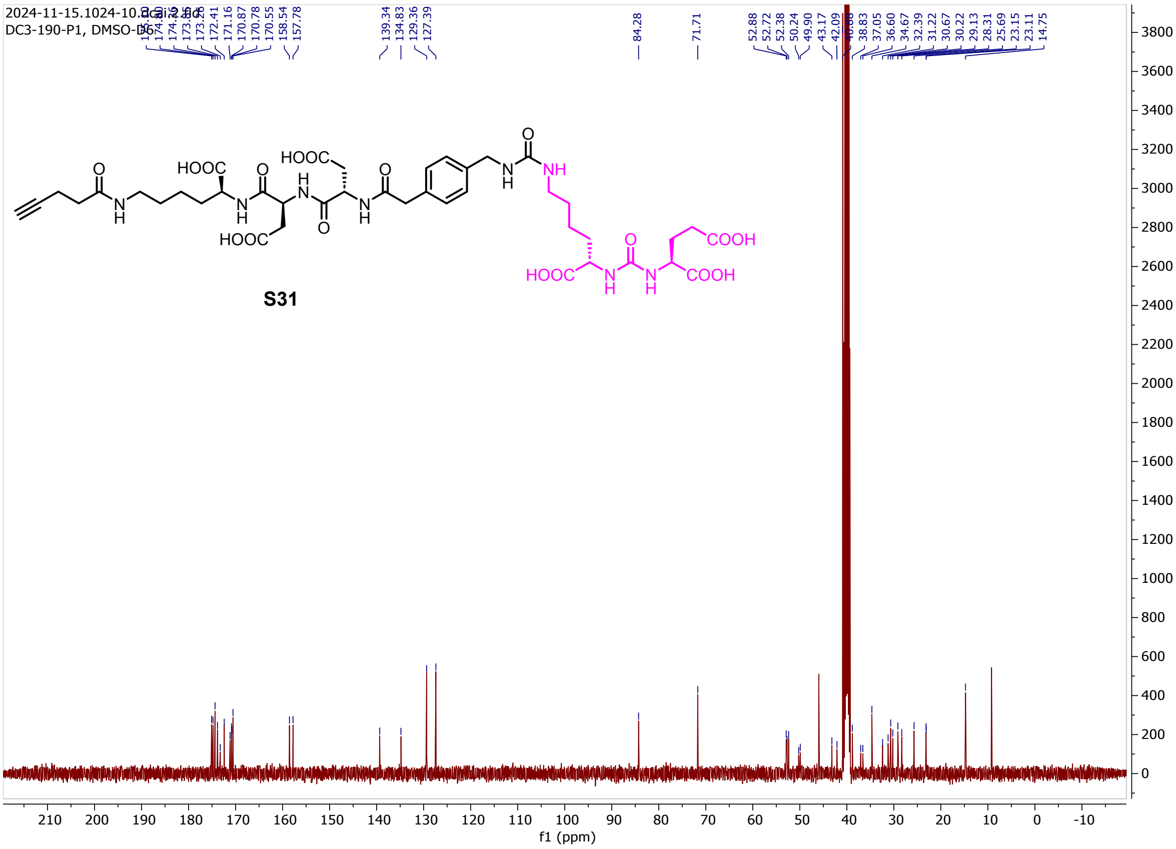

2024-11-15.1024-10.dcai.2.fid
DC3-190-P1, DMSO-D6
172.41
171.16
170.87
170.78
170.55
158.54
157.78
139.34
134.83
129.36
127.39
84.28
71.71
52.88
52.72
52.38
50.24
49.90
43.17
42.09
38.83
37.05
36.60
34.67
32.39
31.22
30.67
30.22
29.13
28.31
25.69
23.15
23.11
14.75
HOOC
HOOC
HOOC
NH
COOH
HOOC
COOH
S31
f1 (ppm)

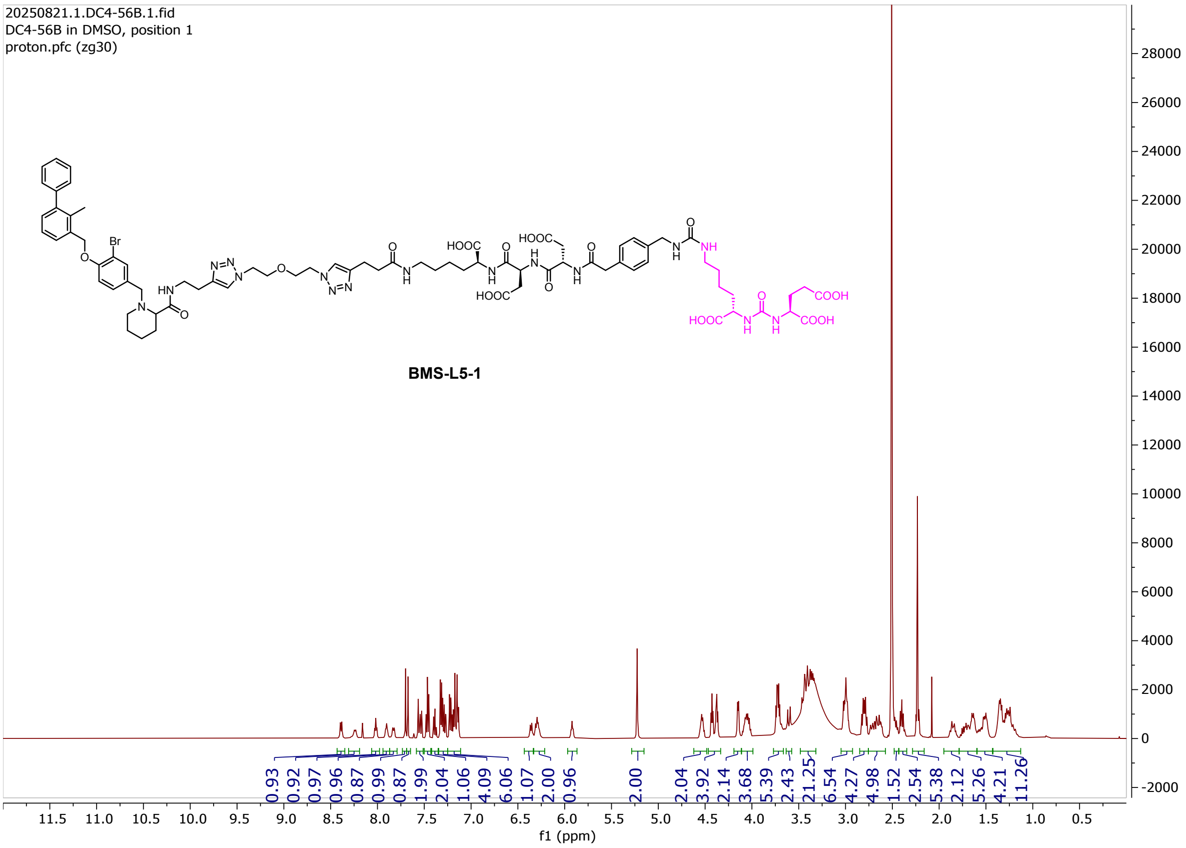

20250821.1.DC4-56B.1.fid
DC4-56B in DMSO, position 1
proton.pfc (zg30)
BMS-L5-1
0.93
0.92
0.97
0.96
0.87
0.99
0.87
1.99
2.04
1.06
4.09
6.06
1.07
2.00
0.96
2.00
2.04
3.92
2.14
3.68
5.39
2.43
21.25
6.54
4.27
4.98
1.52
2.54
5.38
2.12
5.26
4.21
11.26
f1 (ppm)

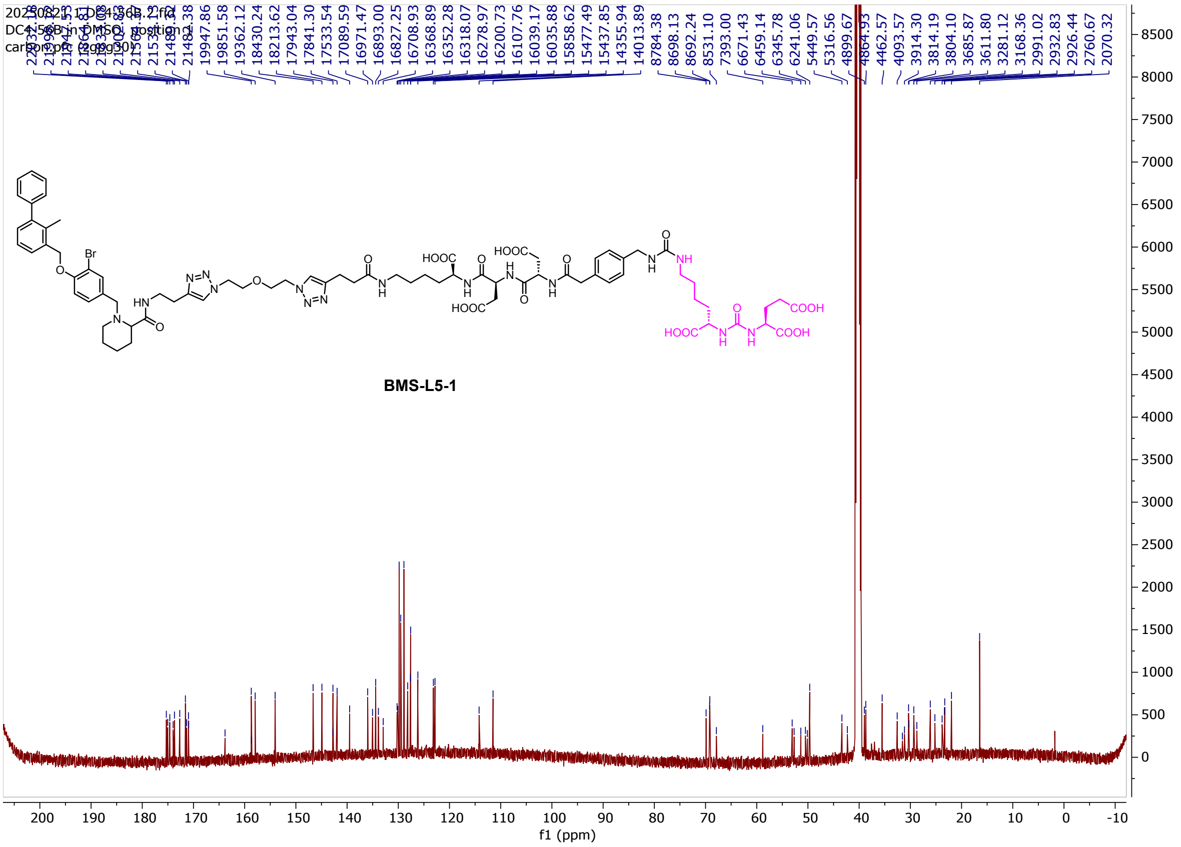

19947.86
19851.58
19362.12
18430.24
18213.62
17943.04
17841.30
17533.54
17089.59
16971.47
16893.00
16827.25
16708.93
16368.89
16352.28
16318.07
16278.97
16200.73
16107.76
16039.17
16035.88
15858.62
15477.49
15437.85
14355.94
14013.89
8784.38
8698.13
8692.24
8531.10
7393.00
6671.43
6459.14
6345.78
6241.06
5449.57
5316.56
4899.67
4864.93
4462.57
4093.57
3914.30
3814.19
3804.10
3685.87
3611.80
3281.12
3168.36
2991.02
2932.83
2926.44
2760.67
2070.32
BMS-L5-1
f1 (ppm)

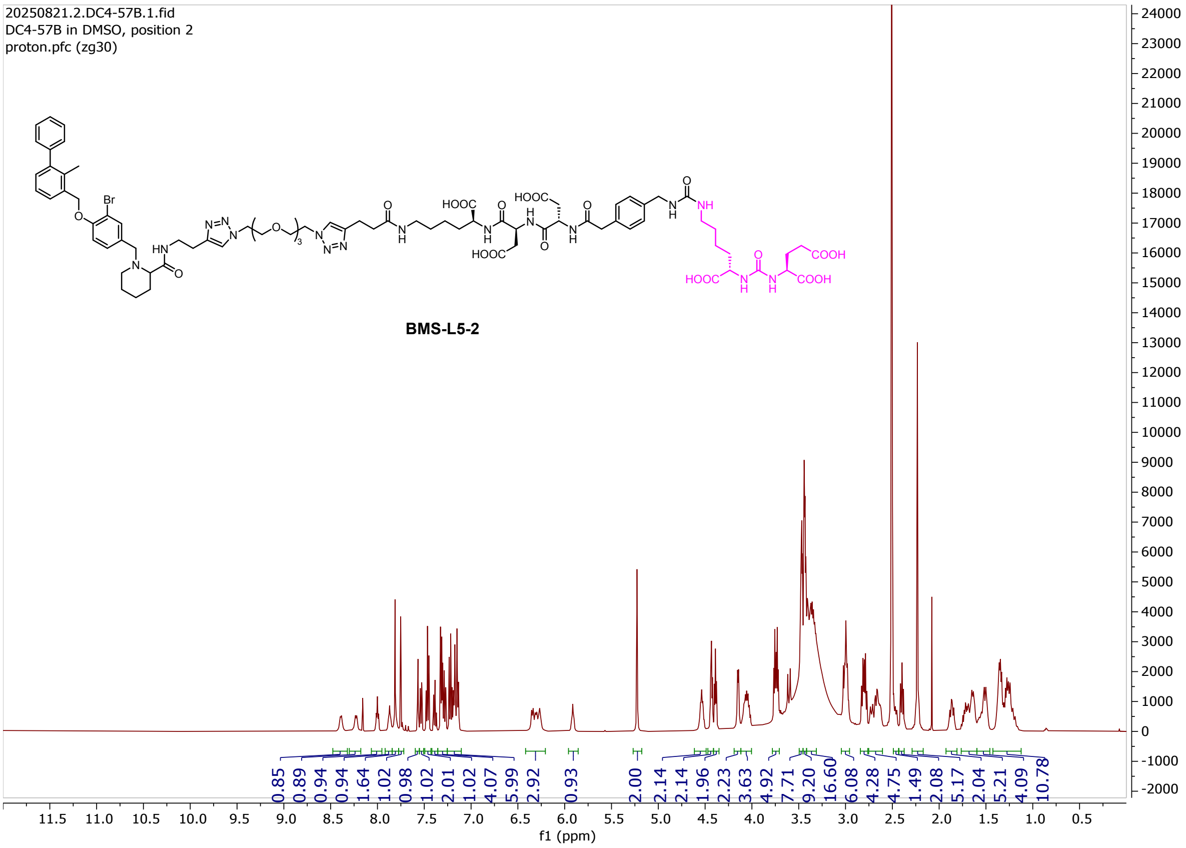
20250821.2.DC4-57B.1.fid
DC4-57B in DMSO, position 2
proton.pfc (zg30)
BMS-L5-2
f1 (ppm)

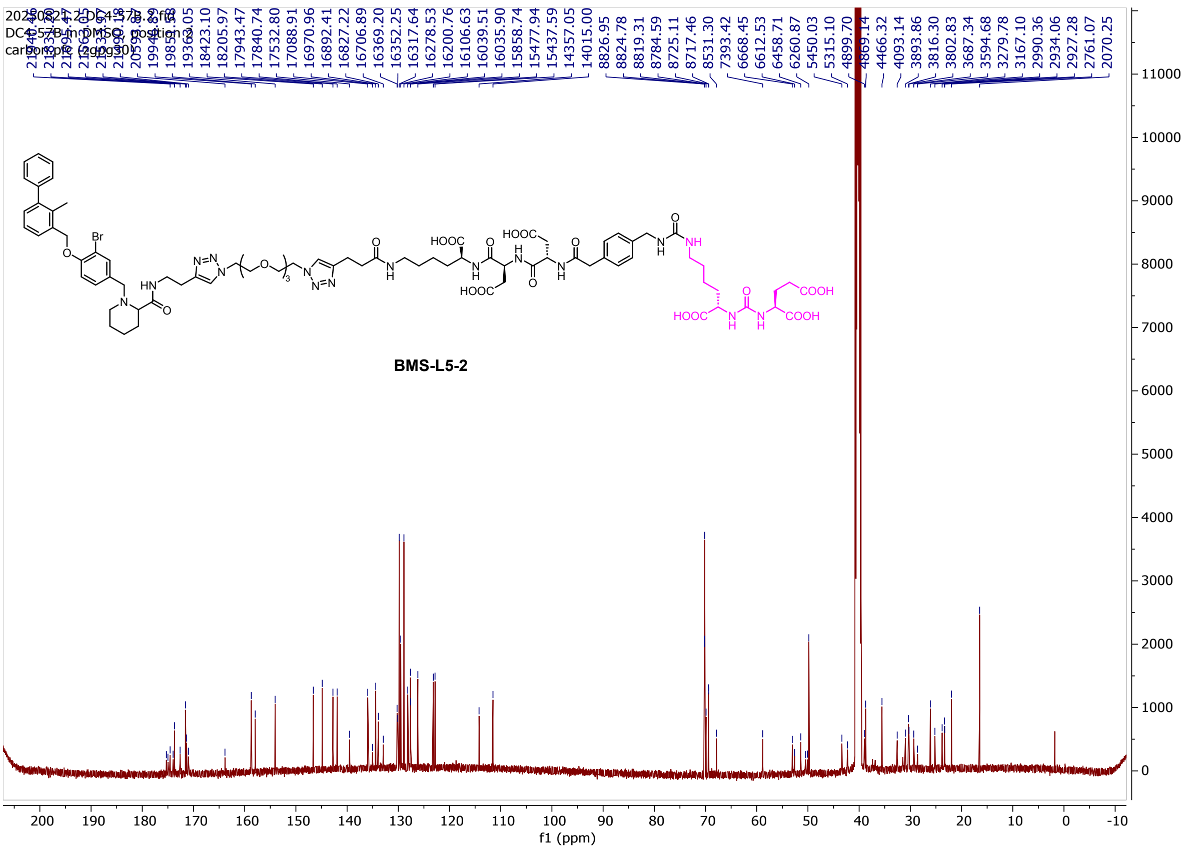
BMS-L5-2
f1 (ppm)

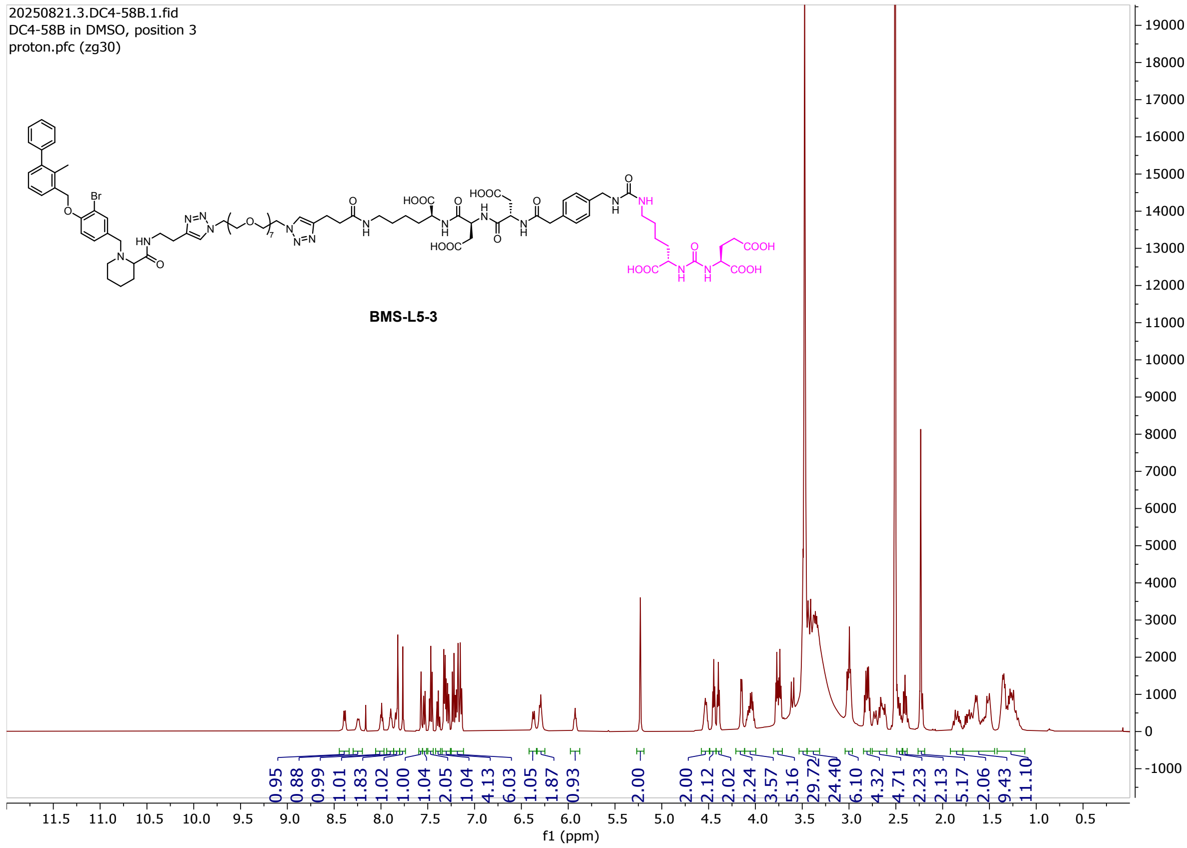
20250821.3.DC4-58B.1.fid
DC4-58B in DMSO, position 3
proton.pfc (zg30)
BMS-L5-3
f1 (ppm)

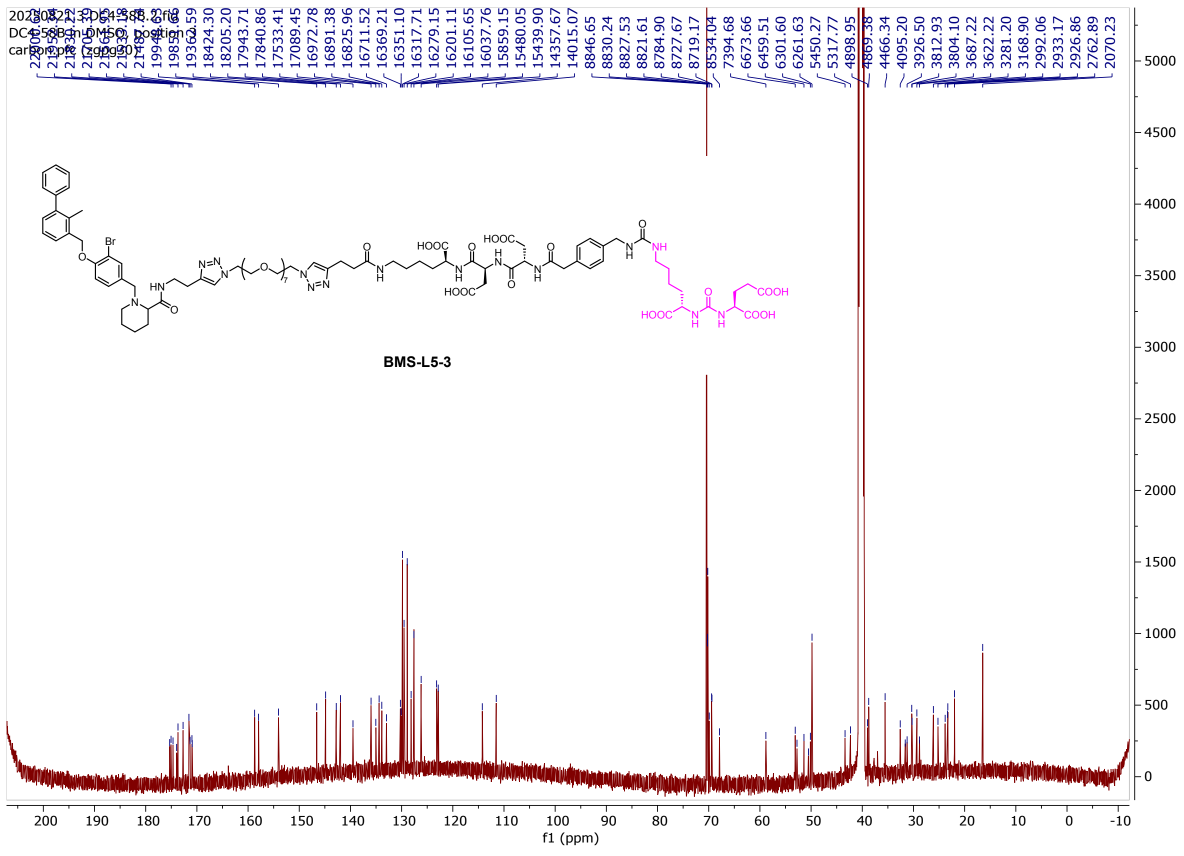
BMS-L5-3
f1 (ppm)

5. HPLC analysis

HPLC analysis of **L1-biotin**

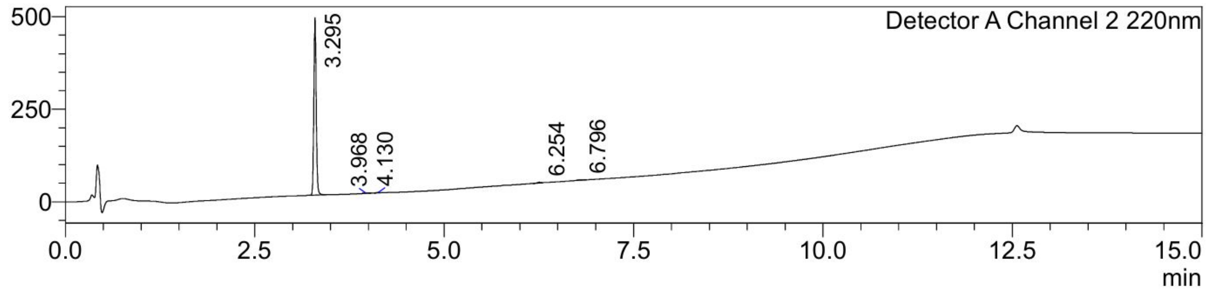

Detector A Channel 2 220nm

Peak#	Ret. Time	Height	Width at 5% Height	Area	Area%
1	3.295	468811	0.074	1049926	98.449
2	3.968	854	--	2568	0.241
3	4.130	1056	0.090	2839	0.266
4	6.254	1832	0.084	4993	0.468
5	6.796	1530	0.134	6136	0.575
Total		474083		1066462	100.000

HPLC analysis of **L2-biotin**

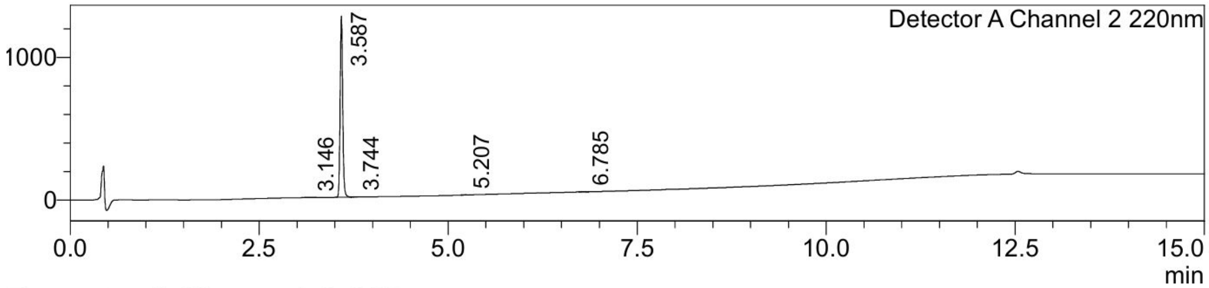

Detector A Channel 2 220nm

Peak#	Ret. Time	Height	Width at 5% Height	Area	Area%
1	3.146	1507	0.065	2657	0.088
2	3.587	1253812	0.080	3002742	99.523
3	3.744	1424	--	3992	0.132
4	5.207	852	--	1986	0.066
5	6.785	1573	--	5752	0.191
Total		1259168		3017130	100.000

HPLC analysis of **L3-biotin**

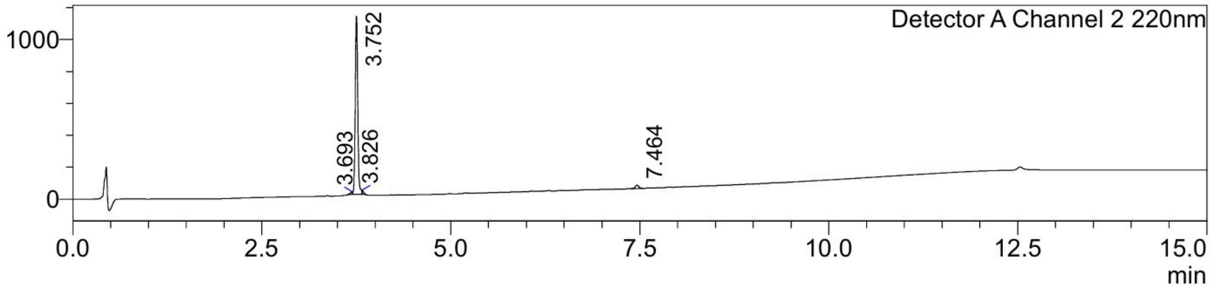

Detector A Channel 2 220nm

Peak#	Ret. Time	Height	Width at 5% Height	Area	Area%
1	3.693	15866	--	31655	1.183
2	3.752	1080192	0.078	2543357	95.030
3	3.826	26557	--	33270	1.243
4	7.464	20643	0.107	68089	2.544
Total		1143258		2676372	100.000

HPLC analysis of **L4-biotin**

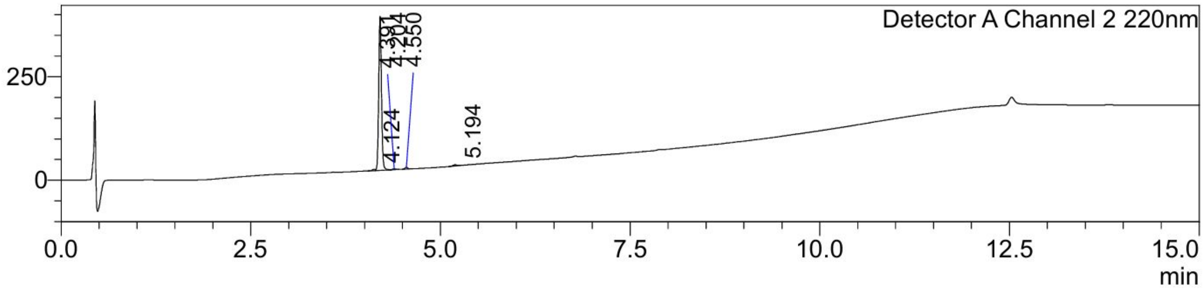

Detector A Channel 2 220nm

Peak#	Ret. Time	Height	Width at 5% Height	Area	Area%
1	4.124	3247	--	7781	0.796
2	4.204	367536	0.085	942074	96.321
3	4.391	1974	--	6200	0.634
4	4.550	4142	0.094	11355	1.161
5	5.194	2847	0.126	10644	1.088
Total		379746		978054	100.000

HPLC analysis of **L5-biotin**

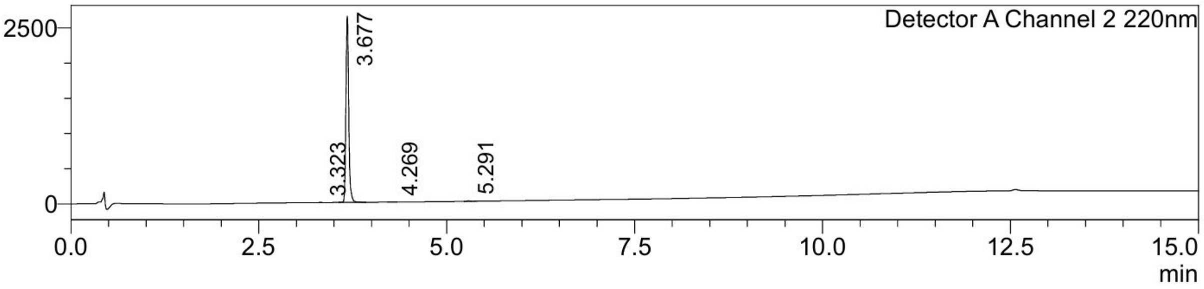

Detector A Channel 2 220nm

Peak#	Ret. Time	Height	Width at 5% Height	Area	Area%
1	3.323	2601	0.155	10975	0.159
2	3.677	2587615	0.094	6873098	99.500
3	4.269	1917	0.095	5766	0.083
4	5.291	6415	0.093	17824	0.258
Total		2598550		6907664	100.000

HPLC analysis of **L3-DBCO**

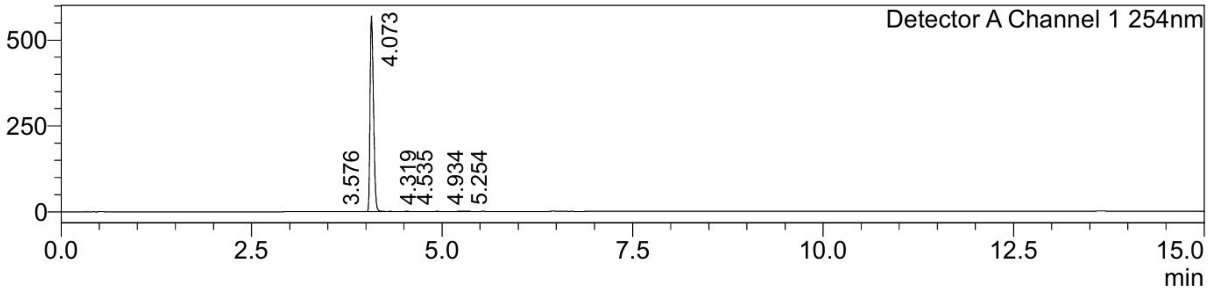

Detector A Channel 1 254nm

Peak#	Ret. Time	Height	Width at 5% Height	Area	Area%
1	3.576	181	--	703	0.039
2	4.073	563522	0.102	1787720	99.493
3	4.319	508	0.064	1045	0.058
4	4.535	1024	0.085	2442	0.136
5	4.934	419	0.081	1248	0.069
6	5.254	605	0.190	3679	0.205
Total		566260		1796838	100.000

HPLC analysis of **L5-DBCO**

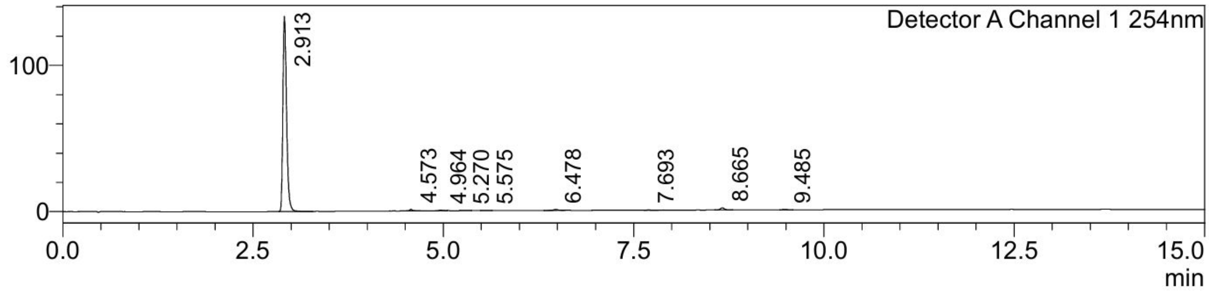

Detector A Channel 1 254nm

Peak#	Ret. Time	Height	Width at 5% Height	Area	Area%
1	2.913	132171	0.116	447635	97.169
2	4.573	995	0.088	2433	0.528
3	4.964	427	0.084	1078	0.234
4	5.270	125	0.071	281	0.061
5	5.575	173	0.123	775	0.168
6	6.478	652	0.121	2496	0.542
7	7.693	100	0.093	274	0.059
8	8.665	1408	0.123	5121	1.112
9	9.485	148	0.133	584	0.127
Total		136199		460676	100.000

HPLC analysis of **BMS-L5-1**

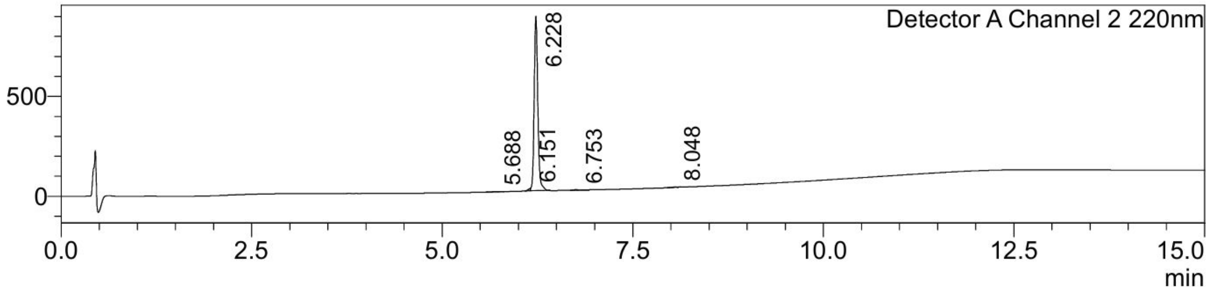

Detector A Channel 2 220nm

Peak#	Ret. Time	Height	Width at 5% Height	Area	Area%
1	5.688	1448	0.171	6015	0.205
2	6.151	10521	--	24391	0.833
3	6.228	864487	0.116	2881797	98.417
4	6.753	2168	0.132	8596	0.294
5	8.048	1245	--	7357	0.251
Total		879870		2928157	100.000

HPLC analysis of **BMS-L5-2**

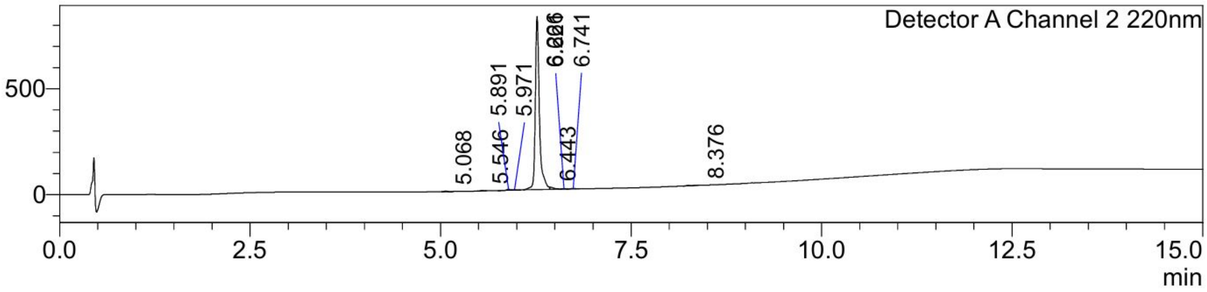

Detector A Channel 2 220nm

Peak#	Ret. Time	Height	Width at 5% Height	Area	Area%
1	5.068	2926	0.087	7570	0.240
2	5.546	2959	0.078	7328	0.233
3	5.891	4789	--	14856	0.471
4	5.971	1470	--	3438	0.109
5	6.266	802943	0.159	3065650	97.272
6	6.443	9255	--	33112	1.051
7	6.621	1843	0.074	4598	0.146
8	6.741	3163	0.117	11734	0.372
9	8.376	46	--	3334	0.106
Total		829393		3151620	100.000

HPLC analysis of **BMS-L5-3**

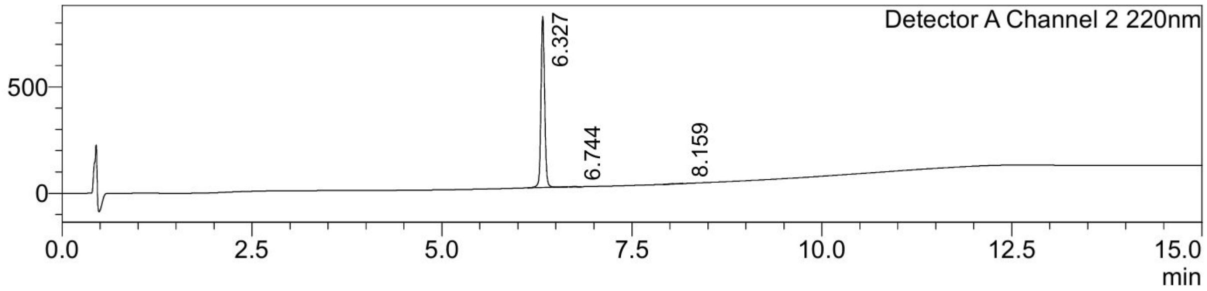

Detector A Channel 2 220nm

Peak#	Ret. Time	Height	Width at 5% Height	Area	Area%
1	6.327	787658	0.115	2696686	99.382
2	6.744	1984	0.118	7201	0.265
3	8.159	4	--	9562	0.352
Total		789646		2713449	100.000

6. References

(1) d'Orchymont, F.; Holland, J. P. Supramolecular Rotaxane-Based Multi-Modal Probes for Cancer Biomarker Imaging. *Angew. Chem. Int. Ed.* **2022**, *61* (29), e202204072.

(2) Leamon, C. P.; Reddy, J. A.; Bloomfield, A.; Dorton, R.; Nelson, M.; Vetzel, M.; Kleindl, P.; Hahn, S.; Wang, K.; Vlahov, I. R. Prostate-Specific Membrane Antigen-Specific Antitumor Activity of a Self-Immolative Tubulysin Conjugate. *Bioconjug. Chem.* **2019**, *30* (6), 1805-1813.
